# Gold(I)-catalyzed formation of furans by a Claisen-type rearrangement of ynenyl allyl ethers

**DOI:** 10.3762/bjoc.7.100

**Published:** 2011-06-29

**Authors:** Florin M Istrate, Fabien Gagosz

**Affiliations:** 1 Département de Chimie, UMR 7652, CNRS/Ecole Polytechnique, 91128 Palaiseau, France

**Keywords:** Claisen rearrangement, furans, gold-catalysis, quaternary centers

## Abstract

A series of ynenyl allyl ethers were rearranged into polysubstituted furans in the presence of a gold(I) catalyst. It is proposed that the transformation involves a Claisen-type rearrangement that allows the efficient creation of quaternary centers under mild experimental conditions.

## Findings

**Scheme 1:**
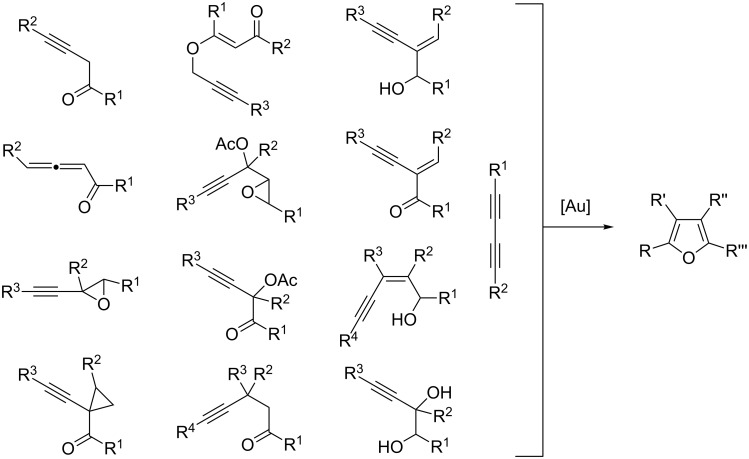
Alkynyl and allenyl substrates in gold-catalyzed formation of furans.

Furans represent an important class of heteroaromatic compounds, which are found in a large number of natural products, in synthetic biologically active substances and also in flavor chemicals [1–2]. Consequently, many efforts have been devoted to the development of synthetic methods which allow a rapid, efficient, and selective access to the furan motif [3–6]. Recently, several new strategies that involve a metal-mediated cyclization of an allene or an alkyne derivative with an oxygen functionality have appeared in the literature [7]. Among the transition metals that are commonly employed in these transformations (viz. Cu, Ag, Pd and Au), gold has proven to be particularly suitable given the strong π Lewis acidic property of cationic gold species and their ability to activate alkynes and allenes towards the addition of oxygen functionalities [8–16]. The various alkynyl and allenyl compounds presented in Scheme 1 have thus proved to be suitable precursors for the formation of polysubstituted furans in the presence of a gold(I) or a gold(III) catalyst [17–44].

We report herein our own investigations in this field which have led to the development of a new procedure for the synthesis of polysubstituted furans by a gold-catalyzed cycloisomerization of ynenyl allyl ethers [45–48].

**Scheme 2:**
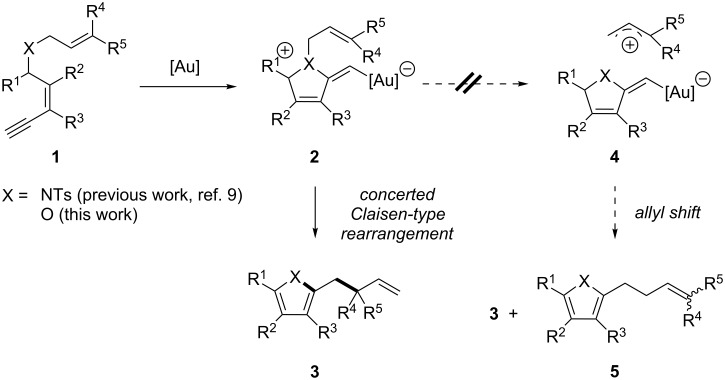
Synthetic approach to functionalized furans.

In the course of our work on the development of new gold-catalyzed transformations [49–51], we recently found that a series of ynenyl allyl tosylamides **1** (X = NTs) could be converted under mild experimental conditions into functionalized pyrroles **3** (X = NTs) in the presence of a gold(I) catalyst (Scheme 2) [52]. In contrast to Fürstners observations for the rearrangement of allyl pent-4-ynyl ethers [53–54], the results obtained during this study strongly suggested that no allyl cation was formed during the reaction. The substitution pattern of the pyrroles thus obtained point toward the involvement of a more concerted aza-Claisen-type rearrangement mechanism (**2** → **3**) and tend to exclude the possibility of a simple N to C allyl shift (**2** → **4** → **5**). Based on these initial findings, we envisaged that an analogous transformation could be employed for the synthesis of substituted furans **3** (X = O) from ynenyl allyl ethers **1** (X = O) (Scheme 2). The proof that a similar reaction can take place via an analogous pathway using oxygen- instead of nitrogen-derivatives would therefore support our initial mechanistic proposal and would broaden the scope of this new gold-catalyzed Claisen-type rearrangement [55–62].

**Figure 1:**
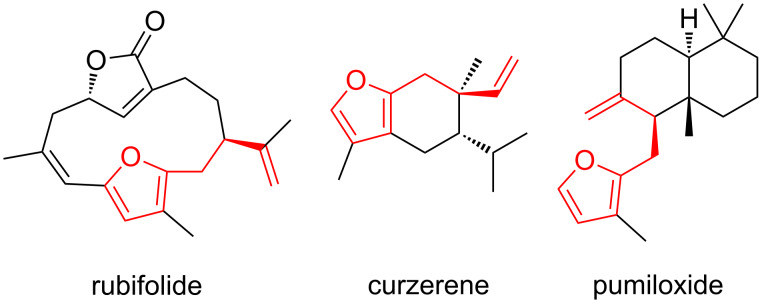
Natural products possessing a 2-butenylfuran motif.

Moreover this synthetic approach to furans would be particularly interesting for several reasons:

• The required ynenyl allyl ether substrates are easily accessible via various methods (see Supporting Information File 1 for more details),

• the Claisen-type rearrangement would allow the formation of two new C–O and C–C bonds in a single step,

• the reaction would allow the easy formation of quaternary centers and the introduction of a variety of other substituents on the side chain (when R^4^ ≠ H and R^5^ ≠ H),

• and the reaction could be particularly useful for the preparation of 2-butenylfurans, whose motif can be found in a variety of natural products, such as rubifolide [63], curzerene [64] or pumiloxide [65] (Figure 1).

Thus, a wide range of ynenyl allyl ethers **6a**–**s** was synthesized (see Supporting Information File 1) and reacted under the conditions that were found to be optimal for the analogous formation of pyrroles from ynenyl allyl tosylamides, that is, 2 mol % of the gold catalyst {[(*p*-CF_3_-C_6_H_4_)_3_P]-Au-NTf_2_} [66] in dichloromethane at room temperature (Table 1).

**Table 1:** Scope of the gold(I)-catalyzed formation of furans.^a^

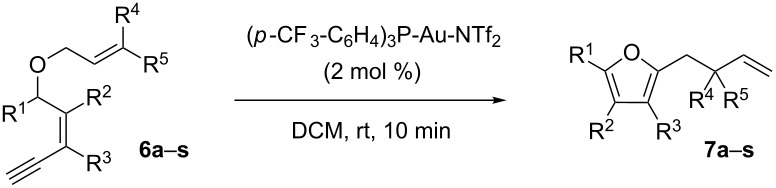						

entry	substrate		product		conversion^b^	yield^c^

1	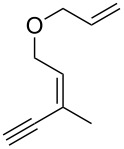	**6a**	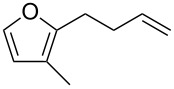	**7a**	100%	18% (75%^d^)
2	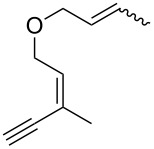	**6b**^e^	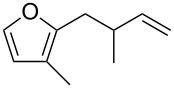	**7b**	100%	39% (86%^d^)
3	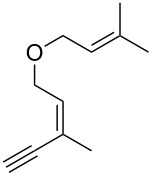	**6c**	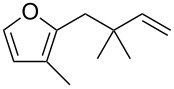	**7c**	100%	59% (82%^d^)
4	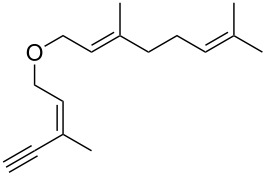	**6d**	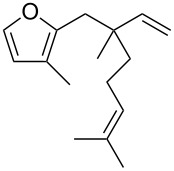	**7d**	100%	81% (92%^d^)
5	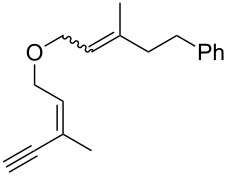	**6e**^f^	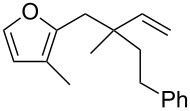	**7e**	100%	66%
6	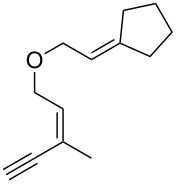	**6f**	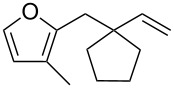	**7f**	100%	71%
7	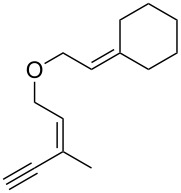	**6g**	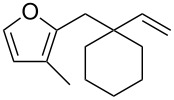	**7g**	100%	63%
8	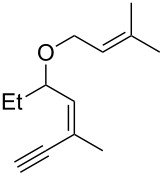	**6h**	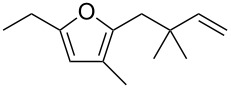	**7h**	100%	quant.
9	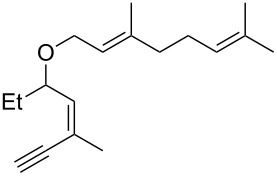	**6i**	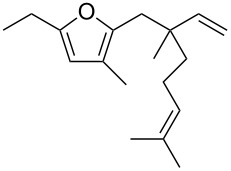	**7i**	100%	quant.
10	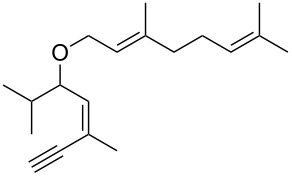	**6j**	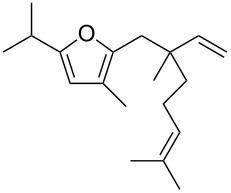	**7j**	100%	78%
11	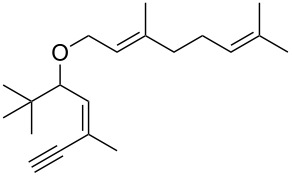	**6k**	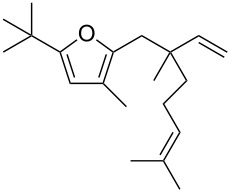	**7k**	100%	78%
12	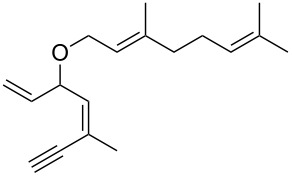	**6l**	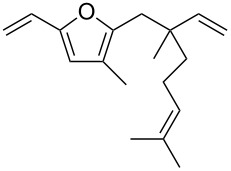	**7l**	100%	17%
13	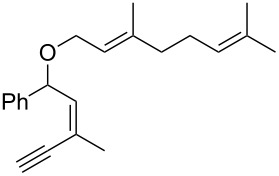	**6m**	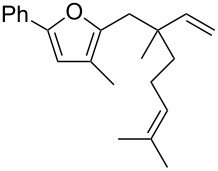	**7m**	100%	77%
14	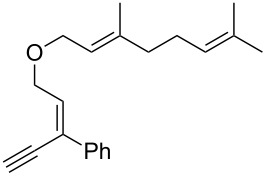	**6n**	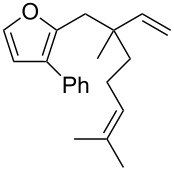	**7n**	100%	80%
15	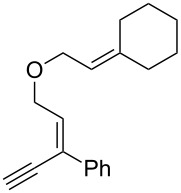	**6o**	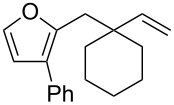	**7o**	100%	90%
16	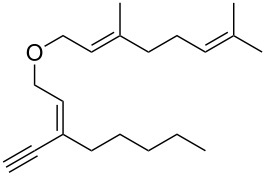	**6p**	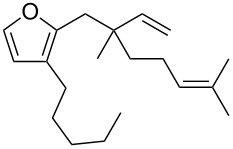	**7p**	100%	82%
17	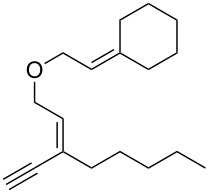	**6q**	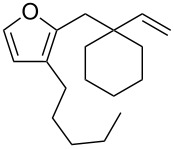	**7q**	100%	86%
18	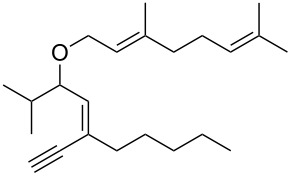	**6r**	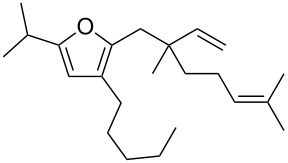	**7r**	>84%^g^	73%
19	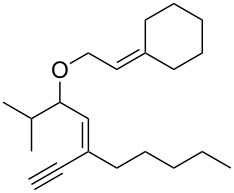	**6s**	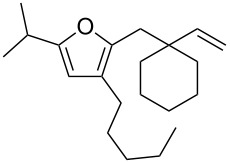	**7s**	>62%^g^	36%

^a^Reaction conditions: 0.1 M of substrate in DCM with 2 mol % of (*p*-CF_3_-C_6_H_4_)_3_P-Au-NTf_2_ at rt for 10 minutes. ^b^Conversion of the substrate determined by ^1^H NMR of the crude mixture. ^c^Isolated yields. ^d^Yields determined by ^1^H NMR of the crude mixture (with 1,3,5-trimethoxybenzene as an internal reference). ^e^*Z*/*E* ratio ≈ 1/3. ^f^*Z*/E ratio ≈ 1/2.6. ^g^Reaction time: 40 minutes.

Under these conditions, we observed the rapid formation (usually less than 10 minutes) of the expected furans. The allyl (**6a**), crotyl (**6b**), prenyl (**6c**) and geranyl (**6d**) derivatives were readily cycloisomerized in the presence of the gold catalyst, but the isolation of the corresponding furans **7a**–**d** proved to be quite challenging due to their high volatility (entries 1–4). These reactions were therefore performed in deuterated dichloromethane and their yields assessed by ^1^H NMR spectroscopy with 1,3,5-trimethoxybenzene as an internal reference (75–92%). All the examples presented in entries 2–19 are in agreement with the postulated Claisen-type rearrangement since only the exclusive formation of branched products of type **3** was observed. Indeed, a linear product of type **5** resulting from an O to C shift of the allylic moiety could not be detected, whatever substrate was used [67]. Substrates **6b** and **6e**, which were used as a mixture of *Z*/*E* isomers, each afforded a single product, i.e., the furans **7b** and **7e**, respectively (entries 2 and 5). The cycloisomerization of compounds **6f**, **6g**, **6o, 6q** and **6s**, which possess an exocyclic allyl moiety, furnished the corresponding furans **7f**, **7g**, **7o, 7q** and **7s** in moderate to quantitative yields (entries 6, 7, 15, 17 and 19). It is also worth noting that an increase in the substitution at the terminus of the allylic moieties of the substrates (monosubstitution in the case of **6b**, disubstitution for **6c**–**s**) did not notably influence the conversion, the rate or the yield of the reaction, even though the steric hindrance of the postulated Claisen intermediate **2** would have increased. This behavior strongly contrasts with the generally less efficient Claisen reactions of similarly substituted substrates and consequently allows the easy creation of a new quaternary center for the disubstituted substrates **6c**–**s** (entries 3–19). Interestingly substrates **6h**–**m**, which possess an extra substituent at the allylic position of the ynenyl fragment, also easily rearranged to afford the expected furans **7h**–**m** in good to quantitative yields (entries 8–13). A large variety of substituents were tolerated including primary, secondary or tertiary alkyl groups and even a vinyl or a phenyl group. However, a poor yield (17%) was obtained when compound **6l** was used as the substrate, due to the facile polymerization of the corresponding vinylfuran **7l** (entry 12). Substituents other than a simple methyl group could be introduced at position C(3) of the furans. Substrates **6n**–**q**, which possess either a phenyl or a longer alkyl chain, were indeed efficiently converted into compounds **7n**–**q** (80–90%, entries 14–17). However, limited reactivity was observed with ethers **6r**–**s**, which could not be completely converted into the corresponding furans **7r**–**s** (entries 18–19).

**Scheme 3:**
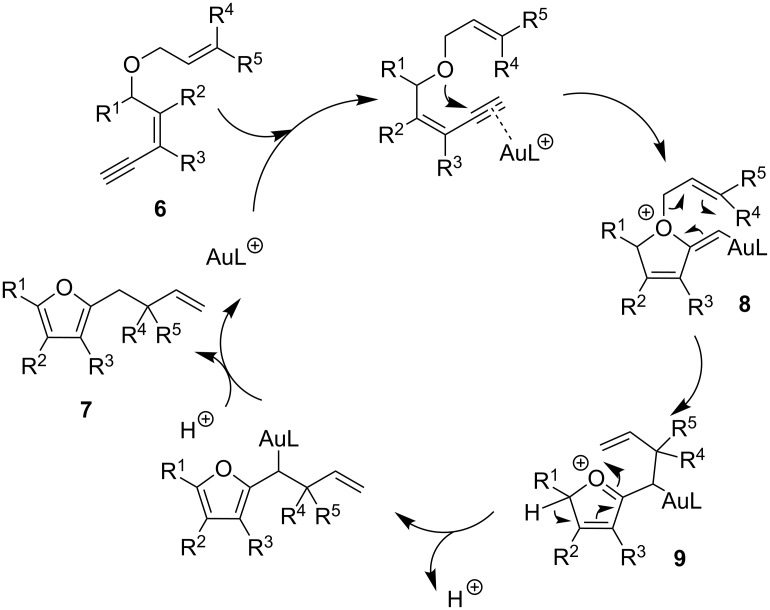
Mechanistic proposal.

A mechanistic proposal for the formation of furans **7a**–**s** is presented in Scheme 3. It is based on the results shown in entries 2–19 (Table 1), which support the involvement of a gold-catalyzed Claisen-type rearrangement as the key step of the transformation.

The gold(I) activation of the alkyne moiety in substrate **6** could promote the nucleophilic addition of the oxygen atom, and lead to the formation of the cationic vinyl gold intermediate **8**. A subsequent Claisen-type rearrangement would furnish the intermediate **9**. The loss of a proton to allow aromatization of the system, followed by a protodemetalation step would finally give furan **7**.

In summary, we have developed a new gold(I)-catalyzed formation of polysubstituted furans, which is characterized by its efficiency, the mild conditions employed and the easy formation of quaternary centers. The selectivity observed in the structure of the final product is in agreement with the postulated Claisen-type rearrangement. Further studies related to the development of an asymmetric version of this new gold(I)-catalyzed process and its application to the synthesis of natural products are underway.

## Supporting Information

File 1 Detailed experimental procedures.

File 2 NMR spectral data for substrates **6a**–**s**.

File 3 NMR spectral data for products **7a**–**s**.
