# Supplementary material for: Gold(I)-catalyzed formation of furans by a Claisen-type rearrangement of ynenyl allyl ethers
Source: Beilstein J Org Chem. 2011 Jun 29;7:878–85. doi: 10.3762/bjoc.7.100 (PMC3135071; doi:10.3762/bjoc.7.100)
Supplement: File 1 — Detailed experimental procedures. [file Beilstein_J_Org_Chem-07-878-s001.pdf]

Supporting Information

for

Gold(I)-catalyzed formation of furans by a Claisen-type rearrangement of ynenyl allyl ethers

Florin M. Istrate and Fabien Gagosz*

Address: Département de Chimie, UMR 7652, CNRS/Ecole Polytechnique, 91128 Palaiseau, France

Email: Fabien Gagosz - gagosz@dcso.polytechnique.fr

* Corresponding Author

General Information.. S2

Synthesis of substrates **6a-s** S2

Gold - catalyzed formation of furans **7a-s**.. S7

General Information

Commercially available reagents were used as received, without further purification. Dry THF and hexanes were obtained by distillation from Na/benzophenone, dry diethyl ether from $CaCl_2$ and then NaH, and dry CH_2Cl_2 from P_2O_5. $CDCl_3$ was distilled from P_2O_5, and stored over 4 Å Linde molecular sieves.

All products were purified by flash column chromatography using silica gel (40-63 µm) or neutral alumina (50-200 µm).

NMR spectra were recorded in $CDCl_3$ (or CD_2Cl_2) with TMS as an internal standard at ambient temperature, at 400 MHz for ^{1}H, at 100 MHz for ^{13}C and at 121.5 MHz for ^{31}P. Infrared absorption spectra were recorded in CCl_4 solution with a Fourier transform spectrophotometer. Melting points were determined by Reichert microscope apparatus and are uncorrected.

R_3P-Au-NTf_2 catalysts were synthesized as previously described [1].

Synthesis of substrates 6a-s

Substrates **6a-s** were synthesized by allylation of the corresponding alcohol. Typical procedure: NaH 60% dispersion in mineral oil (1.5 equiv) was added portionwise to a solution of the alcohol (1 equiv) in THF (0.5 M). The mixture was stirred at rt for a few minutes and then the corresponding bromide (1.2 equiv) and tetrabutylammonium iodide (0.1 equiv) were added. The mixture was allowed to react at rt and monitored periodically by TLC. Upon completion, the reaction was quenched with a saturated solution of NH_4Cl, the aqueous layer extracted twice with diethyl ether and the combined organic layers were washed with brine, dried over $MgSO_4$ and evaporated under vacuum. The crude mixture was then loaded onto a silica gel column and chromatographed with the appropriate mixture of petroleum ether and diethyl ether to give the expected product.

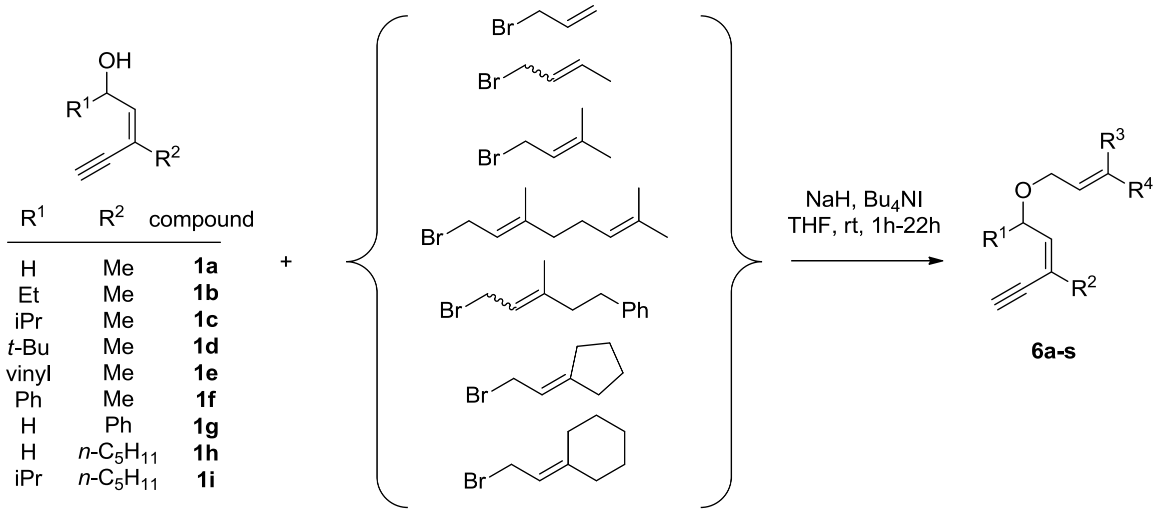

Secondary alcohols **1b-f** and **1i** were obtained by the following procedure: MnO_2 (20 equiv) was added to a solution of the starting alcohol (1 equiv) in THF (0.33 M). The mixture was stirred at rt for 2h and then filtered through a Celite pad. The filtrate was then cooled at 0°C and the corresponding organomagnesium reagent (2 equiv) was added dropwise. The mixture was allowed to warm to rt and monitored periodically by TLC. Upon completion, the reaction was quenched with a saturated solution of NH_4Cl, the aqueous layer extracted twice with diethyl ether and the combined organic layers were washed with brine, dried over $MgSO_4$ and evaporated under vacuum. The crude mixture was then loaded onto a silica gel column and chromatographed with the appropriate mixture of petroleum ether and diethyl ether to give the expected product.

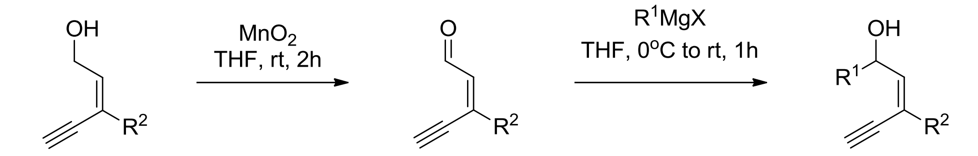

R^2	compound
Me	**1a**
n-C_5H_{11}	**1h**

R^1	R^2	compound
Et	Me	**1b**
iPr	Me	**1c**
t-Bu	Me	**1d**
vinyl	Me	**1e**
Ph	Me	**1f**
iPr	*n*-C_5H_{11}	**1i**

Alcohols **1g** and **1h** were synthesized according to the methods described in the literature [2-5].

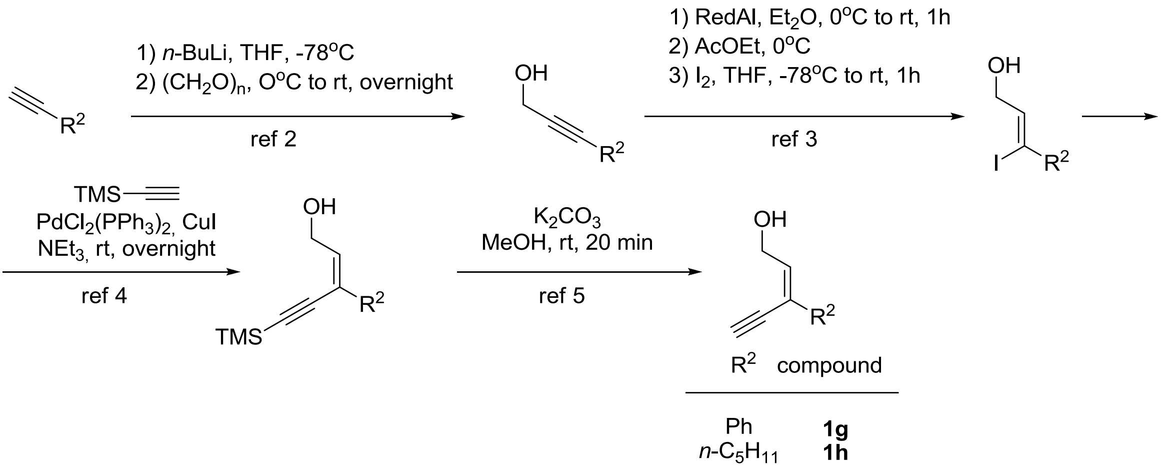

R^2	compound
Ph	**1g**
n-C_5H_{11}	**1h**

1. Mezailles, N.; Ricard, L.; Gagosz, F. *Org. Lett.* **2005**, *7*, 4133-4136.
2. Zachová, H.; Man, S.; Nečas, M.; Potáček, M. *Eur. J. Org. Chem.* **2005**, 2548–2557.
3. Fürstner, A.; Nagano, T. *J. Am. Chem. Soc.* **2007**, *129*, 1906-1907.
4. Marx, K.; Eberbach, W.; *Angew. Chem. Int. Ed.* **2000**, *6*, *11*, 2063-2068.
5. Musso, D. L.; Clarke, M. J.; Kelley, J. L.; Boswell, G. E.; Chen, G.; *Org. Biomol. Chem.* **2003**, *1*, 498-506.

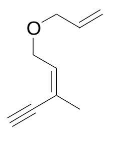

(*Z*)-5-Allyloxy-3-methylpent-3-en-1-yne (6a): Yield: 45%. Yellow oil. ^{1}H-NMR (400 MHz, $CDCl_3$): 5.96-5.87 (m, 2H), 5.28 (dd, J = 1.6, 17.2 Hz, 1H), 5.17 (dd, J = 1.4, 10.4 Hz, 1H), 4.20 (dd, J = 0.9, 6.7 Hz, 2H), 3.97 (dt, J = 1.3, 5.7 Hz, 2H), 3.15 (s, 1H), 1.90 (d, J = 1.2 Hz, 3H). ^{13}C-NMR (100 MHz, $CDCl_3$): 135.1, 134.6, 120.4, 116.9, 82.0, 81.8, 71.1, 68.2, 22.9. IR (CCl_4): 3309, 3081, 3019, 2979, 2923, 2853, 1641, 1449, 1353, 1235, 1107, 1069, 991. MS (CI+, NH_3): m/z 155 (MNH_4^+), 137 (MH^+), 105. HRMS (EI+): m/z calcd for $C_9H_{12}O$: 136.0888, found: 136.0889.

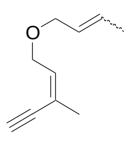

(*Z*)-5-[(But-2-enyl)oxy]-3-methylpent-3-en-1-yne (6b): Yield: 65%. Yellow oil (Z/E mixture ~ 1/3). ^{1}H-NMR (400 MHz, $CDCl_3$) for the mixture of Z/E isomers: 5.90 (m, 1H), 5.80-5.54 (m, 2H), 4.21 (dd, J = 1.0, 6.8 Hz, 2H, minor isomer), 4.19 (dd, J = 1.0, 6.7 Hz, 2H, major isomer), 4.06 (d, J = 6.3 Hz, 2H, minor isomer), 3.91 (dm, J = 6.3 Hz, 2H, major isomer), 3.16 (s, 1H, minor isomer), 3.15 (s, 1H, major isomer), 1.91 (m, 3H), 1.72 (dm, J = 6.4 Hz, major isomer), 1.68 (dm, J = 6.8 Hz, minor isomer). ^{13}C-NMR (100 MHz, $CDCl_3$): major isomer: 135.4, 129.8, 127.4, 120.2, 82.0, 81.9, 70.9, 68.1, 22.9, 17.7; minor isomer: 135.4, 127.9, 126.7, 120.3, 82.0, 81.9, 68.3, 65.5, 22.9, 13.1. IR (CCl_4): 3309, 3021, 2955, 2924, 2855, 1672, 1636, 1448, 1375, 1358, 1234, 1105, 1053, 968. MS (CI+, NH_3): m/z 169 (MNH_4^+), 151 (MH^+). HRMS (EI+): m/z calcd for $C_{10}H_{14}O$: 150.1045, found: 150.1043.

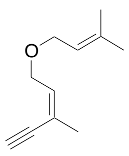

(*Z*)-3-Methyl-5-(3-methylbut-2-enyloxy)pent-3-en-1-yne (6c): Yield: 64%. Pale yellow oil. ^{1}H-NMR (400 MHz, $CDCl_3$): 5.91 (t, J = 7.0 Hz, 1H), 5.37 (t, J = 7.0 Hz, 1H), 4.18 (d, J = 6.6 Hz, 2H), 3.97 (d, J = 7.0 Hz, 2H), 3.16 (s, 1H), 1.91 (d, J = 1.2 Hz, 3H), 1.75 (s, 3H), 1.69 (s, 3H). ^{13}C-NMR (100 MHz, $CDCl_3$): 137.2, 135.6, 120.9, 120.1, 81.9, 81.9, 68.2, 66.6, 25.7, 22.9, 18.0. IR (CCl_4): 3309, 2974, 2922, 2858, 1674, 1636, 1447, 1376, 1111, 1065. MS (CI+, NH_3): m/z 183 (MNH_4^+), 165 (MH^+). HRMS (EI+): m/z calcd for $C_{11}H_{16}O$: 164.1201, found: 164.1196.

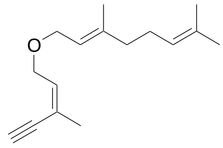

(*E*)-3,7-Dimethyl-1-((Z)-3-methylpent-2-en-4-ynyloxy)octa-2,6-diene (6d): Yield: 69%. Pale yellow oil. ^{1}H-NMR (400 MHz, $CDCl_3$): 5.91 (t, J = 6.6 Hz, 1H), 5.37 (t, J = 6.8 Hz, 1H), 5.11 (t, J = 6.6 Hz, 1H), 4.19 (d, J = 6.6 Hz, 2H), 4.00 (d, J = 6.8 Hz, 2H), 3.16 (s, 1H), 2.10 (m, 2H), 2.04 (m, 2H), 1.91 (d, J = 1.3 Hz, 3H), 1.68 (s, 3H), 1.61 (s, 3H). ^{13}C-NMR (100 MHz, $CDCl_3$): 140.4, 135.6, 131.6, 123.9, 120.6, 120.2, 81.9, 81.9, 68.2, 66.7, 39.6, 26.3, 25.6, 22.9, 17.6, 16.4. IR (CCl_4): 3309, 2964, 2923, 2857, 1668, 1447, 1377, 1108, 1062. MS (CI+, NH_3): m/z 251 (MNH_4^+), 233 (MH^+). HRMS (EI+): m/z calcd for $C_{16}H_{24}O$: 232.1827, found: 232.1834.

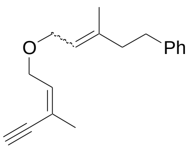

(*Z*)-5-[3-(2-Phenylethyl)but-2-enyloxy]-3-methylpent-3-en-1-yne (6e): Yield: 69%. Pale yellow oil (Z/E mixture ~ 0.28/0.72). ^{1}H-NMR (400 MHz, $CDCl_3$) for the mixture of Z/E isomers: 7.29 (m, 2H), 7.20 (m, 3H), 5.91 (m, 1H), 5.41 (m, 1H), 4.18 (dq, J = 1.1, 6.7 Hz, 2H, major isomer), 4.18 (dq, J = 1.1, 6.7 Hz, 2H, minor isomer), 4.00 (d, J = 6.7 Hz, 2H, major isomer), 3.82 (dq, J = 1.1, 0.8, 6.9 Hz, 2H, minor isomer), 3.16 (s, 1H, major isomer), 3.15 (s, 1H, minor isomer), 2.78-2.68 (m, 2H), 2.40-2.32 (m, 2H), 1.92 (d, J = 1.3 Hz, 3H, major isomer), 1.90 (d, J = 1.3 Hz, 3H, minor isomer), 1.80 (d, J = 1.1 Hz, 3H, minor isomer), 1.74 (s, 3H, major isomer). ^{13}C-NMR (100 MHz, $CDCl_3$): major isomer: 141.9, 139.8, 135.5, 128.3, 128.2, 125.8, 122.3, 120.2, 82.0, 81.9, 68.2, 66.2, 34.5, 34.3, 23.5, 22.9; minor isomer: 142.1, 139.8, 135.5, 128.3, 128.2, 125.7, 121.1, 120.2, 82.0, 81.9, 68.2, 66.6, 41.4, 34.3, 23.0, 16.6. IR (CCl_4): 3309, 3062, 3027, 2973, 2926, 2857, 1668, 1601, 1495, 1449, 1376, 1109, 1061. MS (EI+): m/z 254 (M^+), 149, 131. HRMS (EI+): m/z calcd for $C_{18}H_{22}O$: 254.1671, found: 254.1672.

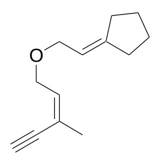

[2-((*Z*)-3-Methylpent-2-en-4-ynyloxy)ethylidene]cyclopentane (6f): Yield: 70%. Pale yellow oil. ^{1}H-NMR (400 MHz, $CDCl_3$): 5.91 (t, J = 6.6 Hz, 1H), 5.47 (m, 1H), 4.19 (dq, J = 1.0, 6.6 Hz, 2H), 3.97 (d, J = 6.9 Hz, 2H), 3.16 (s, 1H), 2.33-2.24 (m, 4H), 1.91 (d, J = 1.3 Hz, 3H), 1.73-1.58 (m, 4H). ^{13}C-NMR (100 MHz, $CDCl_3$): 148.6, 135.7, 120.0, 116.4, 82.0, 81.9, 68.2, 68.1, 33.7, 28.7, 26.2, 26.0, 22.9. IR (CCl_4): 3309, 2953, 2866, 1678, 1636, 1449, 1361, 1233, 1170, 1104, 1053. MS (EI+): m/z 190 (M^+), 161, 145, 137, 131, 121. HRMS (EI+): m/z calcd for $C_{13}H_{18}O$: 190.1358, found: 190.1363.

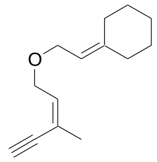

[2-((*Z*)-3-Methylpent-2-en-4-ynyloxy)ethylidene]cyclohexane (6g): Yield: 62%. Yellow oil. ^{1}H-NMR (400 MHz, $CDCl_3$): 5.91 (t, J = 6.6 Hz, 1H), 5.30 (t, J = 7.0 Hz, 1H), 4.19 (dq, J = 1.1, 6.6 Hz, 2H), 3.99 (d, J = 7.0 Hz, 2H), 3.16 (s, 1H), 2.19 (m, 4H), 2.13 (m, 2H), 1.91 (d, J = 1.3 Hz, 3H), 1.57-1.53 (m, 6H). ^{13}C-NMR (100 MHz, $CDCl_3$): 145.0, 135.7, 120.0, 117.6, 82.0, 81.9, 68.1, 65.8, 37.0, 28.9, 28.3, 27.7, 26.7, 22.9. IR (CCl_4): 3309, 2929, 2854, 1668, 1446, 1100, 1065. MS (EI+): m/z 204 (M^+), 189, 175, 161, 145, 137, 123, 109. HRMS (EI+): m/z calcd for $C_{14}H_{20}O$: 204.1514, found: 204.1505.

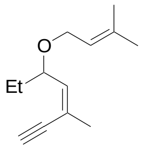

(*Z*)-3-Methyl-5-(3-methylbut-2-enyloxy)hept-3-en-1-yne (6h): Yield: 44%. Pale yellow oil. ^{1}H-NMR (400 MHz, $CDCl_3$): 5.64 (dm, J = 0.7, 9.1 Hz, 1H), 5.35 (t, J = 6.9 Hz, 1H), 4.17 (dt, J = 6.7, 9.1 Hz, 1H), 3.98 (dd, J = 6.8, 11.4 Hz, 1H), 3.88 (dd, J = 7.1, 11.3 Hz, 1H), 3.11 (s, 1H), 1.92 (d, J = 1.4 Hz, 3H), 1.74 (s, 3H), 1.67 (s, 3H), 1.64 (m, 1H), 1.50 (m, 1H), 0.92 (t, J = 7.4 Hz, 3H). ^{13}C-NMR (100 MHz, $CDCl_3$): 140.4, 136.6, 121.5, 120.1, 82.4, 81.1, 78.6, 65.0, 28.2, 25.8, 23.0, 18.0, 9.7. IR (CCl_4): 3309, 2970, 2926, 2866, 1668, 1634, 1447, 1378, 1111, 1056. MS (CI+, NH_3): m/z 211 (MNH_4^+), 193 (MH^+), 175, 137, 124. HRMS (EI+): m/z calcd for $C_{13}H_{20}O$: 192.1514, found: 192.1520.

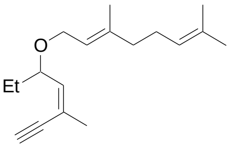

(*E*)-1-((*Z*)-1-Ethyl-3-methylpent-2-en-4-ynyloxy)-3,7-dimethylocta-2,6-diene (6i): Yield: 64%. Pale yellow oil. ^{1}H-NMR (400 MHz, $CDCl_3$): 5.64 (dm, J = 0.7, 9.1 Hz, 1H), 5.36 (tq, J = 1.0, 6.9 Hz, 1H), 5.11 (thept, J = 1.4, 6.9 Hz, 1H), 4.18 (dt, J = 6.6, 9.1 Hz, 1H), 4.03 (dd, J = 6.5, 11.6 Hz, 1H), 3.92 (dd, J = 7.1, 11.6 Hz, 1H), 3.10 (s, 1H), 2.10 (m, 2H), 2.04 (m, 2H), 1.92 (d, J = 1.5 Hz, 3H), 1.69 (s, 3H), 1.67 (m, 1H), 1.66 (s, 3H), 1.61 (s, 3H), 1.48 (m, 1H), 0.92 (t, J = 7.5 Hz, 3H). ^{13}C-NMR (100 MHz, $CDCl_3$): 140.4, 139.7, 131.5, 124.1, 121.2, 120.0, 82.5, 81.2, 78.5, 65.0, 39.6, 28.2, 26.3, 25.6, 23.1, 17.6, 16.5, 9.7. IR (CCl_4): 3309, 2970, 2927, 2872, 1674, 1632, 1448, 1377, 1110, 1059. MS (CI+, NH_3): m/z 279 (MNH_4^+), 261 (MH^+), 243, 173, 154, 137. HRMS (EI+): m/z calcd for $C_{18}H_{28}O$: 260.2140, found: 260.2144.

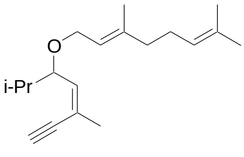

(*E*)-1-((*Z*)-1-Isopropyl-3-methylpent-2-en-4-ynyloxy)-3,7-dimethylocta-2,6-diene (6j): Yield: 50%. Yellow oil. ^{1}H-NMR (400 MHz, $CDCl_3$): 5.65 (dm, J = 0.7, 9.4 Hz, 1H), 5.35 (tq, J = 1.3, 7.0 Hz, 1H), 5.11 (thept, J = 1.4, 6.9 Hz, 1H), 4.01 (dd, J = 6.4, 11.8 Hz, 1H), 3.94 (dd, J = 9.4, 7.1 Hz, 1H), 3.90 (dd, J = 7.0, 11.7 Hz, 1H), 3.08 (d, J = 0.6 Hz, 1H), 2.10 (m, 2H), 2.04 (m, 2H), 1.94 (d, J = 1.5 Hz, 3H), 1.75 (m, 1H), 1.69 (s, 3H), 1.66 (s, 3H), 1.61 (s, 3H), 0.97 (d, J = 6.7 Hz, 3H), 0.87 (d, J = 6.8 Hz, 3H). ^{13}C-NMR (100 MHz, $CDCl_3$): 139.6, 139.1, 131.4, 124.1, 121.4, 120.7, 82.7, 82.2, 81.0, 65.1, 39.6, 32.9, 26.3, 25.6, 23.2, 18.8, 18.1, 17.6, 16.5. IR (CCl_4): 3309, 2966, 2924, 2872, 1667, 1635, 1450, 1377, 1219, 1104, 1058. MS (CI+, NH_3): m/z 275 (MH^+), 257, 248. HRMS (EI+): m/z calcd for $C_{19}H_{30}O$: 274.2297, found: 274.2287.

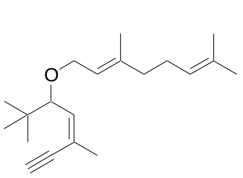

(*E*)-1-((*Z*)-1-*tert*-Butyl-3-methylpent-2-en-4-ynyloxy)-3,7-dimethylocta-2,6-diene (6k): Yield: 40%. Yellow oil. ^{1}H-NMR (400 MHz, $CDCl_3$): 5.70 (dm, J = 0.7, 9.7 Hz, 1H), 5.33 (tq, J = 1.2, 7.1 Hz, 1H), 5.12 (thept, J = 1.4, 6.9 Hz, 1H), 4.00 (dd, J = 6.1, 12.0 Hz, 1H), 3.88 (dd, J = 6.5, 12.4 Hz, 1H), 3.87 (d, J = 9.7 Hz, 1H), 3.07 (s, 1H), 2.10 (m, 2H), 2.04 (m, 2H), 1.94 (d, J = 1.4 Hz, 3H), 1.69 (s, 3H), 1.64 (s, 3H), 1.61 (s, 3H), 0.91 (s, 3H). ^{13}C-NMR (100 MHz, $CDCl_3$): 139.0, 138.0, 131.4, 124.1, 121.8, 121.0, 84.3, 82.9, 80.9, 65.4, 39.6, 35.3, 26.4, 25.9, 25.6, 23.3, 17.6, 16.5. IR (CCl_4): 3309, 2959, 2926, 2866, 1667, 1449, 1379, 1107, 1057. MS (EI+): m/z 288 (M^+), 273, 231. HRMS (EI+): m/z calcd for $C_{20}H_{23}O$: 288.2453, found: 288.2454.

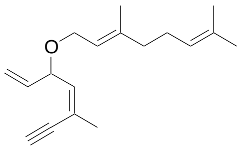

(*E*)-3,7-Dimethyl-1-((*Z*)-3-methyl-1-vinylpent-2-en-4-ynyloxy)octa-2,6-diene (6l): Yield: 57%. Yellow oil. ^{1}H-NMR (400 MHz, $CDCl_3$): 5.81 (ddd, J = 6.4, 10.4, 17.3 Hz, 1H), 5.72 (dm, J = 0.7, 8.8 Hz, 1H), 5.37 (tq, J = 1.3, 6.8 Hz, 1H), 5.30 (dt, J = 1.4, 17.2 Hz, 1H), 5.16 (ddd, J = 1.2, 1.6, 10.4 Hz, 1H), 5.11 (thept, J = 1.4, 6.9 Hz, 1H), 4.78 (dm, J = 7.7 Hz, 1H), 4.00 (d, J = 6.8 Hz, 2H), 3.16 (d, J = 0.6 Hz, 1H), 2.10 (m, 2H), 2.05 (m, 2H), 1.92 (d, J = 1.5 Hz, 3H), 1.68 (s, 3H), 1.66 (s, 3H), 1.60 (s, 3H). ^{13}C-NMR (100 MHz, $CDCl_3$): 140.1, 138.1, 137.0, 131.5, 124.0, 120.8, 120.1, 116.1, 82.1, 81.9, 78.3, 64.7, 39.6, 26.3, 25.6, 23.0, 17.6, 16.5. IR (CCl_4): 3309, 2972, 2922, 2859, 1668, 1640, 1445, 1378, 1111, 1054. MS (EI+): m/z 258 (M^+), 187, 175, 159, 145, 135, 123, 106. HRMS (EI+): m/z calcd for $C_{18}H_{26}O$: 258.1984, found: 258.1991.

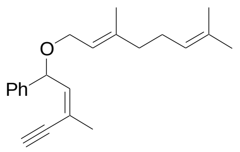

[(*Z*)-1-((*E*)-3,7-Dimethylocta-2,6-dienyloxy)-3-methylpent-2-en-4-ynyl]benzene (6m): Yield: 59%. Yellow oil. ^{1}H-NMR (400 MHz, $CDCl_3$): 7.42 (d, J = 7.1 Hz, 2H), 7.35 (t, J = 7.2 Hz, 2H), 7.27 (t, J = 7.2 Hz, 1H), 5.89 (dm, J = 0.7, 9.1 Hz, 1H), 5.42 (tq, J = 1.2, 6.8 Hz, 1H), 5.38 (d, J = 9.1 Hz, 1H), 5.12 (thept, J = 1.4, 6.9 Hz, 1H), 4.02 (t, J = 6.2 Hz, 2H), 3.21 (s, 1H), 2.10 (m, 2H), 2.06 (m, 2H), 1.90 (d, J = 1.4 Hz, 3H), 1.69 (s, 3H), 1.63 (s, 3H), 1.61 (s, 3H). ^{13}C-NMR (100 MHz, $CDCl_3$): 141.5, 140.2, 139.6, 131.5, 128.4, 127.5, 126.4, 124.0, 120.8, 119.3, 82.4, 81.8, 78.9, 64.9, 39.6, 26.4, 25.6, 23.0, 17.6, 16.5. IR (CCl_4): 3309, 2970, 2922, 2859, 1668, 1600, 1448, 1377, 1102, 1055. MS (CI+, NH_3): m/z 309 (MH^+), 171, 155. HRMS (EI+): m/z calcd for $C_{22}H_{28}O$: 308.2140, found: 308.2144.

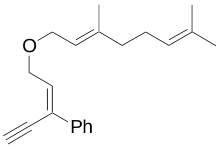

[(*Z*)-3-((*E*)-3,7-Dimethylocta-2,6-dienyloxy)-1-ethynylpropenyl]benzene (6n): Yield: 68%. Yellow oil. ^{1}H-NMR (400 MHz, $CDCl_3$): 7.64 (d, J = 7.0 Hz, 2H), 7.36 (t, J = 6.9 Hz, 2H), 7.31 (t, J = 7.0 Hz, 1H), 6.63 (t, J = 6.3 Hz, 1H), 5.41 (tq, J = 1.2, 6.8 Hz, 1H), 5.12 (thept, J = 1.3, 6.8 Hz, 1H), 4.46 (d, J = 6.4 Hz, 2H), 4.09 (d, J = 6.8 Hz, 2H), 3.41 (s, 1H), 2.12 (m, 2H), 2.06 (m, 2H), 1.71 (s, 3H), 1.69 (s, 3H), 1.62 (s, 3H). ^{13}C-NMR (100 MHz, $CDCl_3$): 140.7, 136.7, 135.9, 131.6, 128.4, 128.1, 126.0, 124.0, 123.9, 120.5, 84.5, 79.9, 68.8, 66.9, 39.6, 26.3, 25.6, 17.6, 16.5. IR (CCl_4): 3308, 2969, 2922, 2857, 1668, 1447, 1375, 1110, 1073. MS (EI+): m/z 294 (M^+), 279, 251, 225, 211, 195, 183, 167, 157, 141, 128, 115, 109. HRMS (EI+): m/z calcd for $C_{21}H_{26}O$: 294.1984., found: $C_{21}H_{26}O$: 294.1977.

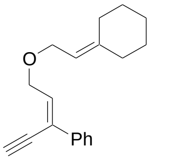

[(*Z*)-3-(2-Cyclohexylidene-ethyloxy)-1-ethynylpropenyl]benzene (6o): Yield: 77%. Yellow oil. ^{1}H-NMR (400 MHz, $CDCl_3$): 7.64 (d, J = 7.1 Hz, 2H), 7.36 (t, J = 7.0 Hz, 2H), 7.30 (t, J = 7.1 Hz, 1H), 6.62 (t, J = 6.3 Hz, 1H), 5.34 (t, J = 7.0 Hz, 1H), 4.45 (d, J = 6.4 Hz, 2H), 4.07 (d, J = 7.0 Hz, 2H), 3.41 (s, 1H), 2.21 (m, 2H), 2.15 (m, 2H), 1.58-1.53 (m, 6H). ^{13}C-NMR (100 MHz, $CDCl_3$): 145.3, 136.7, 136.1, 128.4, 128.1, 126.0, 123.9, 117.4, 84.5, 79.9, 77.0, 68.7, 66.1, 37.1, 29.0, 28.3, 27.7, 26.6. IR (CCl_4): 3308, 2930, 2854, 1711, 1668, 1495, 1446, 1360, 1239, 1085. MS (EI+): m/z 266 (M^+), 235, 223, 207, 195. HRMS (EI+): m/z calcd for $C_{19}H_{22}O$: 266.1671, found: 266.1675.

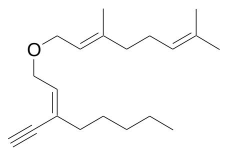

(*E*)-1-((*Z*)-3-Ethynyloct-2-enyloxy)-3,7-dimethylocta-2,6-diene (6p): Yield: 84%. Yellow oil. ^{1}H-NMR (400 MHz, $CDCl_3$): 5.90 (t, J = 6.5 Hz, 1H), 5.37 (tq, J = 1.2, 6.8 Hz, 1H), 5.11 (thept, J = 1.4, 6.9 Hz, 1H), 4.22 (d, J = 6.5 Hz, 2H), 4.00 (d, J = 6.8 Hz, 2H), 3.16 (s, 1H), 2.18-2.01 (m, 6H), 1.69 (s, 3H), 1.68 (s, 3H), 1.61 (s, 3H), 1.54 (m, 2H), 1.36-1.26 (m, 4H), 0.90 (t, J = 7.1 Hz, 3H). ^{13}C-NMR (100 MHz, $CDCl_3$): 140.4, 135.0, 131.6, 125.1, 124.0, 120.7, 82.4, 81.3, 77.0, 68.1, 66.6, 39.6, 36.8, 31.1, 27.6, 26.3, 25.6, 22.4, 17.6, 16.4, 13.9. IR (CCl_4): 3309, 2957, 2928, 2858, 1668, 1633, 1449, 1376, 1100, 1064. MS (EI+): m/z 288 (M^+), 273, 257, 245, 230, 217, 205. HRMS (EI+): m/z calcd for $C_{20}H_{32}O$: 288.2453, found: 288.2458.

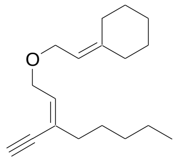

[2-((*Z*)-3-Ethynyloct-2-enyloxy)ethylidene]cyclohexane (6q): Yield: 75%. Yellow oil. ^{1}H-NMR (400 MHz, $CDCl_3$): 5.91 (t, J = 6.5 Hz, 1H), 5.30 (t, J = 7.0 Hz, 1H), 4.21 (d, J = 6.5 Hz, 2H), 3.99 (d, J = 7.0 Hz, 2H), 3.16 (s, 1H), 2.21-2.10 (m, 6H), 1.57-1.50 (m, 8H), 1.36-1.24 (m, 4H), 0.90 (t, J = 7.1 Hz, 3H). ^{13}C-NMR (100 MHz, $CDCl_3$): 145.0, 135.1, 125.0, 117.6, 82.4, 81.4, 68.1, 65.8, 37.0, 36.8, 31.1, 28.9, 28.3, 27.7, 27.6, 26.7, 22.4, 13.9. IR (CCl_4): 3309, 2930, 2856, 1668, 1447, 1373, 1233, 1091, 1050. MS (EI+): m/z 260 (M^+), 245, 242, 229, 217, 204. HRMS (EI+): m/z calcd for $C_{18}H_{28}O$: 260.2140, found: 260.2147.

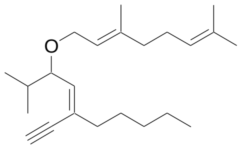

(*Z*)-3-((*E*)-3,7-Dimethylocta-2,6-dienyloxy)-5-ethynyl-2-methyldec-4-ene (6r): Yield: 50%. Yellow oil. ^{1}H-NMR (400 MHz, $CDCl_3$): 5.64 (dd, J = 0.7, 9.4 Hz, 1H), 5.35 (tq, J = 0.7, 7.0 Hz, 1H), 5.11 (thept, J = 1.4, 6.9 Hz, 1H), 4.03 (dd, J = 6.4, 12.1 Hz, 1H), 3.98 (dd, J = 7.1, 9.4 Hz, 1H), 3.89 (dd, J = 7.0, 12.1 Hz, 1H), 3.08 (d, J = 0.6 Hz, 1H), 2.19 (td, J = 0.7, 7.5 Hz, 2H), 2.10 (m, 2H), 2.03 (m, 2H), 1.76 (hept, J = 6.7 Hz, 1H), 1.69 (s, 3H), 1.65 (s, 3H), 1.61 (s, 3H), 1.54 (m, 2H), 1.35-1.28 (m, 4H), 0.98 (d, J = 6.7 Hz, 3H), 0.90 (t, J = 6.9 Hz, 3H), 0.87 (d, J = 6.8 Hz, 3H). ^{13}C-NMR (100 MHz, $CDCl_3$): 139.6, 138.5, 131.4, 126.0, 124.1, 121.4, 82.2, 82.1, 81.5, 65.1, 39.6, 37.0, 32.9, 31.0, 27.8, 26.3, 25.6, 22.4, 18.9, 18.1, 17.6, 16.5, 14.0. IR (CCl_4): 3309, 2960, 2928, 2863, 1457, 1378, 1114, 1058. MS (EI+): m/z 330 (M^+), 315, 292, 288, 259, 247, 230, 218, 204. HRMS (EI+): m/z calcd for $C_{23}H_{38}O$: 330.2923, found: 330.2915.

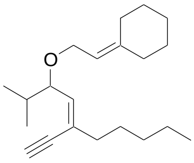

[2-((*Z*)-3-Ethynyl-1-isopropyl-oct-2-enyloxy)ethylidene]cyclohexane (6s): Yield: 40%. Yellow oil. ^{1}H-NMR (400 MHz, $CDCl_3$): 5.63 (dd, J = 0.7, 9.4 Hz, 1H), 5.29 (t, J = 6.9 Hz, 1H), 4.01 (dd, J = 6.7, 11.4 Hz, 1H), 3.95 (dd, J = 7.1, 9.4 Hz, 1H), 3.87 (dd, J = 7.1, 11.5 Hz, 1H), 3.08 (d, J = 0.7Hz, 1H), 2.20-2.06 (m, 6H), 1.76 (hept, J = 6.9 Hz, 1H), 1.58-1.51 (m, 8H), 1.36-1.26 (m, 4H), 0.98 (d, J = 6.7 Hz, 3H), 0.91 (t, J = 6.9 Hz, 3H), 0.87 (d, J = 6.8 Hz, 3H). ^{13}C-NMR (100 MHz, $CDCl_3$): 144.2, 138.6, 125.9, 118.3, 82.3, 82.1, 81.5, 64.3, 37.1, 37.0, 32.9, 31.0, 29.0, 28.3, 27.8, 27.7, 26.7, 22.4, 18.9, 18.1, 14.0. IR (CCl_4): 3309, 2928, 2857, 1705, 1667, 1448, 1377, 1066, 1049. MS (EI+): m/z 302 (M^+), 287, 280, 272, 266, 259, 246, 229, 215, 204. HRMS (EI+): m/z calcd for $C_{21}H_{34}O$: 302.2610, found: 302.2606.

Gold-catalyzed formation of furans 7a-s

General procedure: To a solution of the substrate (0.25 mmoles, 1 equiv) in CH_2Cl_2 (0.1 M) was added (p-CF_3-C_6H_4)$_3$P-Au-NTf_2 (4.7 mg, 0.02 equiv). The mixture was stirred at rt and monitored periodically by TLC. Upon completion, the mixture was evaporated, loaded onto a silica gel column and chromatographed with petroleum ether to give the desired furan.

Procedure for catalysis reactions in deuterated solvent with internal reference: (*p*-CF_3-C_6H_4)$_3$P-Au-NTf_2 (1.9 mg, 0.02 equiv) and 1,3,5-trimethoxybenzene (17 mg, 1 equiv) was added to a solution of the substrate (0.1 mmoles, 1 equiv) in CD_2Cl_2 (0.1M). The mixture was stirred at rt and monitored periodically by NMR. The yield of the desired product was assessed by NMR.

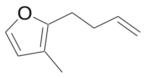

2-But-3-enyl-3-methylfuran (7a): Isolated yield: 18%. Pale yellow oil. ^{1}H-NMR (400 MHz, $CDCl_3$): 7.23 (d, J = 1.7 Hz, 1H), 6.16 (d, J = 1.6 Hz, 1H), 5.84 (ddt, J = 6.6, 10.1, 17.1 Hz, 1H), 5.05 (dd, J = 1.4, 17.1 Hz, 1H), 4.98 (d, J = 10.2 Hz, 1H), 2.66 (t, J = 7.2 Hz, 2H), 2.36 (dt, J = 6.7, 8.0 Hz, 2H), 1.97 (s, 3H). ^{13}C-NMR (100 MHz, $CDCl_3$): 150.4, 139.7, 137.7, 115.0, 113.8, 112.6, 32.5, 25.5, 15.2. IR (CCl_4): 2926, 2857, 1640, 1451, 1150. MS (CI+, NH_3): m/z 137 (MH^+), 122, 105. HRMS (EI+): m/z calcd for $C_9H_{12}O$: 136.0888, found: 136.0882.

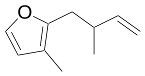

3-Methyl-2-(2-methylbut-3-enyl)furan (7b): Isolated yield: 39%. Pale yellow oil. ^{1}H-NMR (400 MHz, $CDCl_3$): 7.24 (d, J = 1.8 Hz, 1H), 6.17 (d, J = 1.7 Hz, 1H), 5.80 (ddd, J = 6.6, 10.4, 17.2 Hz, 1H), 4.99 (dt, J = 1.4, 17.2 Hz, 1H), 4.94 (dt, J = 0.8, 10.3 Hz, 1H), 2.64-2.48 (m, 2H), 1.96 (s, 3H), 1.00 (d, J = 6.4 Hz, 3H). ^{13}C-NMR (100 MHz, $CDCl_3$): 149.8, 143.6, 139.8, 114.7, 112.7, 112.5, 37.2, 33.0, 19.2, 9.9. IR (CCl_4): 2960, 2926, 2867, 1640, 1512, 1456, 1374, 1149. MS (CI+, NH_3): m/z 169 (MNH_4^+), 151 (MH^+), 137. HRMS (EI+): m/z calcd for $C_{10}H_{14}O$: 150.1045, found: 150.1048.

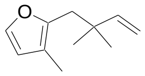

2-(2,2-Dimethylbut-3-enyl)-3-methylfuran (7c): Isolated yield: 59%. Pale yellow oil. ^{1}H-NMR (400 MHz, $CDCl_3$): 7.24 (d, J = 1.7 Hz, 1H), 6.18 (d, J = 1.6 Hz, 1H), 5.91 (dd, J = 10.7, 17.5 Hz, 1H), 4.94 (dd, J = 1.2, 17.5 Hz, 1H), 4.91 (dd, J = 1.2, 10.7 Hz, 1H), 2.54 (s, 2H), 1.96 (s, 3H), 1.04 (s, 6H). ^{13}C-NMR (100 MHz, $CDCl_3$): 149.4, 148.1, 139.9, 115.7, 112.5, 110.1, 38.7, 38.5, 26.5, 10.4. IR (CCl_4): 2961, 2927, 2869, 1639, 1511, 1460, 1377, 1150, 1075, 1001. MS (CI+, NH_3): m/z 183 (MNH_4^+), 165 (MH^+). HRMS (EI+): m/z calcd for $C_{11}H_{16}O$: 164.1201, found: 164.1205.

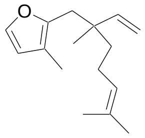

2-(2,6-Dimethyl-2-vinylhept-5-enyl)-3-methylfuran (7d): Isolated yield: 81%. Yellow oil. ^{1}H-NMR (400 MHz, $CDCl_3$): 7.24 (d, J = 1.8 Hz, 1H), 6.18 (d, J = 1.7 Hz, 1H), 5.84 (dd, J = 10.8, 17.5 Hz, 1H), 5.10 (thept, J = 1.4, 7.1 Hz, 1H), 5.01 (dd, J = 1.3, 10.8 Hz, 1H), 4.93 (dd, J = 1.3, 17.5 Hz, 1H), 2.57 (s, 2H), 1.96 (s, 3H), 1.93 (m, 2H), 1.69 (s, 3H), 1.61 (s, 3H), 1.36 (m, 2H), 1.03 (s, 3H). ^{13}C-NMR (100 MHz, $CDCl_3$): 149.1, 146.5, 139.9, 131.0, 124.9, 115.8, 112.5, 111.6, 41.4, 40.3, 37.5, 25.6, 23.0, 22.6, 17.5, 10.4. IR (CCl_4): 2965, 2924, 2868, 1636, 1511, 1452, 1376, 1149, 1069, 1002. MS (CI+, NH_3): m/z 233 (MH^+), 216, 183. HRMS (EI+): m/z calcd for $C_{16}H_{24}O$: 232.1827, found: 232.1835.

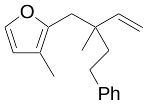

3-Methyl-2-(2-methyl-2-phenethylbut-3-enyl)furan (7e): Isolated yield: 66%. Yellow oil. ^{1}H-NMR (400 MHz, $CDCl_3$): 7.28-7.23 (m, 3H), 7.18-7.14 (m, 3H), 6.16 (d, J = 1.6 Hz, 1H), 5.87 (dd, J = 10.8, 17.5 Hz, 1H), 5.05 (dd, J = 1.2, 10.8 Hz, 1H), 4.98 (dd, J = 1.2, 17.5 Hz, 1H), 2.62 (d, J = 3.6 Hz, 2H), 2.57 (m, 2H), 1.95 (s, 3H), 1.64 (m, 2H), 1.09 (s, 3H). ^{13}C-NMR (100 MHz, $CDCl_3$): 148.9, 146.3, 143.1, 140.0, 128.3, 128.2, 125.5, 116.0, 112.6, 112.0, 42.3, 41.6, 37.3, 30.8, 22.9, 10.5. IR (CCl_4): 3082, 3027, 2930, 2866, 1636, 1602, 1502, 1455, 1374, 1204, 1149, 1071, 1003. MS (EI+): m/z 254 (M^+), 159, 128, 117, 108. HRMS (EI+): m/z calcd for $C_{18}H_{22}O$: 254.1671, found: 254.1664.

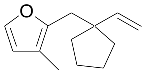

3-Methyl-2-(1-vinylcyclopentylmethyl)furan (7f): Isolated yield: 71%. Yellow oil. ^{1}H-NMR (400 MHz, $CDCl_3$): 7.23 (d, J = 1.8 Hz, 1H), 6.17 (d, J = 1.7 Hz, 1H), 5.86 (dd, J = 10.8, 17.4 Hz, 1H), 4.95 (dd, J = 1.3, 10.8 Hz, 1H), 4.93 (dd, J = 1.3, 17.4 Hz, 1H), 2.65 (s, 2H), 1.96 (s, 3H), 1.68-1.54 (m, 8H). ^{13}C-NMR (100 MHz, $CDCl_3$): 149.8, 145.8, 139.8, 115.4, 112.5, 111.0, 50.3, 36.2, 36.1, 23.5, 10.3. IR (CCl_4): 3080, 2953, 2868, 1635, 1511, 1454, 1377, 1217, 1149, 1073. MS (EI+): m/z 190 (M^+), 121, 109. HRMS (EI+): m/z calcd for $C_{13}H_{18}O$: 190.1358, found: 190.1354.

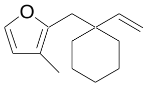

3-Methyl-2-(1-vinylcyclohexylmethyl)furan (7g): Isolated yield: 63%. Yellow oil. ^{1}H-NMR (400 MHz, $CDCl_3$): 7.23 (d, J = 1.8 Hz, 1H), 6.17 (d, J = 1.7 Hz, 1H), 5.69 (dd, J = 11.0, 17.8 Hz, 1H), 5.08 (dd, J = 1.3, 11.0 Hz, 1H), 4.94 (dd, J = 1.3, 17.8 Hz, 1H), 2.54 (s, 2H), 1.95 (s, 3H), 1.68-1.20 (m, 10H). ^{13}C-NMR (100 MHz, $CDCl_3$): 149.2, 145.9, 139.8, 115.8, 112.8, 112.5, 41.5, 38.7, 35.2, 26.3, 22.3, 10.5. IR (CCl_4): 3080, 2924, 2857, 1634, 1563, 1511, 1449, 1211, 1149, 1094, 1071, 1000. MS (EI+): m/z 204 (M^+), 121, 109. HRMS (EI+): m/z calcd for $C_{14}H_{20}O$: 204.1514, found: 204.1507.

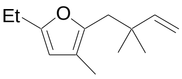

2-(2,2-Dimethylbut-3-enyl)-5-ethyl-3-methylfuran (7h): Isolated yield: quant. Pale yellow oil. ^{1}H-NMR (400 MHz, $CDCl_3$): 5.91 (dd, J = 10.7, 17.5 Hz, 1H), 5.77 (s, 1H), 4.94 (dd, J = 1.4, 17.5 Hz, 1H), 4.91 (dd, J = 1.4, 10.7 Hz, 1H), 2.57 (qd, J = 0.8, 7.5 Hz, 2H), 2.50 (s, 2H), 1.91 (s, 3H), 1.23 (t, J = 7.0 Hz, 1H), 1.04 (s, 6H). ^{13}C-NMR (100 MHz, $CDCl_3$): 155.0, 148.3, 147.2, 116.1, 109.9, 106.8, 38.7, 38.4, 26.5, 21.3, 12.2, 10.4. IR (CCl_4): 3083, 2964, 2930, 2874, 1637, 1572, 1459, 1375, 1221, 1087. MS (CI+, NH_3): m/z 193 (MH^+), 123. HRMS (EI+): m/z calcd for $C_{13}H_{20}O$: 192.1514, found: 192.1514.

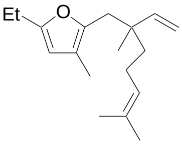

2-(2,6-Dimethyl-2-vinylhept-5-enyl)-5-ethyl-3-methylfuran (7i): Isolated yield: quant. Pale yellow oil. ^{1}H-NMR (400 MHz, $CDCl_3$): 5.82 (dd, J = 10.8, 17.5 Hz, 1H), 5.76 (s, 1H), 5.10 (thept, J = 1.3, 7.1 Hz, 1H), 5.00 (dd, J = 1.3, 10.8 Hz, 1H), 4.93 (dd, J = 1.3, 17.5 Hz, 1H), 2.57 (q, J = 7.5 Hz, 2H), 2.53 (d, J = 2.6 Hz, 2H), 1.95 (m, 2H), 1.91 (s, 3H), 1.69 (s, 3H), 1.61 (s, 3H), 1.35 (m, 2H), 1.23 (t, J = 7.0 Hz, 1H), 1.02 (s, 3H). ^{13}C-NMR (100 MHz, $CDCl_3$): 155.0, 147.0, 146.8, 130.8, 125.0, 116.2, 111.4, 106.8, 41.4, 40.2, 37.3, 25.6, 23.0, 22.7, 21.3, 17.6, 12.2, 10.5. IR (CCl_4): 3082, 2923, 1636, 1572, 1452, 1376, 1218, 1119. MS (CI+, NH_3): m/z 261 (MH^+), 123. HRMS (EI+): m/z calcd for $C_{18}H_{28}O$: 260.2140, found: 260.2132.

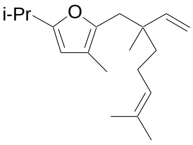

2-(2,6-Dimethyl-2-vinylhept-5-enyl)-5-isopropyl-3-methylfuran (7j): Isolated yield: 78%. Yellow oil. ^{1}H-NMR (400 MHz, $CDCl_3$): 5.82 (dd, J = 10.8, 17.5 Hz, 1H), 5.75 (s, 1H), 5.10 (thept, J = 1.3, 7.1 Hz, 1H), 4.99 (dd, J = 1.3, 10.8 Hz, 1H), 4.92 (dd, J = 1.3, 17.5 Hz, 1H), 2.85 (hept, J = 6.8 Hz, 1H), 2.53 (d, J = 2.5 Hz, 2H), 1.95 (m, 2H), 1.91 (s, 3H), 1.69 (s, 3H), 1.61 (s, 3H), 1.35 (m, 2H), 1.21 (d, J = 6.9 Hz, 6H), 1.01 (s, 3H). ^{13}C-NMR (100 MHz, $CDCl_3$): 159.0, 146.9, 146.8, 130.9, 125.0, 116.0, 111.4, 105.5, 41.4, 40.2, 37.3, 27.6, 25.6, 23.0, 22.7, 21.0, 21.0, 17.6, 10.5. IR (CCl_4): 3082, 2967, 2925, 2871, 1636, 1566, 1454, 1376, 1217, 1125, 1070, 1001. MS (CI+, NH_3): m/z 275 (MH^+), 137. HRMS (EI+): m/z calcd for $C_{19}H_{30}O$: 274.2297, found: 274.2296.

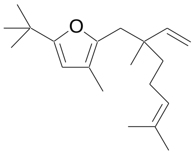

5-*tert*-Butyl-2-(2,6-dimethyl-2-vinylhept-5-enyl)-3-methylfuran (7k): Isolated yield: 78%. Yellow oil. ^{1}H-NMR (400 MHz, $CDCl_3$): 5.82 (dd, J = 10.8, 17.5 Hz, 1H), 5.73 (s, 1H), 5.09 (thept, J = 1.4, 7.1 Hz, 1H), 4.98 (dd, J = 1.4, 10.8 Hz, 1H), 4.91 (dd, J = 1.4, 17.5 Hz, 1H), 2.53 (d, J = 2.4 Hz, 2H), 1.96 (m, 2H), 1.91 (s, 3H), 1.68 (s, 3H), 1.60 (s, 3H), 1.35 (m, 2H), 1.24 (s, 9H), 1.01 (s, 3H). ^{13}C-NMR (100 MHz, $CDCl_3$): 161.4, 146.8, 146.8, 130.8, 125.1, 115.8, 111.3, 104.6, 41.4, 40.3, 37.2, 32.2, 28.9, 25.6, 23.0, 22.8, 17.6, 10.5. IR (CCl_4): 3081, 2965, 2925, 2866, 1636, 1561, 1455, 1369, 1293, 1196, 1103, 1001. MS (EI+): m/z 288 (M^+), 151, 136, 109. HRMS (EI+): m/z calcd for $C_{20}H_{32}O$: 288.2453, found: 288.2461.

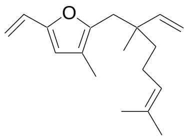

2-(2,6-Dimethyl-2-vinylhept-5-enyl)-3-methyl-5-vinylfuran (7l): Isolated yield: 17%. Yellow oil. ^{1}H-NMR (400 MHz, $CDCl_3$): 6.41 (dd, J = 11.2, 17.5 Hz, 1H), 6.05 (s, 1H), 5.83 (dd, J = 10.8, 17.5 Hz, 1H), 5.53 (dd, J = 1.4, 17.5 Hz, 1H), 5.10 (thept, J = 1.3, 7.0 Hz, 1H), 5.05 (dd, J = 1.4, 11.2 Hz, 1H), 5.00 (dd, J = 1.3, 10.8 Hz, 1H), 4.93 (dd, J = 1.3, 17.5 Hz, 1H), 2.57 (d, J = 2.4 Hz, 2H), 1.94 (m, 2H), 1.92 (s, 3H), 1.68 (s, 3H), 1.60 (s, 3H), 1.38 (m, 2H), 1.04 (s, 3H). ^{13}C-NMR (100 MHz, $CDCl_3$): 150.6, 149.1, 146.5, 131.0, 125.1, 124.9, 117.7, 111.6, 111.3, 110.4, 41.2, 40.2, 37.3, 25.6, 23.0, 22.8, 17.6, 10.4. IR (CCl_4): 3083, 2964, 2924, 2860, 1639, 1606, 1531, 1451, 1375, 1297, 1122, 1028, 1001. MS (EI+): m/z 258 (M^+), 205, 149, 135, 121. HRMS (EI+): m/z calcd for $C_{18}H_{26}O$: 258.1984, found: 258.1982.

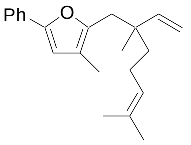

2-(2,6-Dimethyl-2-vinylhept-5-enyl)-3-methyl-5-phenylfuran (7m): Isolated yield: 77%. Yellow oil. ^{1}H-NMR (400 MHz, $CDCl_3$): 7.63 (dd, J = 1.2, 8.2 Hz, 2H), 7.37 (t, J = 8.0 Hz, 2H), 7.23 (tt, J = 1.2, 7.4 Hz, 1H), 6.48 (s, 1H), 5.89 (dd, J = 10.8, 17.5 Hz, 1H), 5.14 (thept, J = 1.4, 7.0 Hz, 1H), 5.06 (dd, J = 1.3, 10.8 Hz, 1H), 4.98 (dd, J = 1.3, 17.5 Hz, 1H), 2.66 (d, J = 2.4 Hz, 2H), 2.03 (m, 2H), 2.01 (s, 3H), 1.72 (s, 3H), 1.64 (s, 3H), 1.45 (m, 2H), 1.24 (s, 9H), 1.11 (s, 3H). ^{13}C-NMR (100 MHz, $CDCl_3$): 151.0, 149.1, 146.5, 131.2, 131.0, 128.5, 126.5, 124.9, 123.2, 118.2, 111.7, 108.1, 41.3, 40.3, 37.3, 25.6, 23.0, 22.9, 17.6, 10.5. IR (CCl_4): 3079, 3031, 2967, 2923, 2866, 1636, 1600, 1550, 1484, 1448, 1376, 1182, 1121, 1068, 1001. MS (CI+, NH_3): m/z 309 (MH^+), 171. HRMS (EI+): m/z calcd for $C_{22}H_{28}O$: 308.214, found: 308.2147.

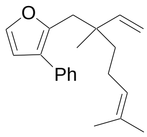

2-(2,6-Dimethyl-2-vinylhept-5-enyl)-3-phenylfuran (7n): Isolated yield: 80%. Yellow oil. ^{1}H-NMR (400 MHz, $CDCl_3$): 7.43-7.36 (m, 5H), 7.28 (m, 1H), 6.48 (d, J = 1.8 Hz, 1H), 5.77 (dd, J = 10.8, 17.5 Hz, 1H), 5.03 (thept, J = 1.4, 7.2 Hz, 1H), 4.95 (dd, J = 1.3, 10.8 Hz, 1H), 4.90 (dd, J = 1.3, 17.5 Hz, 1H), 2.85 (s, 2H), 1.87 (q, J = 8.2 Hz, 2H), 1.68 (s, 3H), 1.57 (s, 3H), 1.35 (m, 2H), 0.99 (s, 3H). ^{13}C-NMR (100 MHz, $CDCl_3$): 149.6, 146.4, 140.5, 134.6, 131.0, 128.4, 128.2, 126.4, 124.8, 123.2, 111.7, 111.6, 41.4, 40.5, 37.5, 25.6, 22.9, 22.9, 17.5. IR (CCl_4): 3079, 3028, 2966, 2921, 2862, 1611, 1515, 1449, 1376, 1202, 1143, 1057, 1000. MS (EI+): m/z 294 (M^+), 170, 157, 137, 129. HRMS (EI+): m/z calcd for $C_{21}H_{26}O$: 294.1984, found: 294.1988.

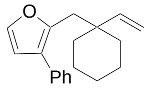

3-Phenyl-2-(1-vinylcyclohexylmethyl)furan (7o): Isolated yield: 90%. Yellow oil. ^{1}H-NMR (400 MHz, $CDCl_3$): 7.40-7.32 (m, 5H), 7.25 (m, 1H), 6.44 (d, J = 1.9 Hz, 1H), 5.62 (dd, J = 11.0, 17.8 Hz, 1H), 4.97 (dd, J = 1.3, 10.9 Hz, 1H), 4.86 (dd, J = 1.3, 17.8 Hz, 1H), 2.79 (s, 2H), 1.58 (m, 2H), 1.47-1.32 (m, 8H). ^{13}C-NMR (100 MHz, $CDCl_3$): 149.6, 145.7, 140.4, 134.7, 128.3, 128.2, 126.3, 123.1, 112.9, 111.5, 41.5, 38.6, 35.5, 26.2, 22.2. IR (CCl_4): 3078, 3031, 2937, 2856, 1611, 1515, 1449, 1213, 1143, 1057. MS (EI+): m/z 266 (M^+). HRMS (EI+): m/z calcd for $C_{19}H_{22}O$: 266.1671, found: 266.1671.

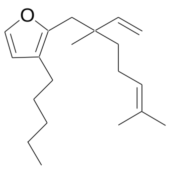

2-(2,6-Dimethyl-2-vinylhept-5-enyl)-3-pentylfuran (7p): Isolated yield: 82%. Yellow oil. ^{1}H-NMR (400 MHz, $CDCl_3$): 7.25 (d, J = 1.8 Hz, 1H), 6.22 (d, J = 1.8 Hz, 1H), 5.82 (dd, J = 10.8, 17.5 Hz, 1H), 5.11 (thept, J = 1.3, 7.1 Hz, 1H), 5.01 (dd, J = 1.3, 10.8 Hz, 1H), 4.93 (dd, J = 1.3, 17.5 Hz, 1H), 2.58 (s, 2H), 2.31 (t, J = 7.6 Hz, 2H), 1.94 (q, J = 7.5 Hz, 2H), 1.69 (s, 3H), 1.61 (s, 3H), 1.53 (m, 2H), 1.40-1.28 (m, 6H), 1.03 (s, 3H), 0.92 (t, J = 7.0 Hz, 3H). ^{13}C-NMR (100 MHz, $CDCl_3$): 148.6, 146.6, 140.0, 130.9, 124.9, 121.2, 111.6, 111.0, 41.2, 40.3, 37.6, 31.7, 30.0, 25.6, 25.0, 23.0, 22.6, 22.5, 17.5, 14.0. IR (CCl_4): 3081, 2923, 2860, 1637, 1511, 1456, 1376, 1188, 1147, 1051, 1002. MS (EI+): m/z 288 (M^+), 245, 232, 219, 205. HRMS (EI+): m/z calcd for $C_{20}H_{32}O$: 288.2453, found: 288.2451.

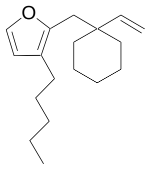

3-Pentyl-2-(1-vinylcyclohexylmethyl)furan (7q): Isolated yield: 86%. Yellow oil. ^{1}H-NMR (400 MHz, $CDCl_3$): 7.24 (d, J = 1.8 Hz, 1H), 6.21 (d, J = 1.8 Hz, 1H), 5.68 (dd, J = 11.0, 17.8 Hz, 1H), 5.07 (dd, J = 1.3, 11.0 Hz, 1H), 4.94 (dd, J = 1.4, 17.8 Hz, 1H), 2.55 (s, 2H), 2.30 (t, J = 7.6 Hz, 2H), 1.63 (m, 2H), 1.57-1.26 (m, 14H), 0.92 (t, J = 7.0 Hz, 3H). ^{13}C-NMR (100 MHz, $CDCl_3$): 148.6, 145.9, 139.9, 121.2, 112.8, 110.9, 41.3, 38.7, 35.3, 31.7, 30.0, 26.3, 25.0, 22.5, 22.3, 14.0. IR (CCl_4): 3080, 2934, 2859, 1635, 1511, 1452, 1377, 1149, 1114, 1050, 1001. MS (EI+): m/z 260 (M^+), 220, 205. HRMS (EI+): m/z calcd for $C_{18}H_{28}O$: 260.2140, found: 260.2140.

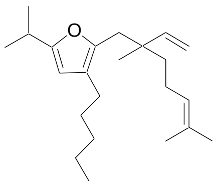

2-(2,6-Dimethyl-2-vinylhept-5-enyl)-5-isopropyl-3-pentylfuran (7r): Isolated yield: 73%. Yellow oil. ^{1}H-NMR (400 MHz, $CDCl_3$): 5.82 (dd, J = 10.8, 17.5 Hz, 1H), 5.79 (s, 1H), 5.10 (thept, J = 1.2, 7.1 Hz, 1H), 4.99 (dd, J = 1.3, 10.8 Hz, 1H), 4.92 (dd, J = 1.3, 17.5 Hz, 1H), 2.85 (hept, J = 6.8 Hz, 1H), 2.53 (d, J = 2.0 Hz, 2H), 2.26 (t, J = 7.7 Hz, 2H), 1.95 (m, 2H), 1.69 (s, 3H), 1.61 (s, 3H), 1.51 (m, 2H), 1.40-1.28 (m, 6H), 1.21 (d, J = 6.9 Hz, 6H), 1.02 (s, 3H), 0.92 (t, J = 7.0 Hz, 3H). ^{13}C-NMR (100 MHz, $CDCl_3$): 159.1, 146.9, 146.3, 130.8, 125.0, 121.4, 111.3, 103.9, 41.1, 40.3, 37.3, 31.8, 30.1, 27.7, 25.6, 25.2, 23.0, 22.8, 22.5, 21.0, 17.6, 14.0. IR (CCl_4): 3081, 2931, 2862, 1636, 1565, 1457, 1376, 1337, 1216, 1125, 1060, 1001. MS (EI+): m/z 330 (M^+), 292, 274, 242, 230, 218, 205. HRMS (EI+): m/z calcd for $C_{23}H_{38}O$: 330.2923, found: 330.2922.

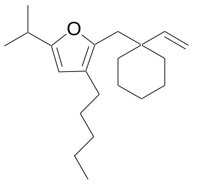

5-Isopropyl-3-pentyl-2-(1-vinylcyclohexylmethyl)furan (7s): Isolated yield: 36%. Yellow oil. ^{1}H-NMR (400 MHz, $CDCl_3$): 5.77 (s, 1H), 5.68 (dd, J = 10.9, 17.8 Hz, 1H), 5.04 (dd, J = 1.3, 11.0 Hz, 1H), 4.91 (dd, J = 1.4, 17.8 Hz, 1H), 2.85 (hept, J = 6.8 Hz, 1H), 2.49 (s, 2H), 2.24 (t, J = 7.7 Hz, 2H), 1.64-1.22 (m, 16H), 1.21 (d, J = 6.9 Hz, 6H), 0.91 (t, J = 7.0 Hz, 3H). ^{13}C-NMR (100 MHz, $CDCl_3$): 158.9, 146.3, 146.3, 121.3, 112.5, 103.8, 41.2, 38.6, 35.2, 31.8, 30.1, 27.7, 26.4, 25.2, 22.5, 22.3, 21.0, 14.0. IR (CCl_4): 3080, 2928, 2858, 1635, 1566, 1454, 1378, 1218, 1118, 1065, 1000. MS (EI+): m/z 302 (M^+), 206. HRMS (EI+): m/z calcd for $C_{21}H_{34}O$: 302.2610, found: 302.2612.
