# Supplementary material for: Gold(I)-catalyzed formation of furans by a Claisen-type rearrangement of ynenyl allyl ethers
Source: Beilstein J Org Chem. 2011 Jun 29;7:878–85. doi: 10.3762/bjoc.7.100 (PMC3135071; doi:10.3762/bjoc.7.100)
Supplement: File 2 — NMR spectral data for substrates 6a–s. [file Beilstein_J_Org_Chem-07-878-s002.pdf]

Supporting Information

for

Gold(I)-catalyzed formation of furans by a Claisen-type rearrangement of ynenyl allyl ethers

Florin M. Istrate and Fabien Gagosz*

Address: Département de Chimie, UMR 7652, CNRS/Ecole Polytechnique, 91128 Palaiseau, France

Email: Fabien Gagosz - gagosz@dcso.polytechnique.fr

* Corresponding Author

Spectra of substrates 6a-s

Spectra of compound **6a**	p S2-S3
Spectra of compound **6b**	p S4-S5
Spectra of compound **6c**	p S6-S7
Spectra of compound **6d**	p S8-S9
Spectra of compound **6e**	p S10-S11
Spectra of compound **6f**	p S12-S13
Spectra of compound **6g**	p S14-S15
Spectra of compound **6h**	p S16-S17
Spectra of compound **6i**	p S18-S19
Spectra of compound **6j**	p S20-S21
Spectra of compound **6k**	p S22-S23
Spectra of compound **6l**	p S24-S25
Spectra of compound **6m**	p S26-S27
Spectra of compound **6n**	p S28-S29
Spectra of compound **6o**	p S30-S31
Spectra of compound **6p**	p S32-S33
Spectra of compound **6q**	p S34-S35
Spectra of compound **6r**	p S36-S37
Spectra of compound **6s**	p S38-S39

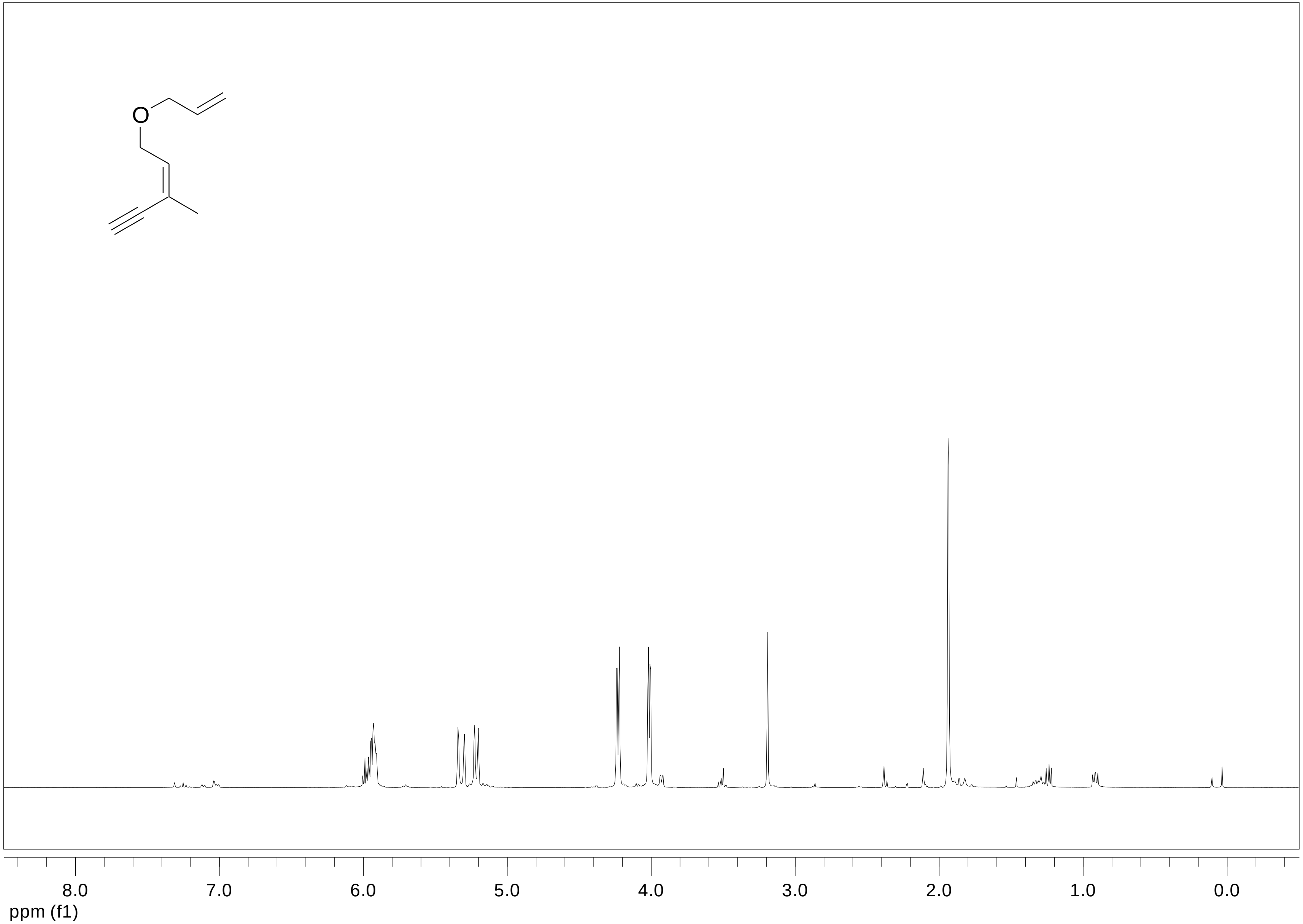

Compound 6a

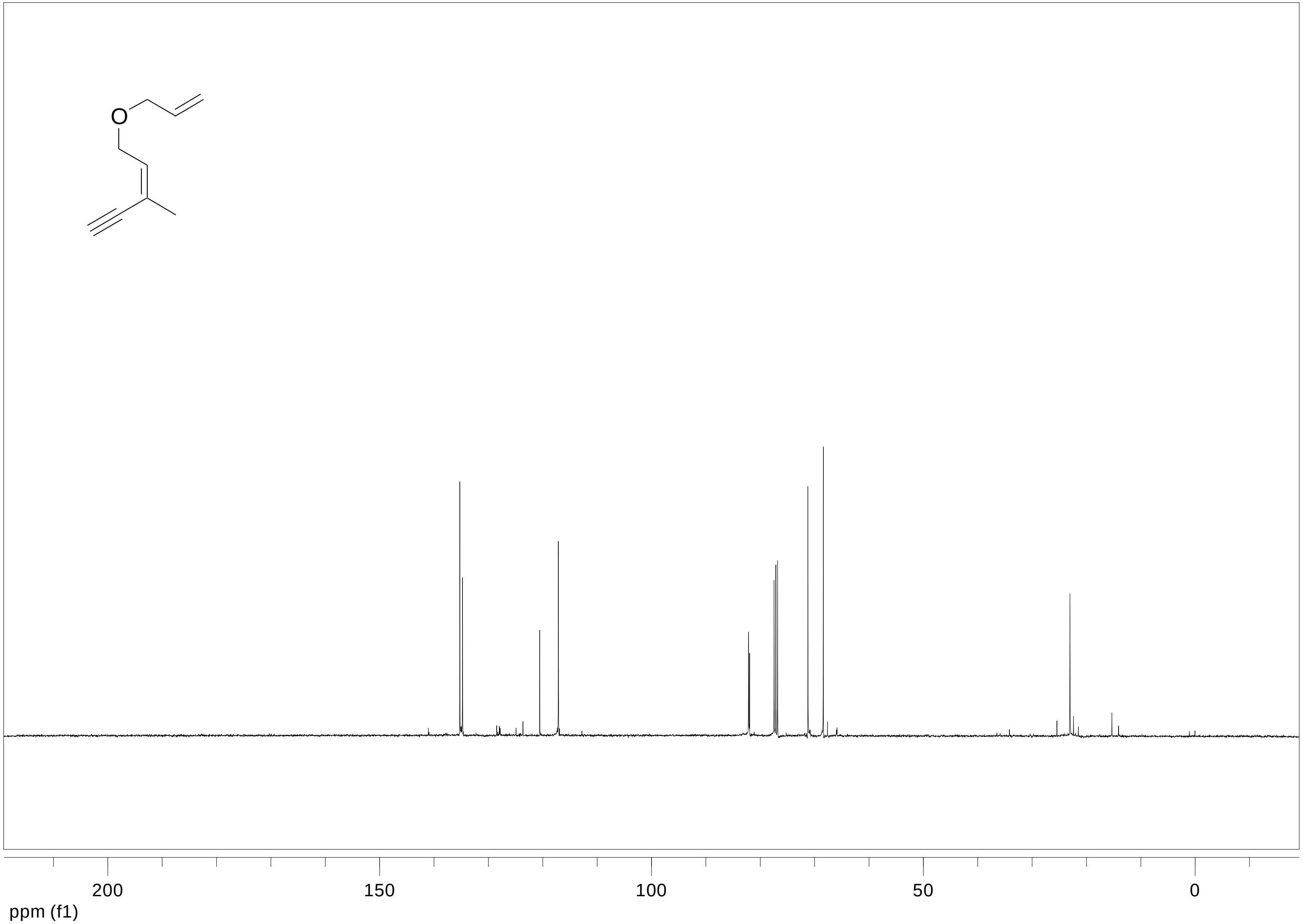

Compound 6a

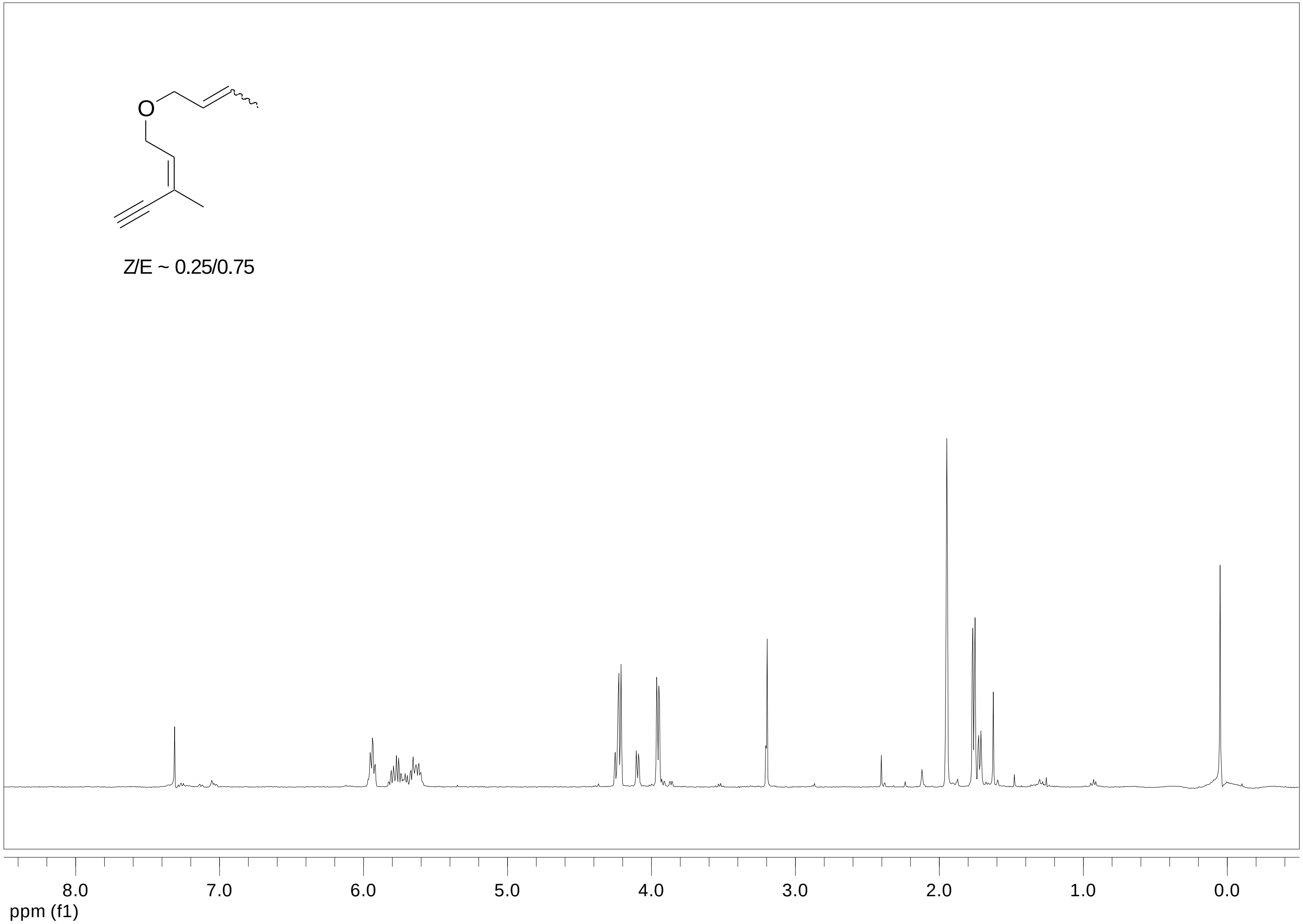

Compound 6b

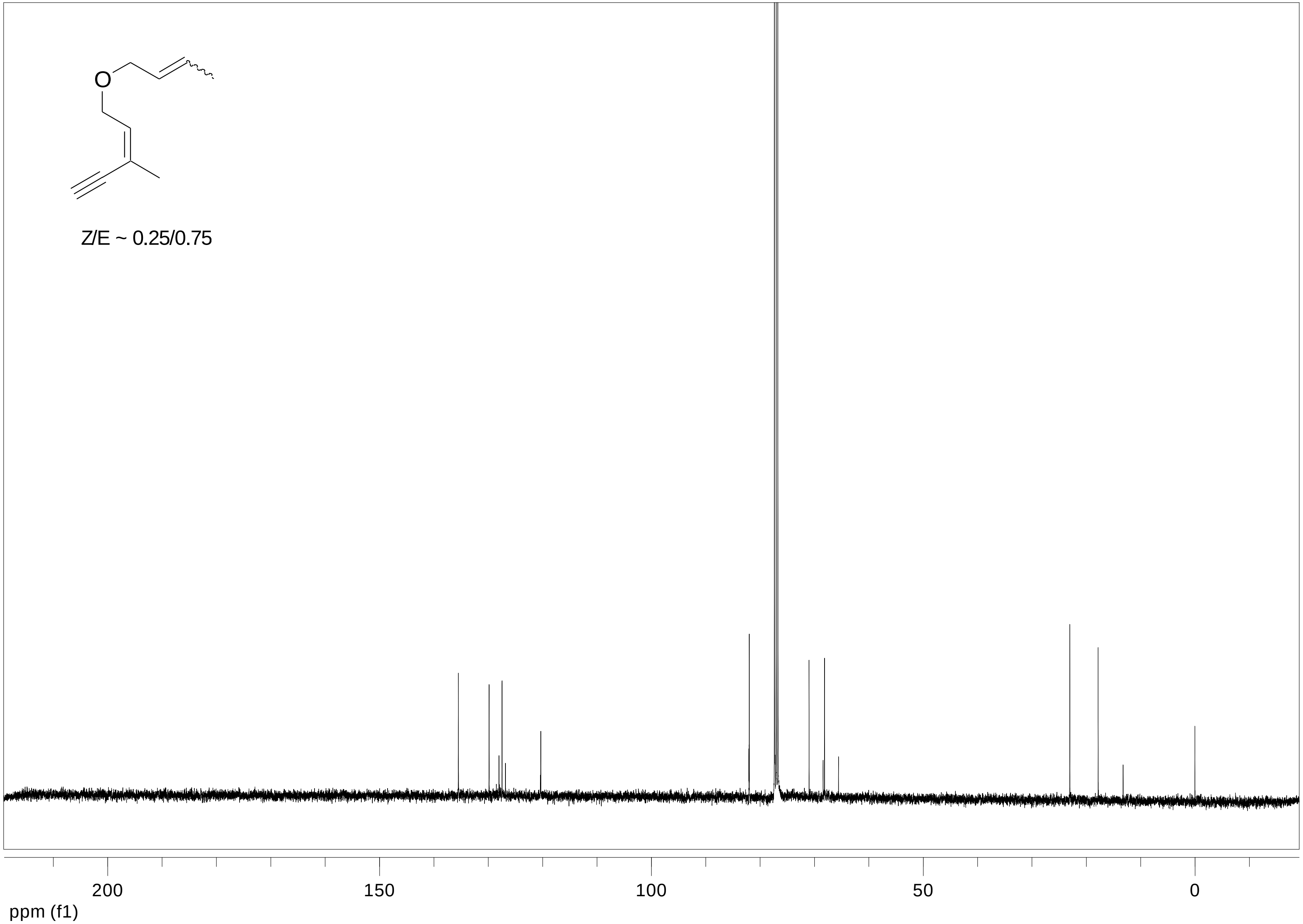

Compound 6b

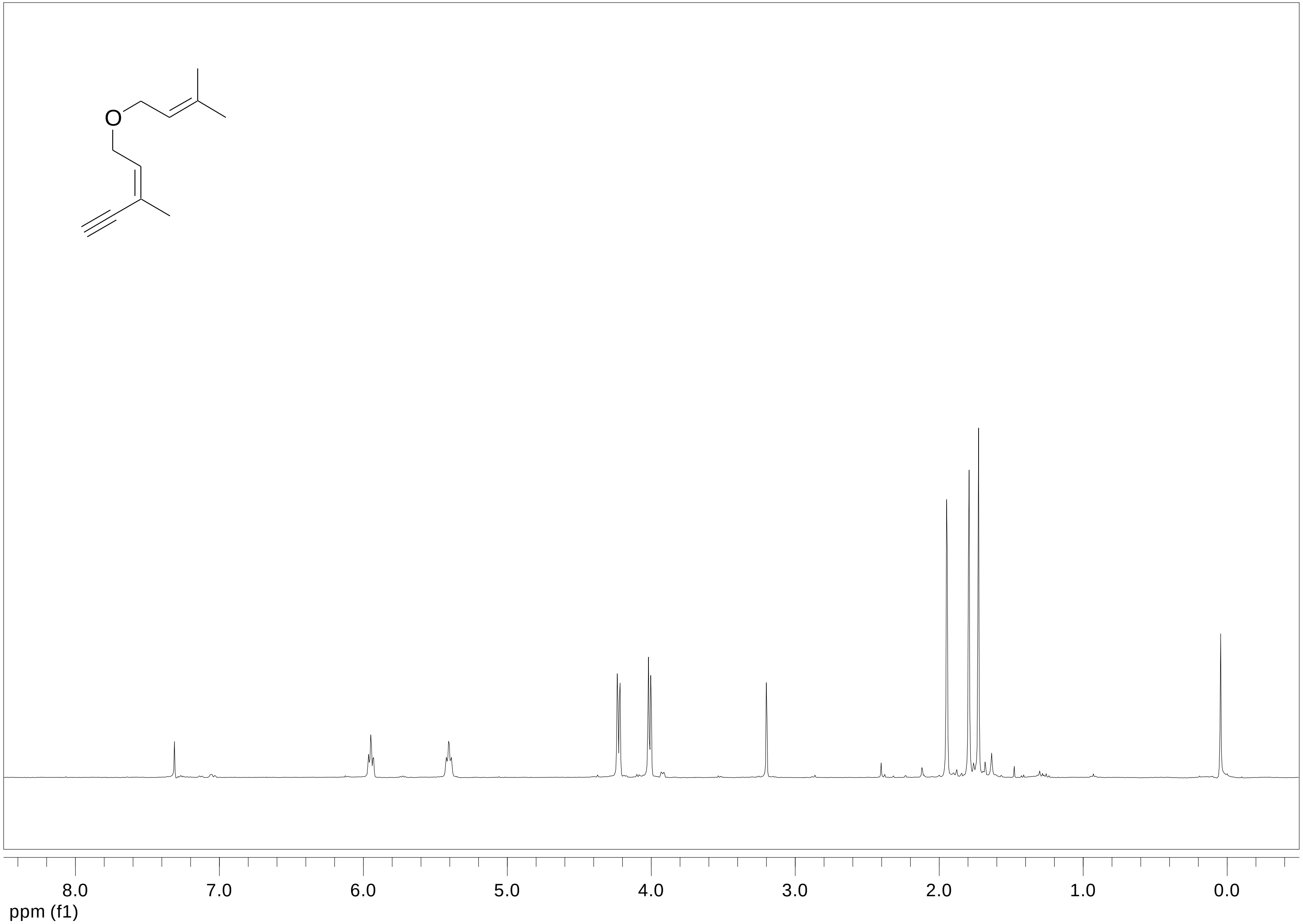

Compound 6c

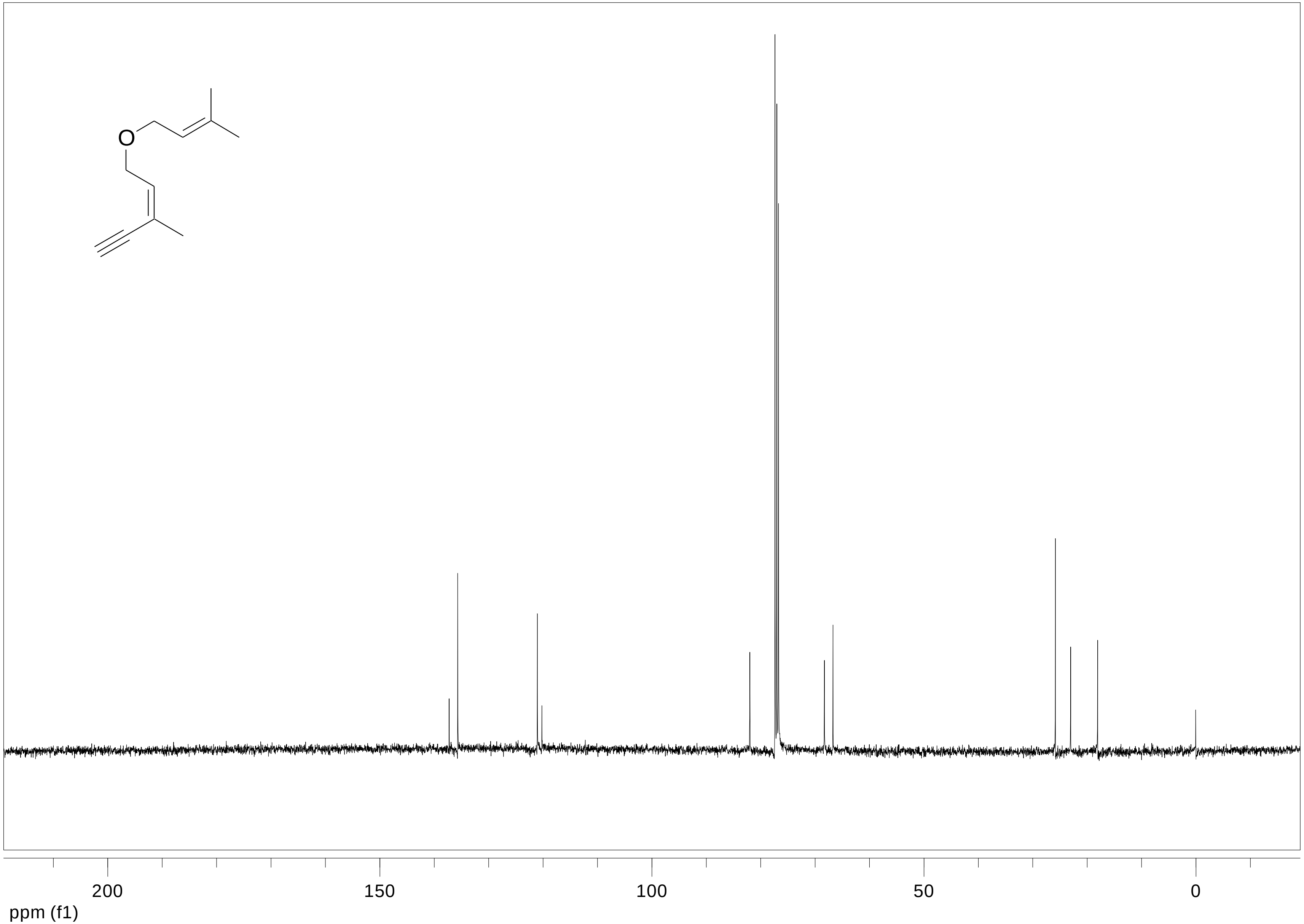

Compound 6c

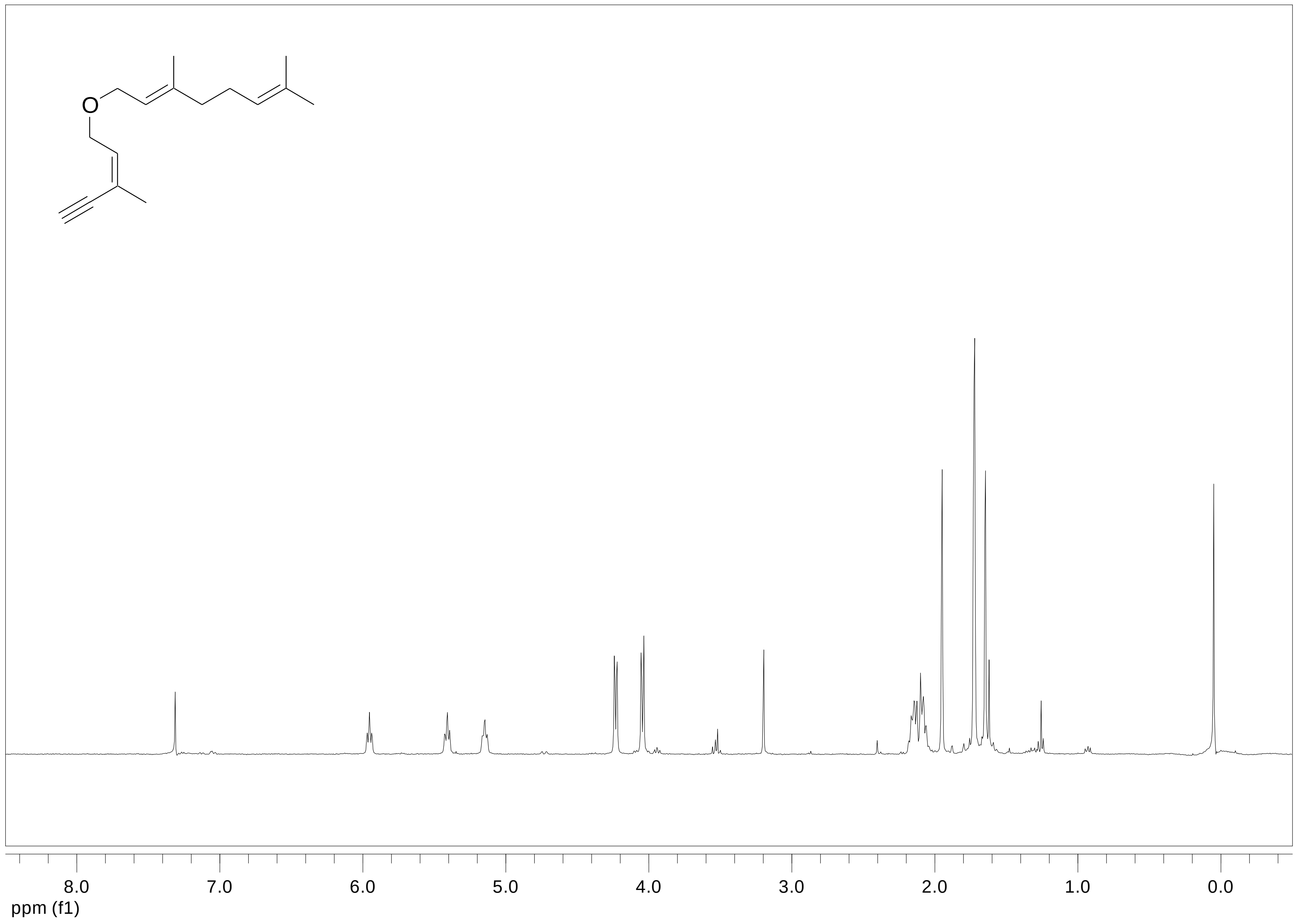

Compound 6d

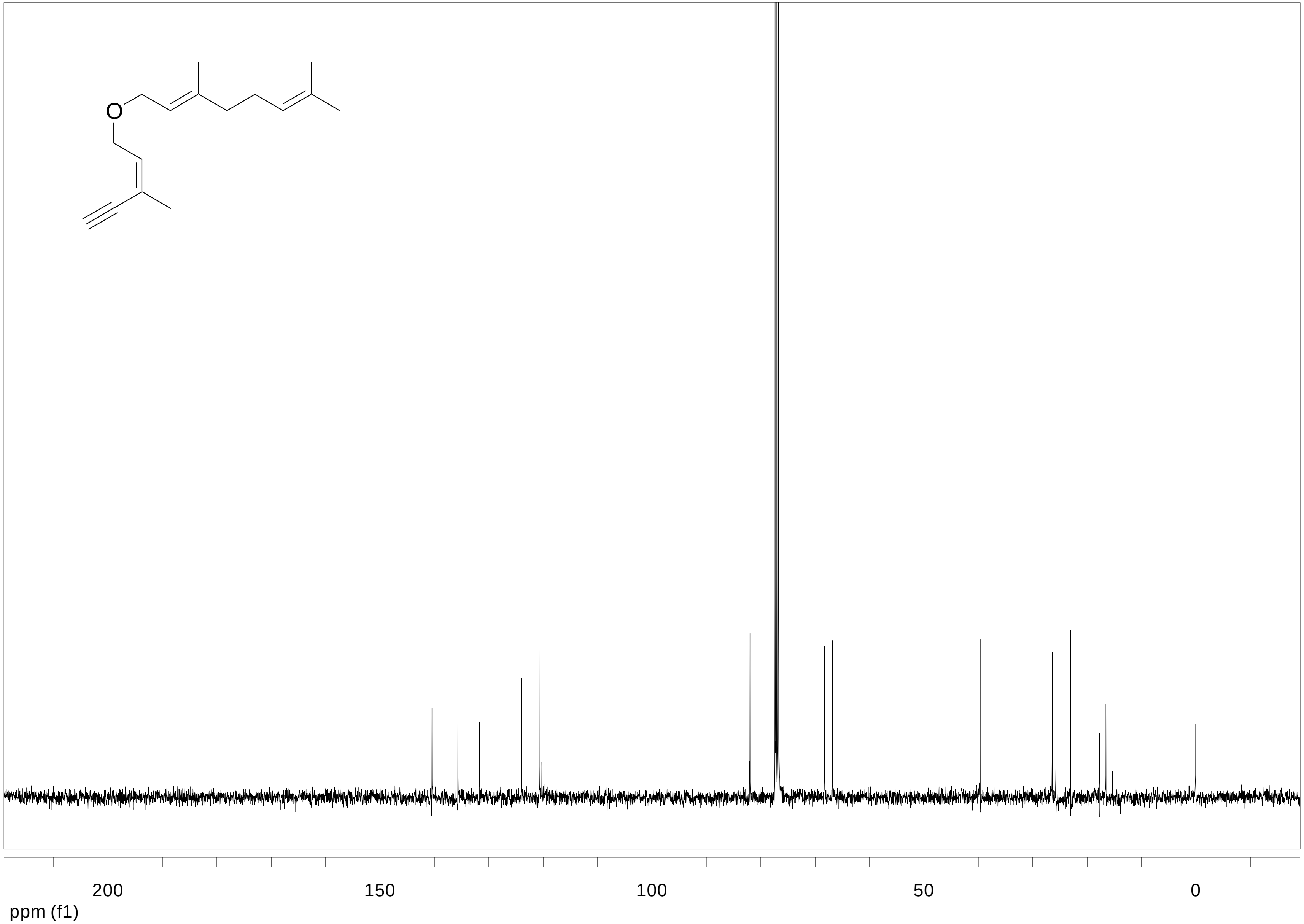

Compound 6d

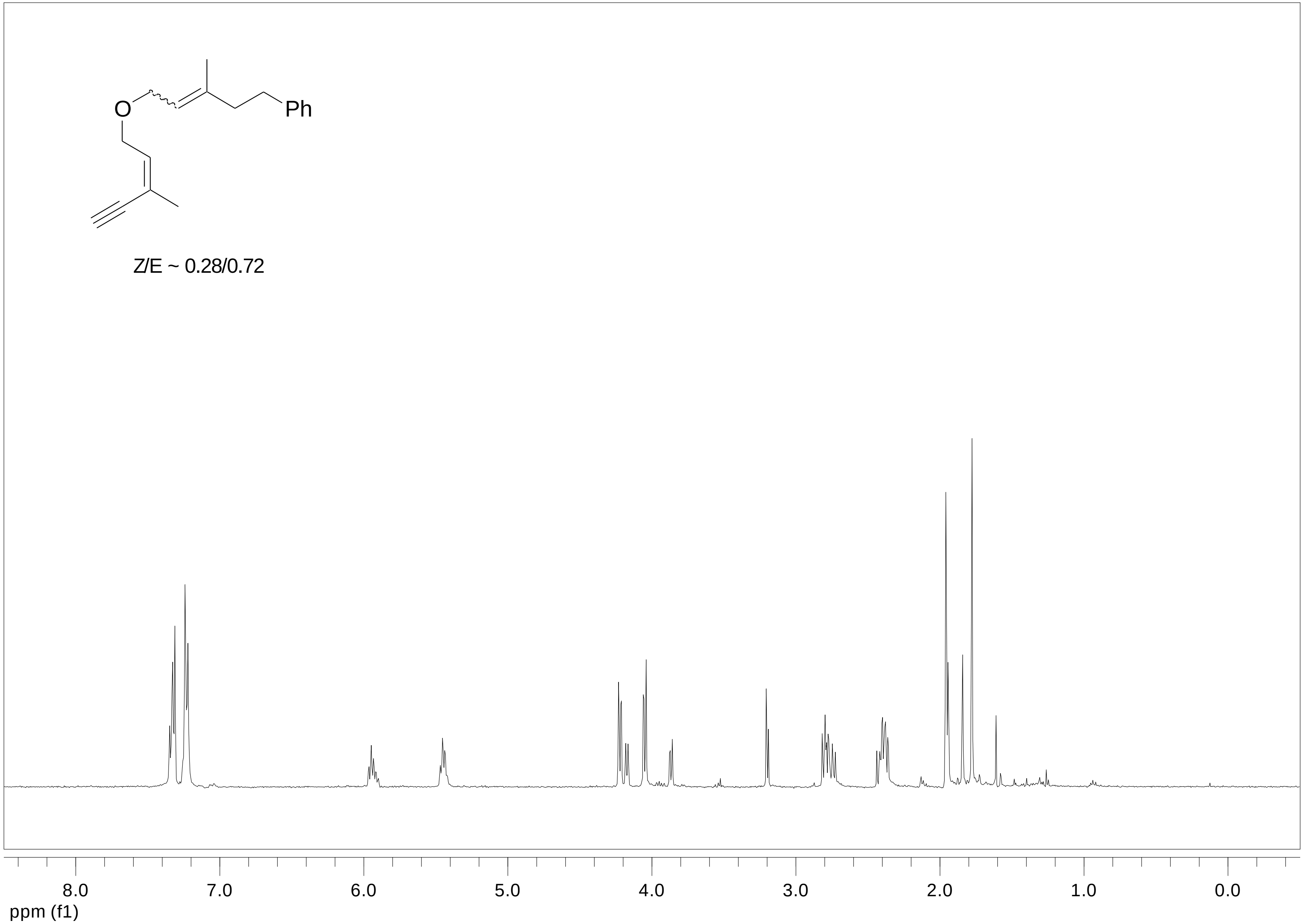

Compound 6e

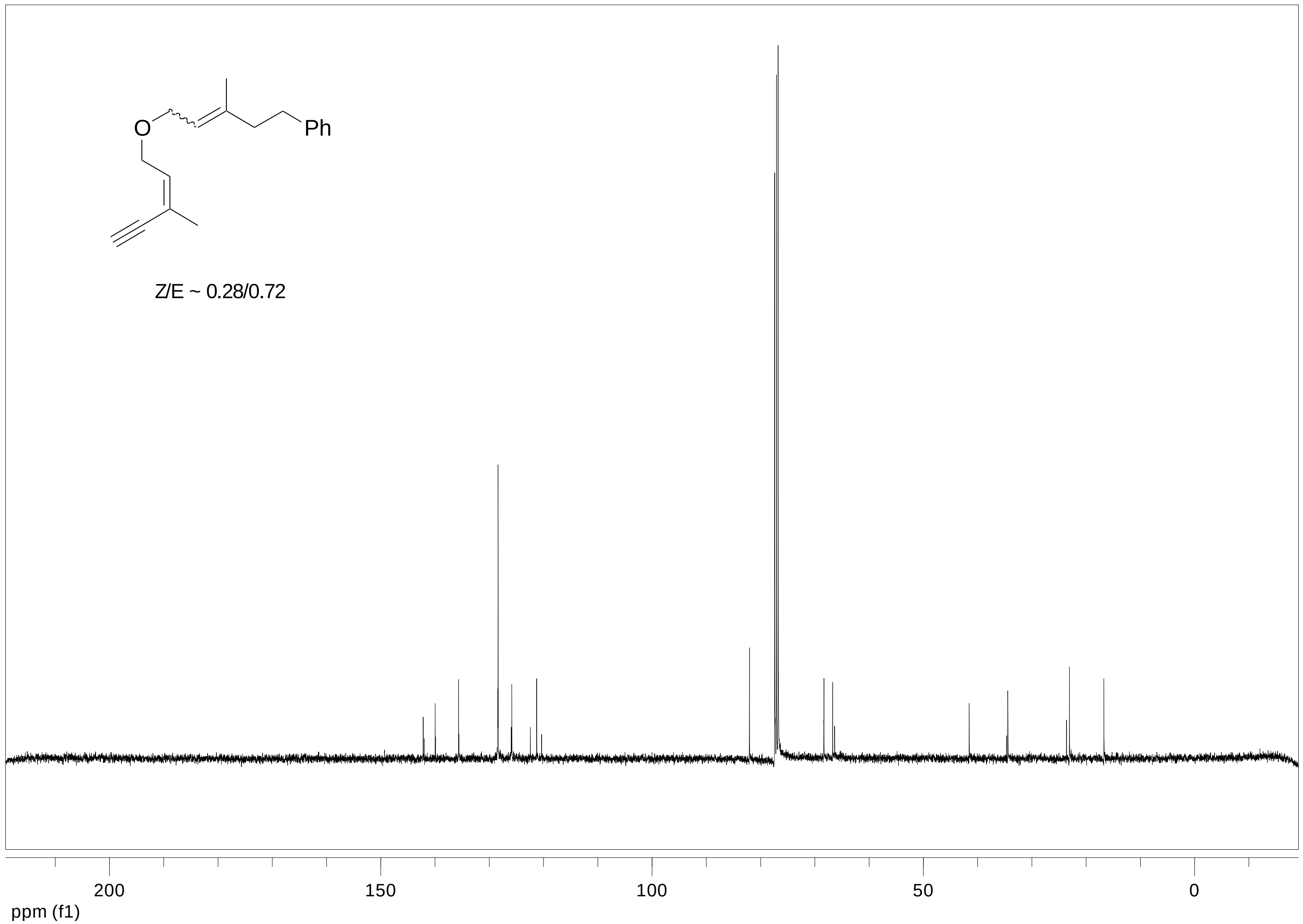

Compound 6e

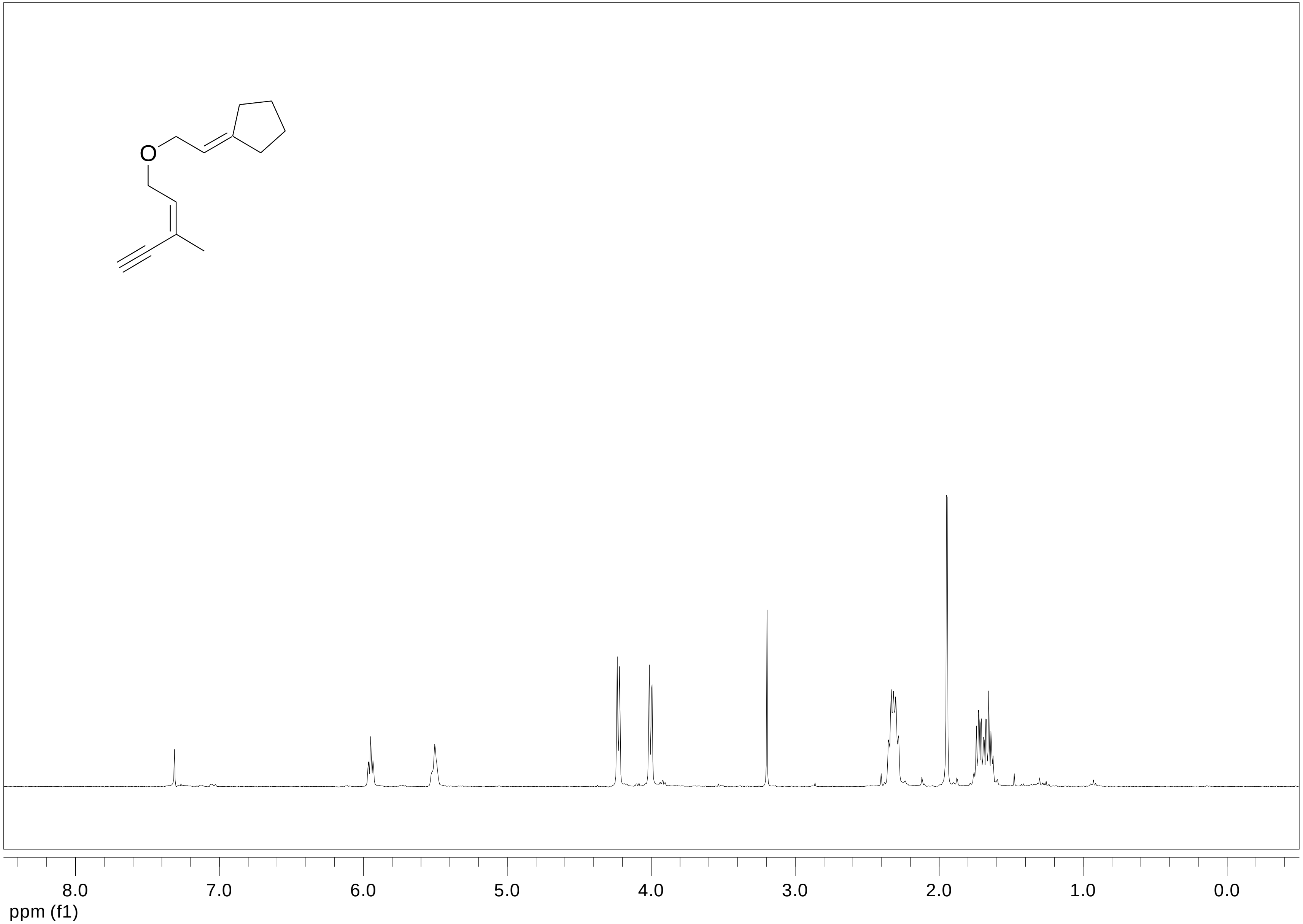

Compound 6f

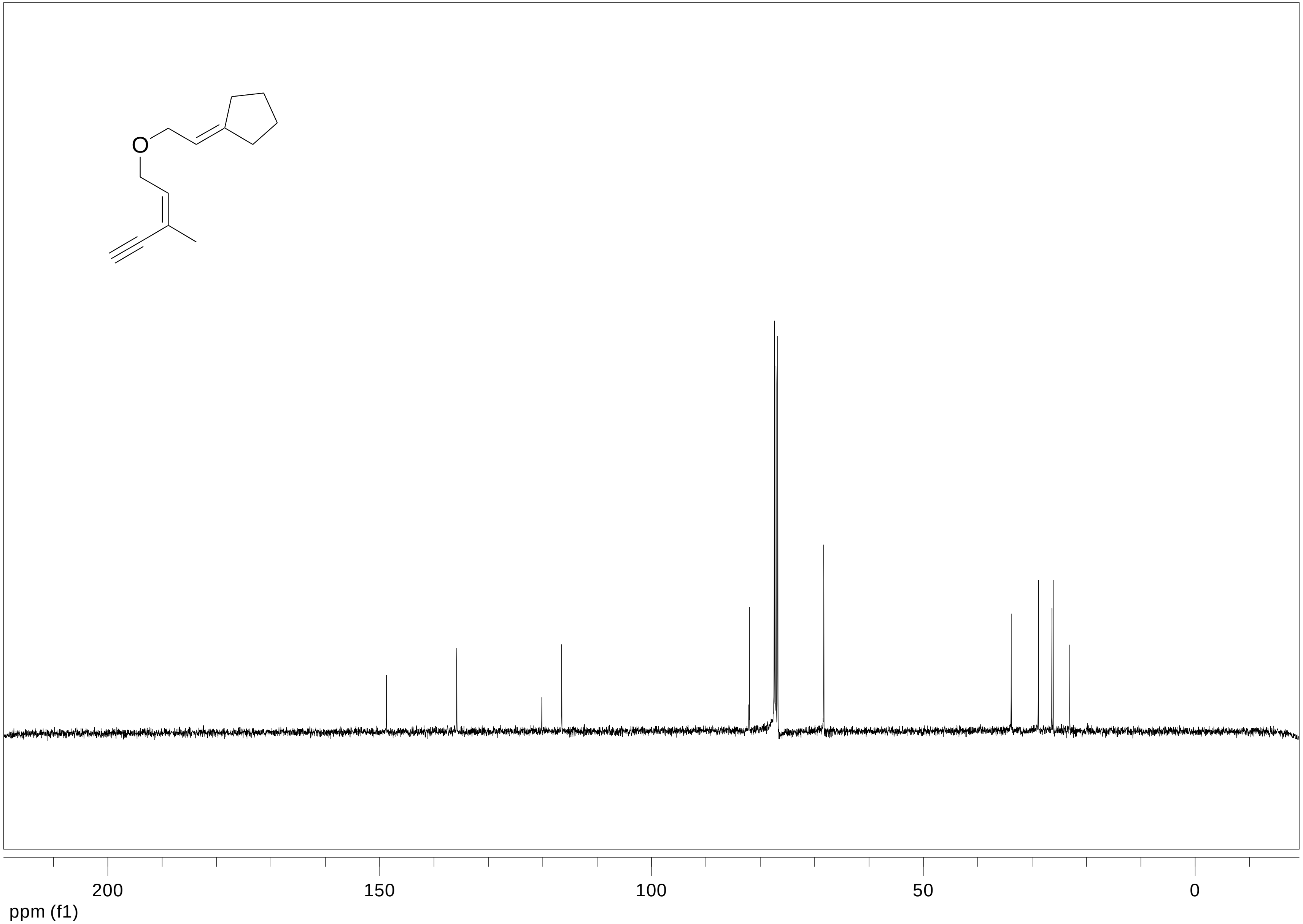

Compound 6f

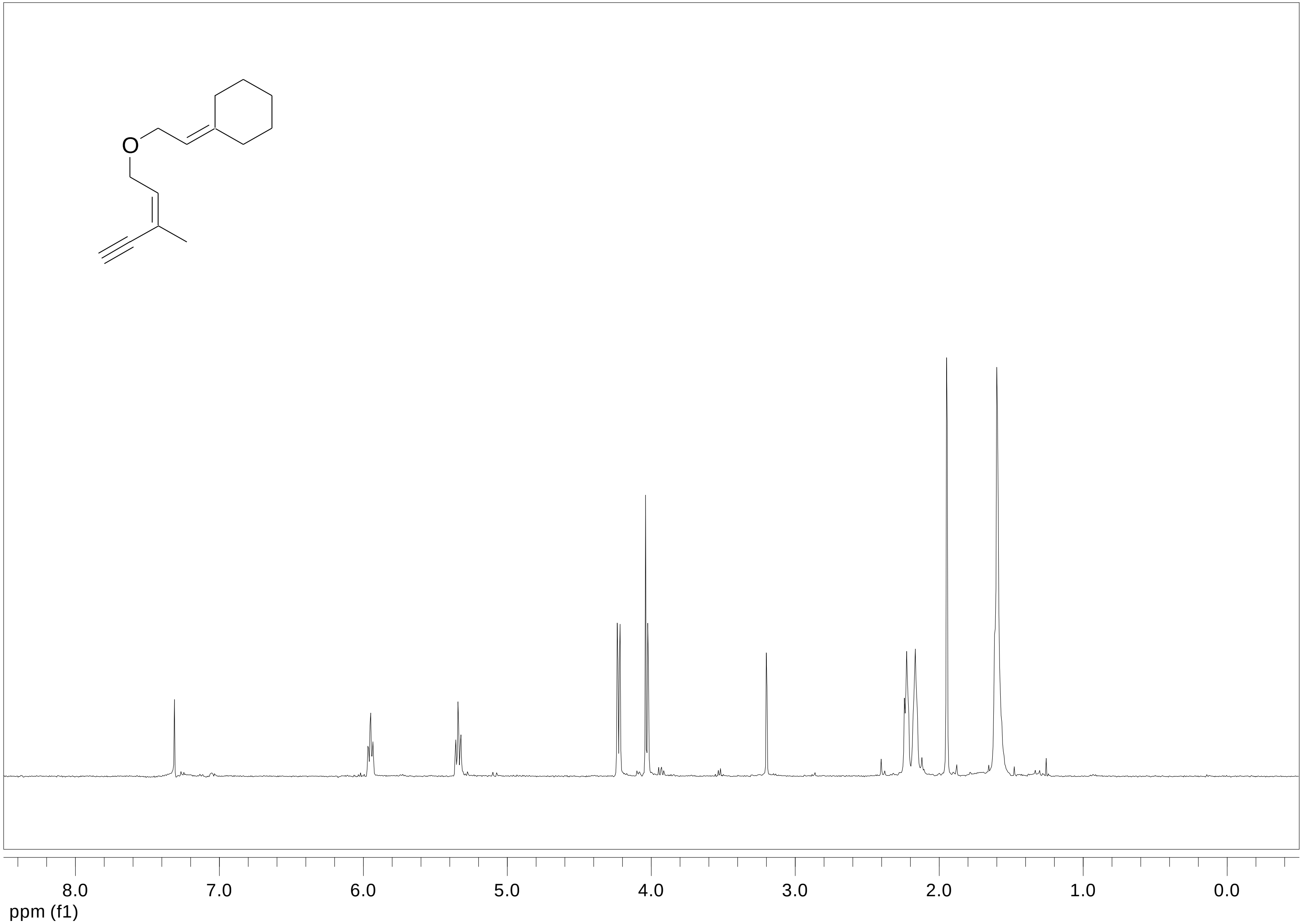

Compound 6g

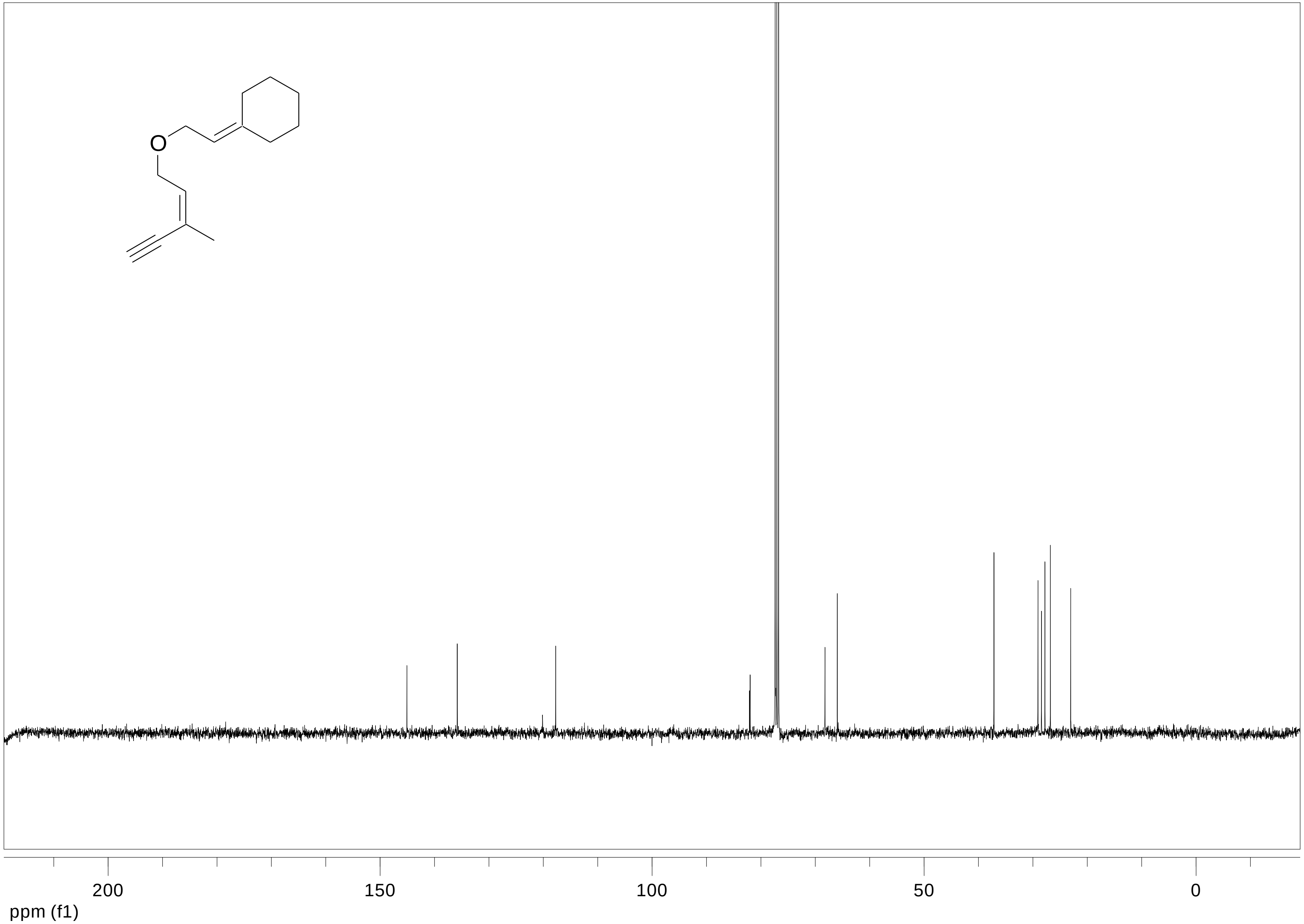

Compound 6g

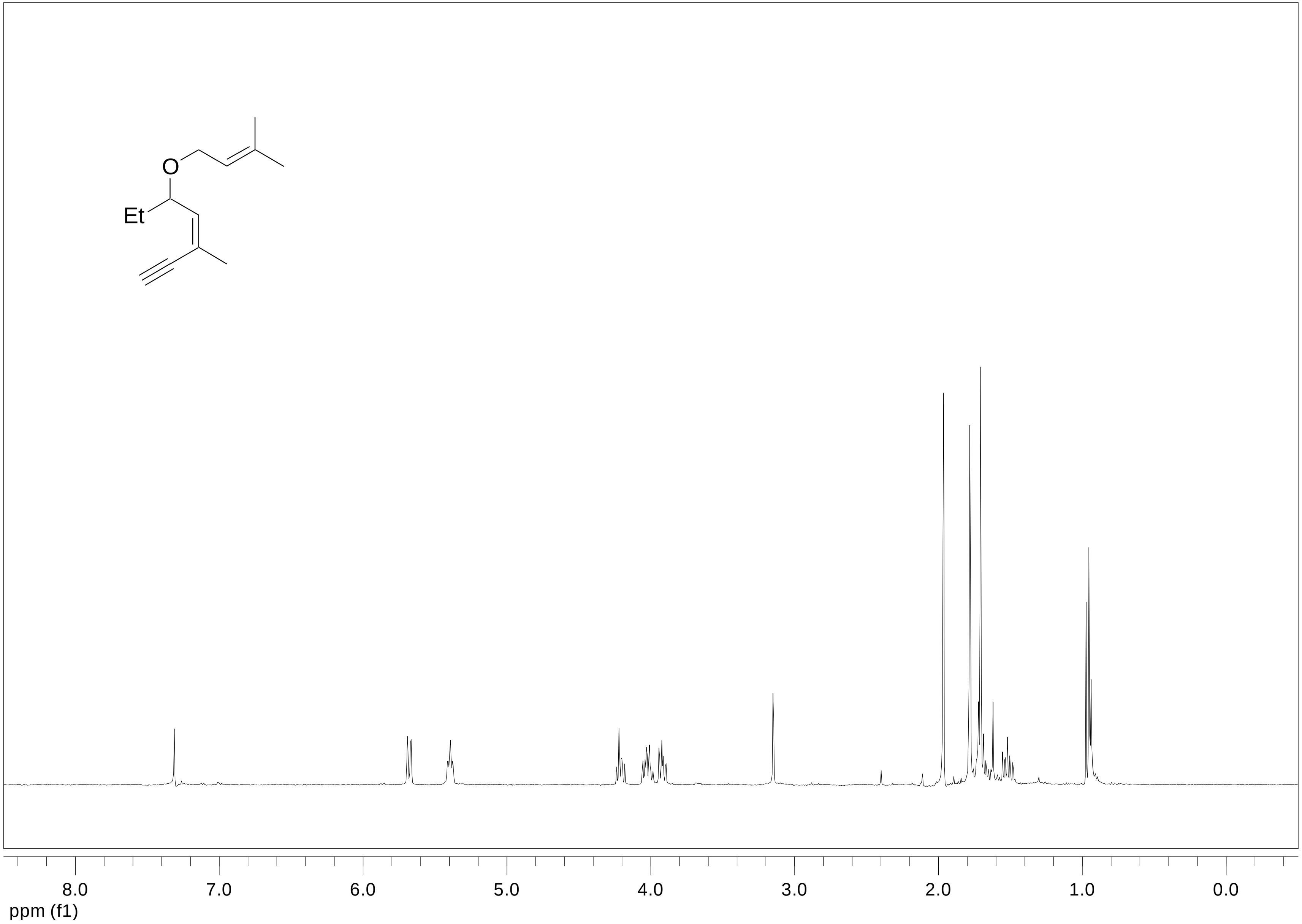

Compound 6h

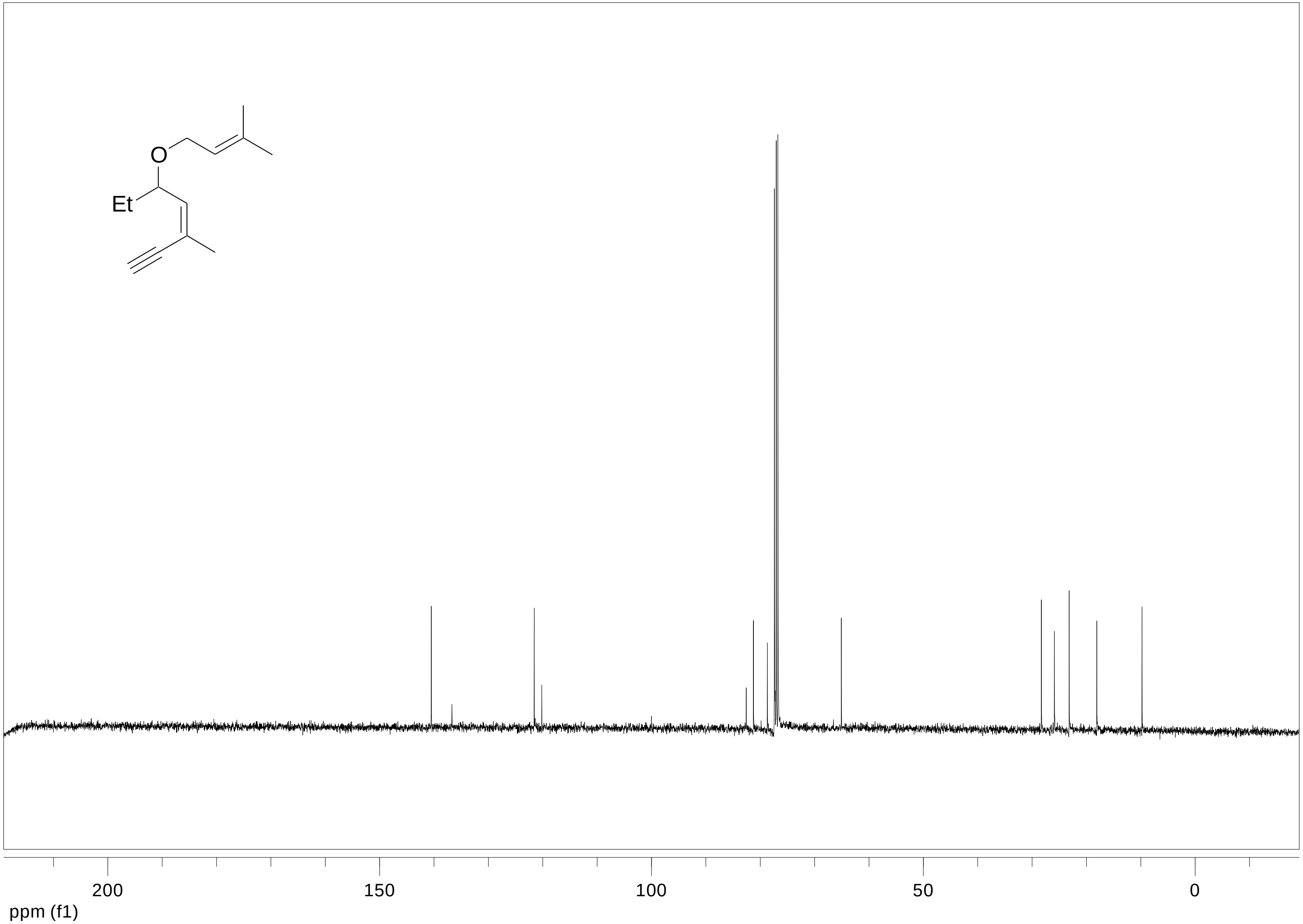

Compound 6h

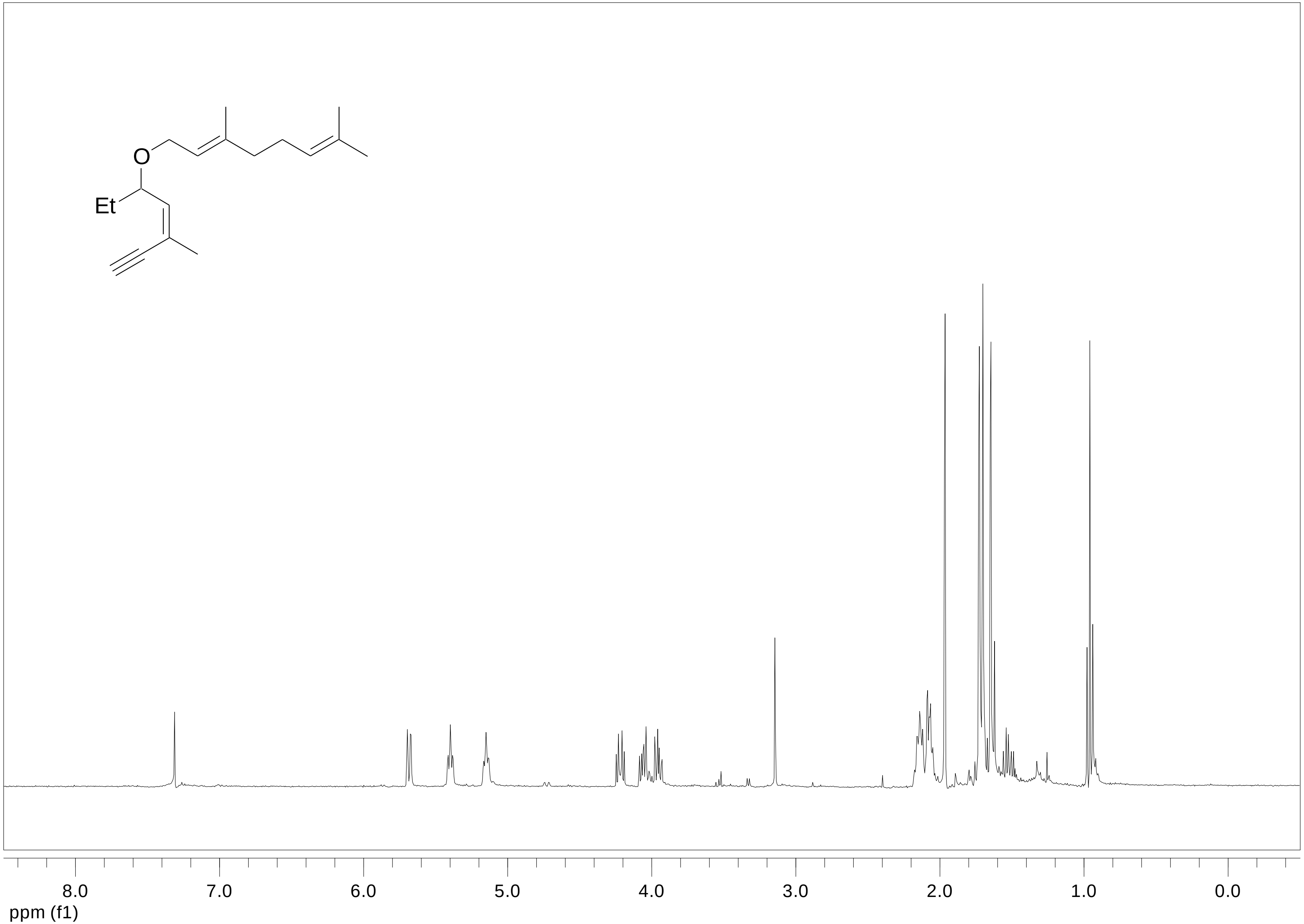

Compound 6i

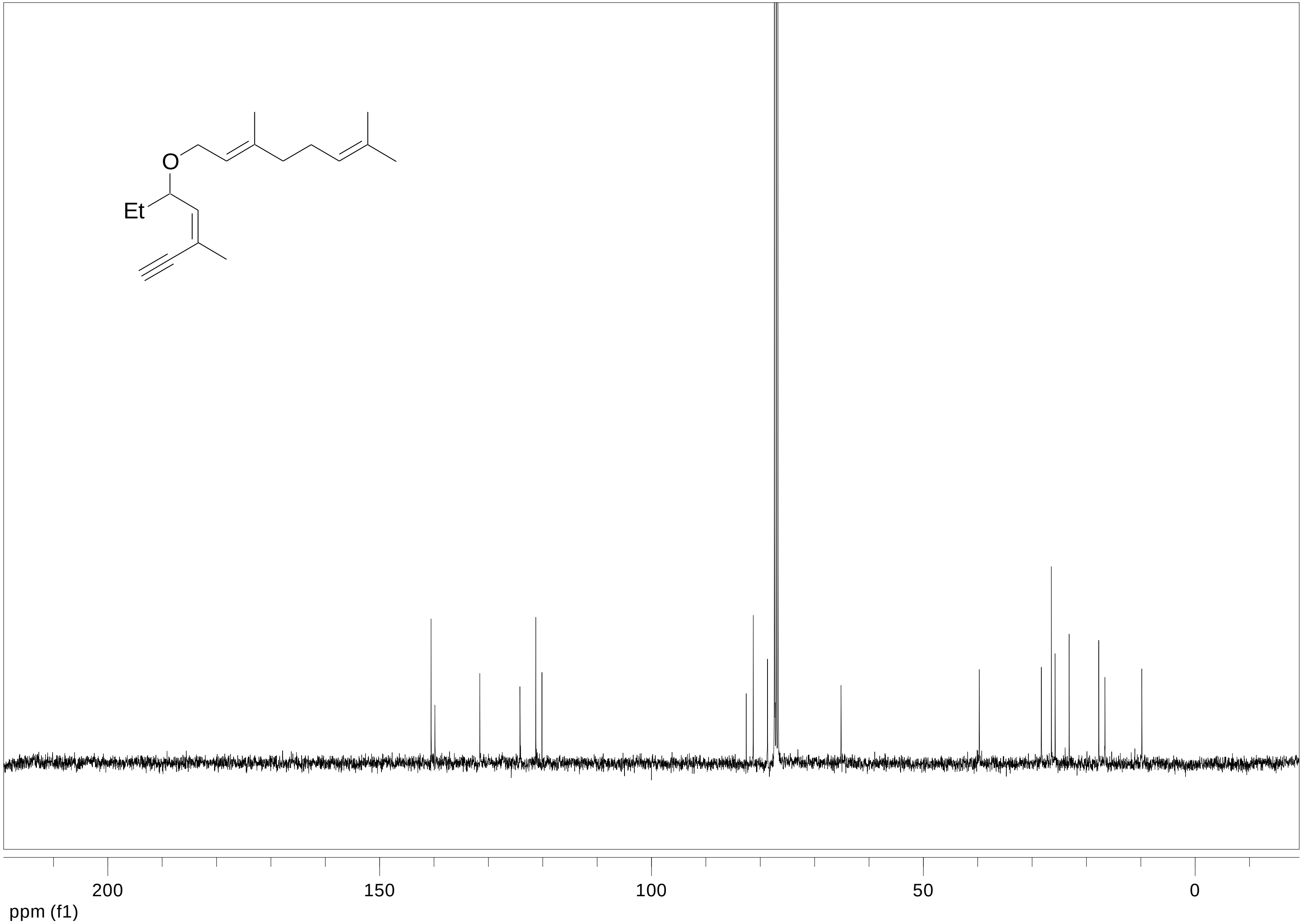

Compound 6i

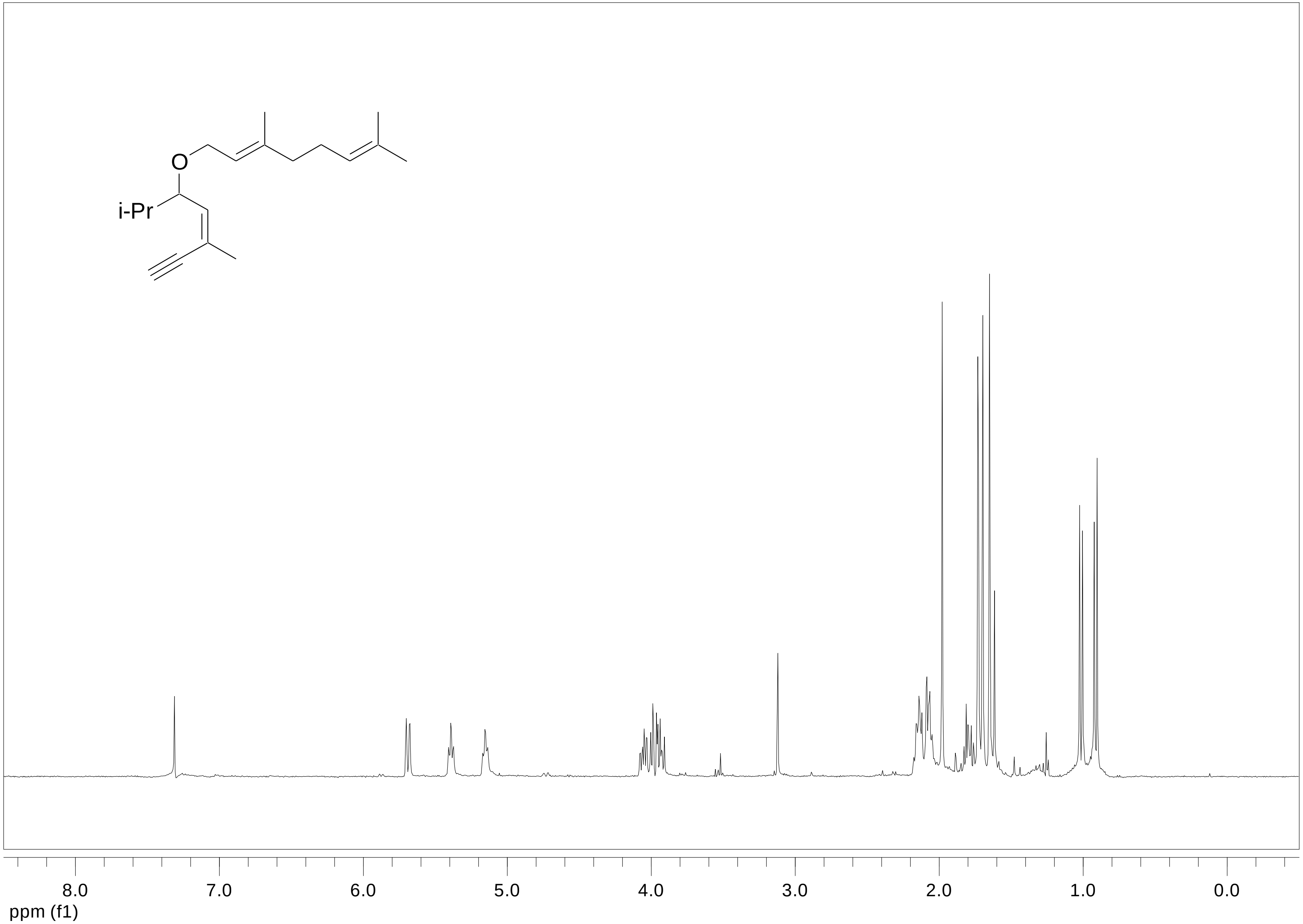

Compound 6j

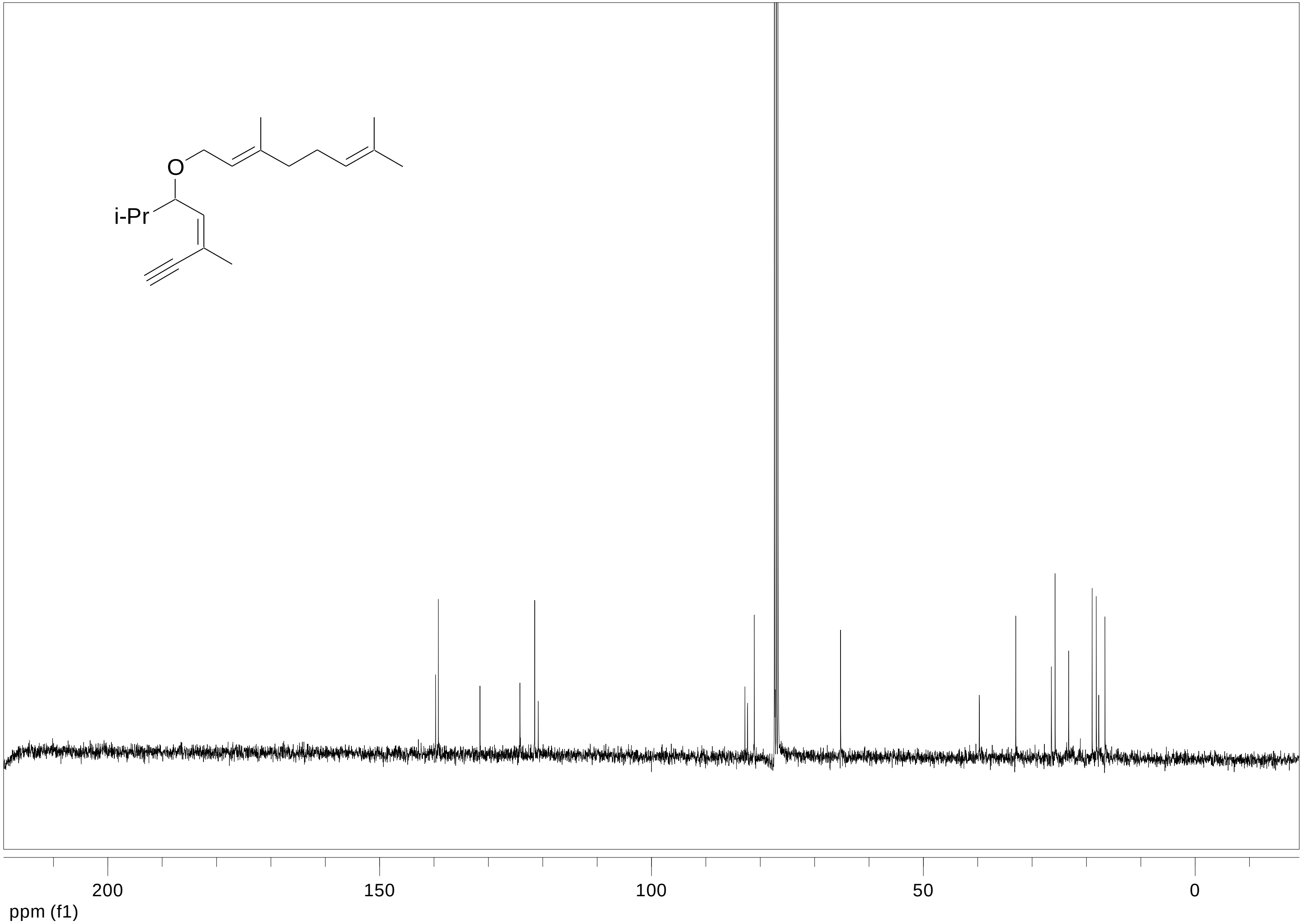

Compound 6j

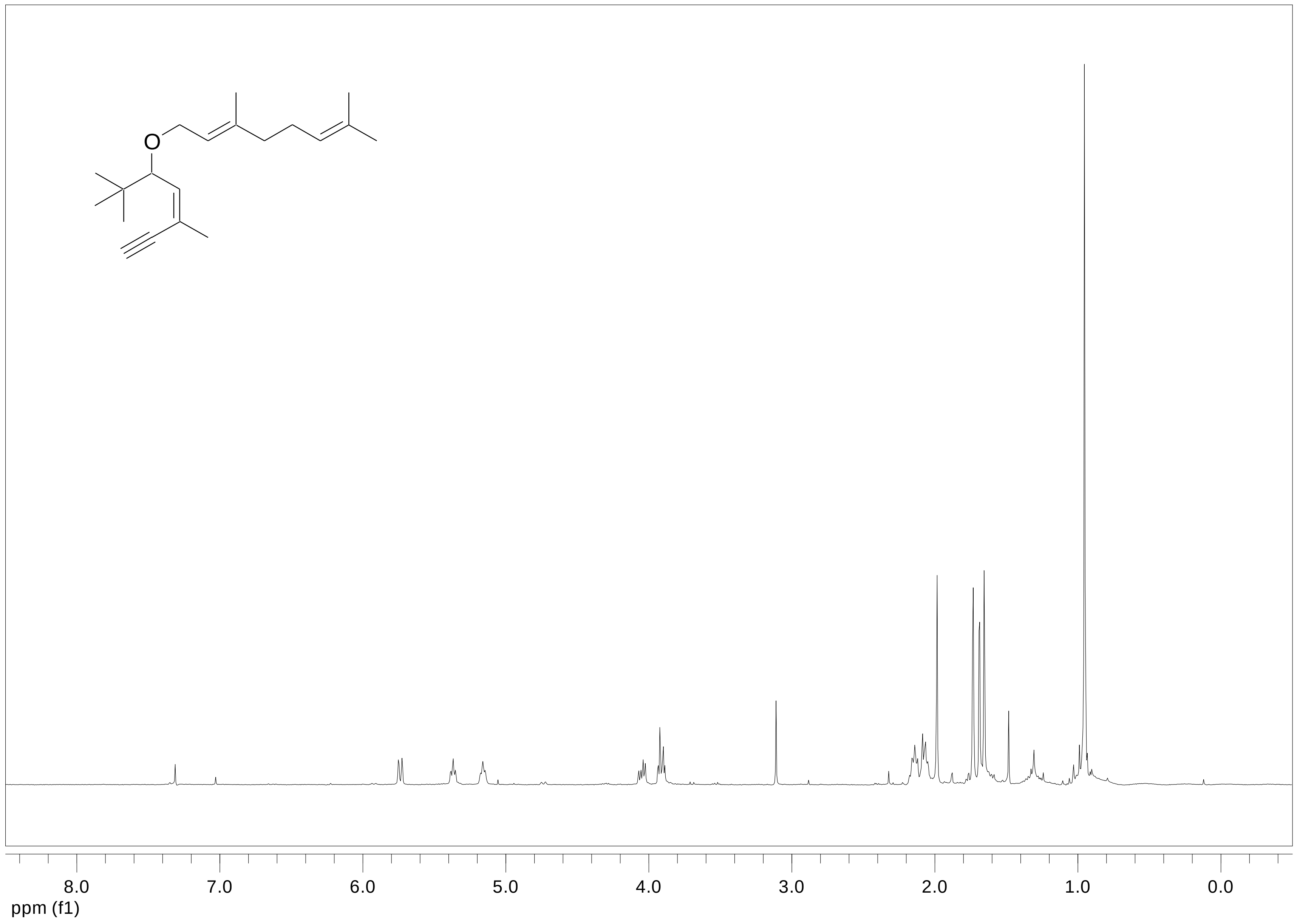

Compound 6k

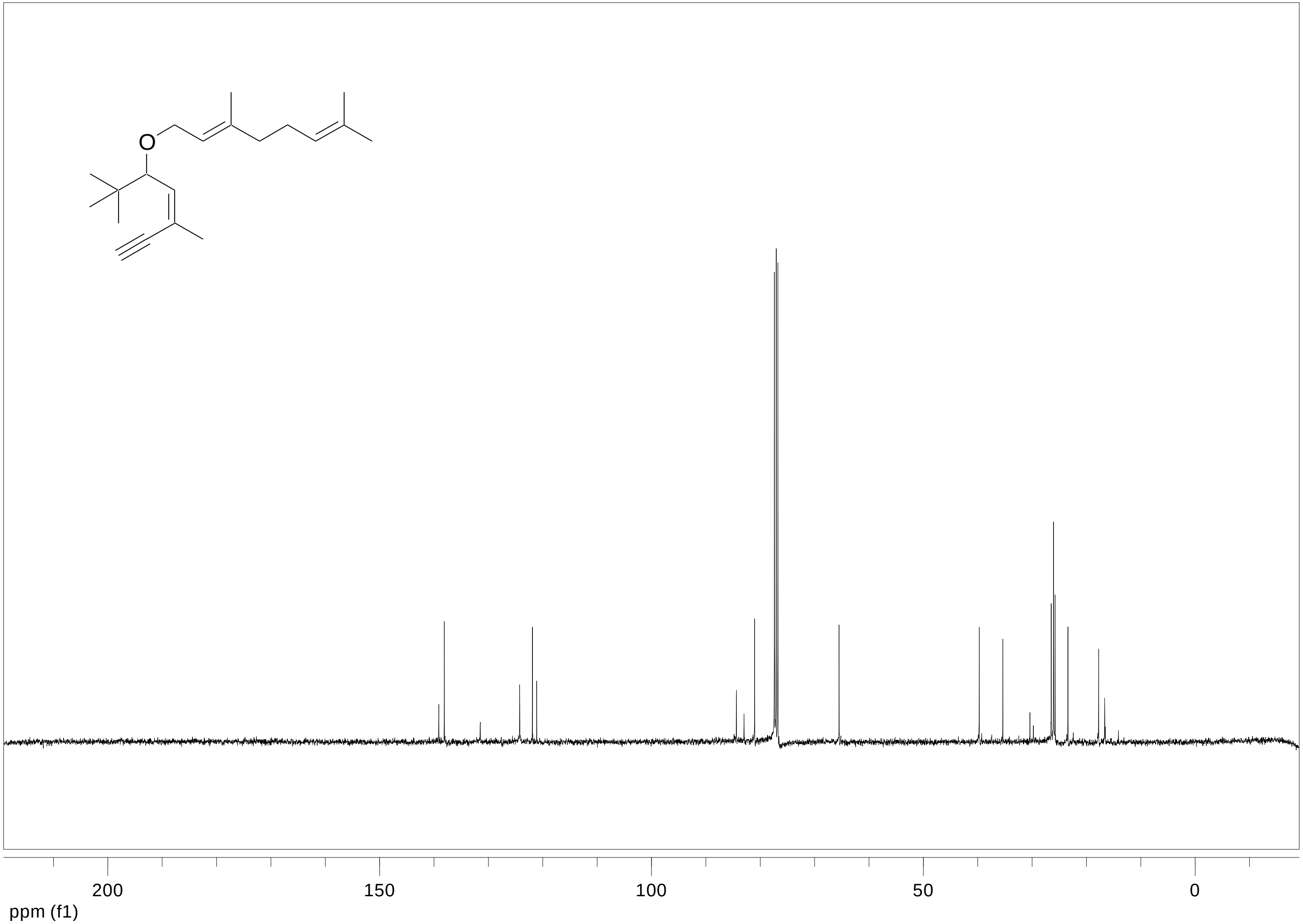

Compound 6k

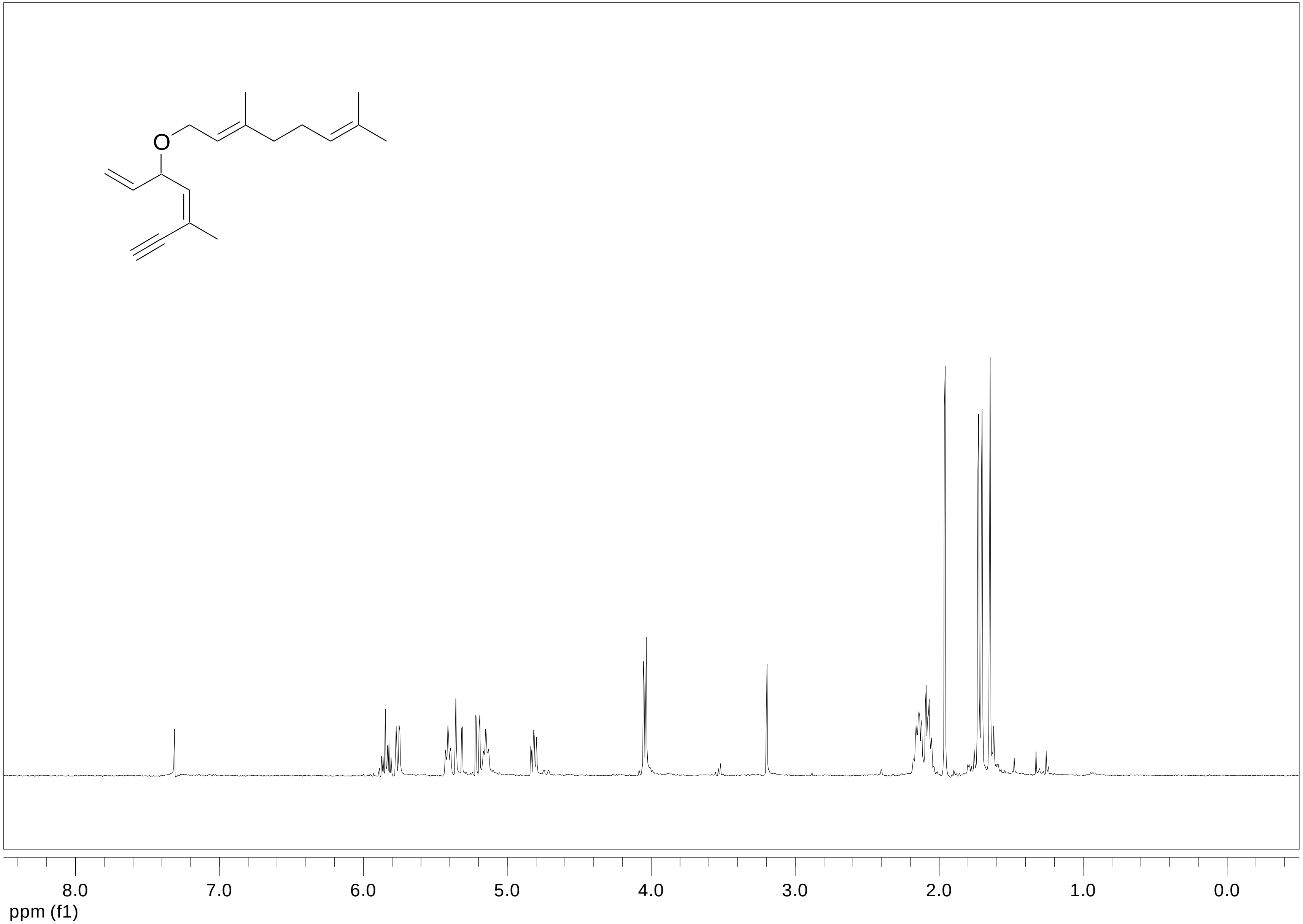

Compound 6l

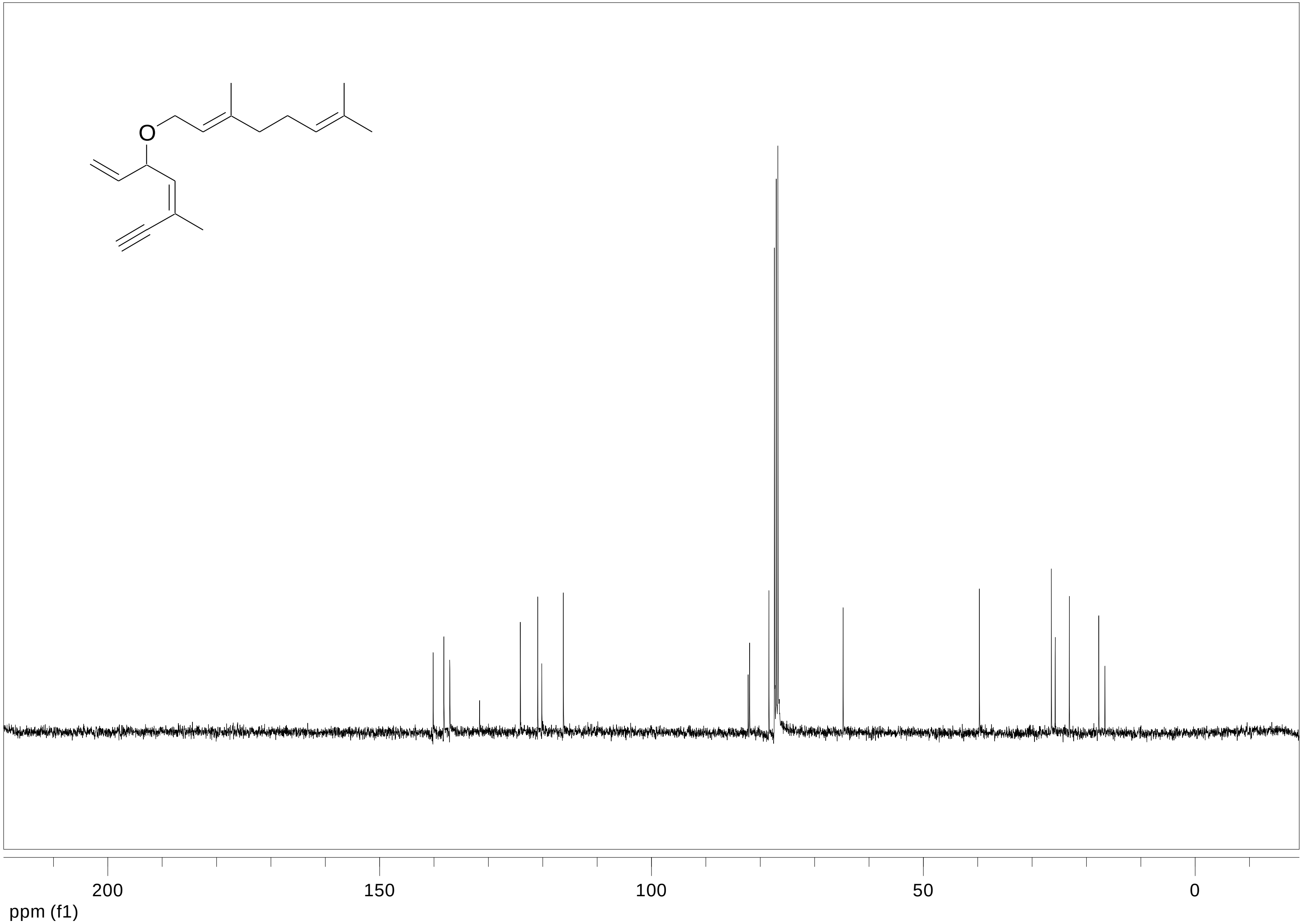

Compound 6l

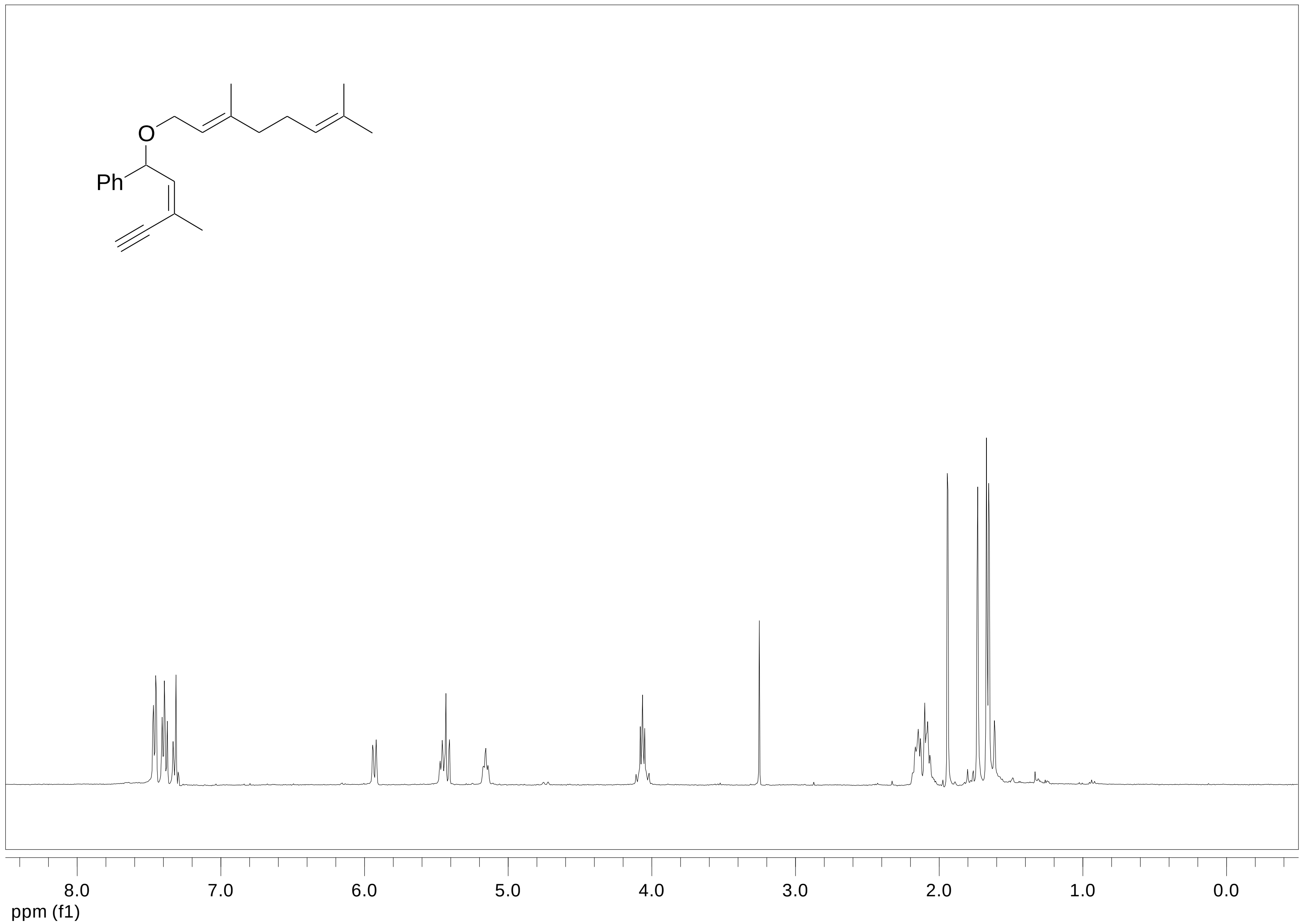

Compound 6m

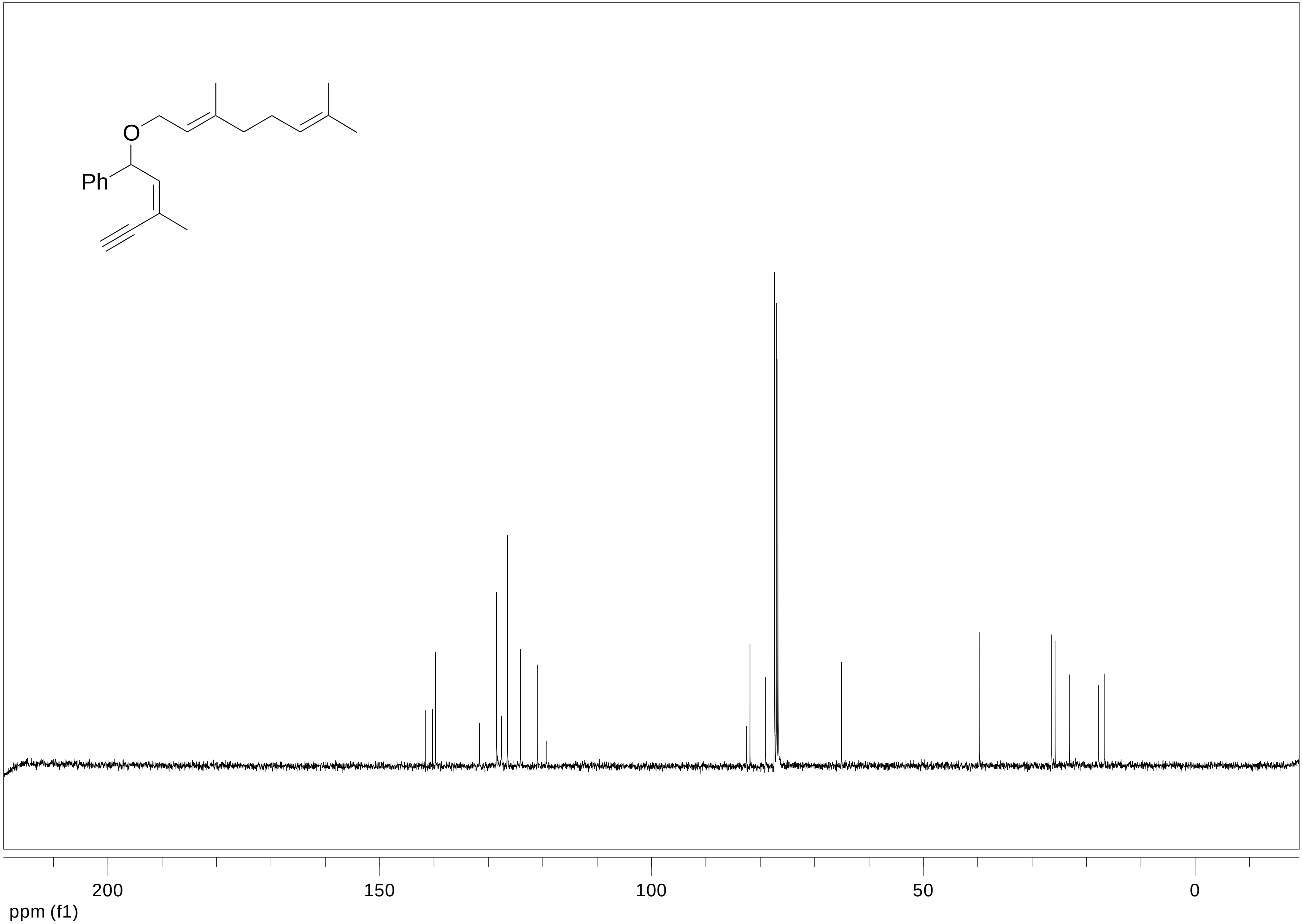

Compound 6m

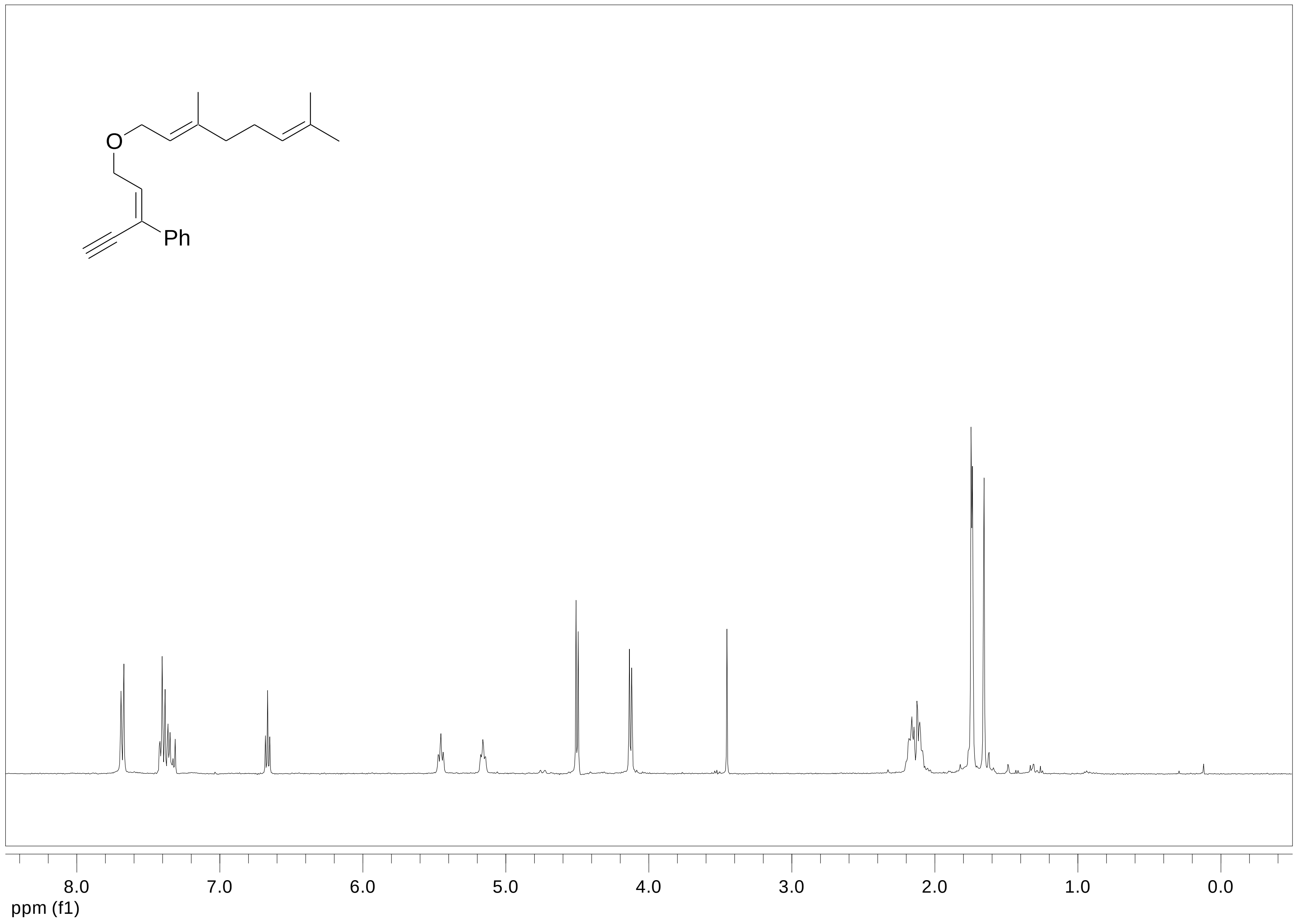

Compound 6n

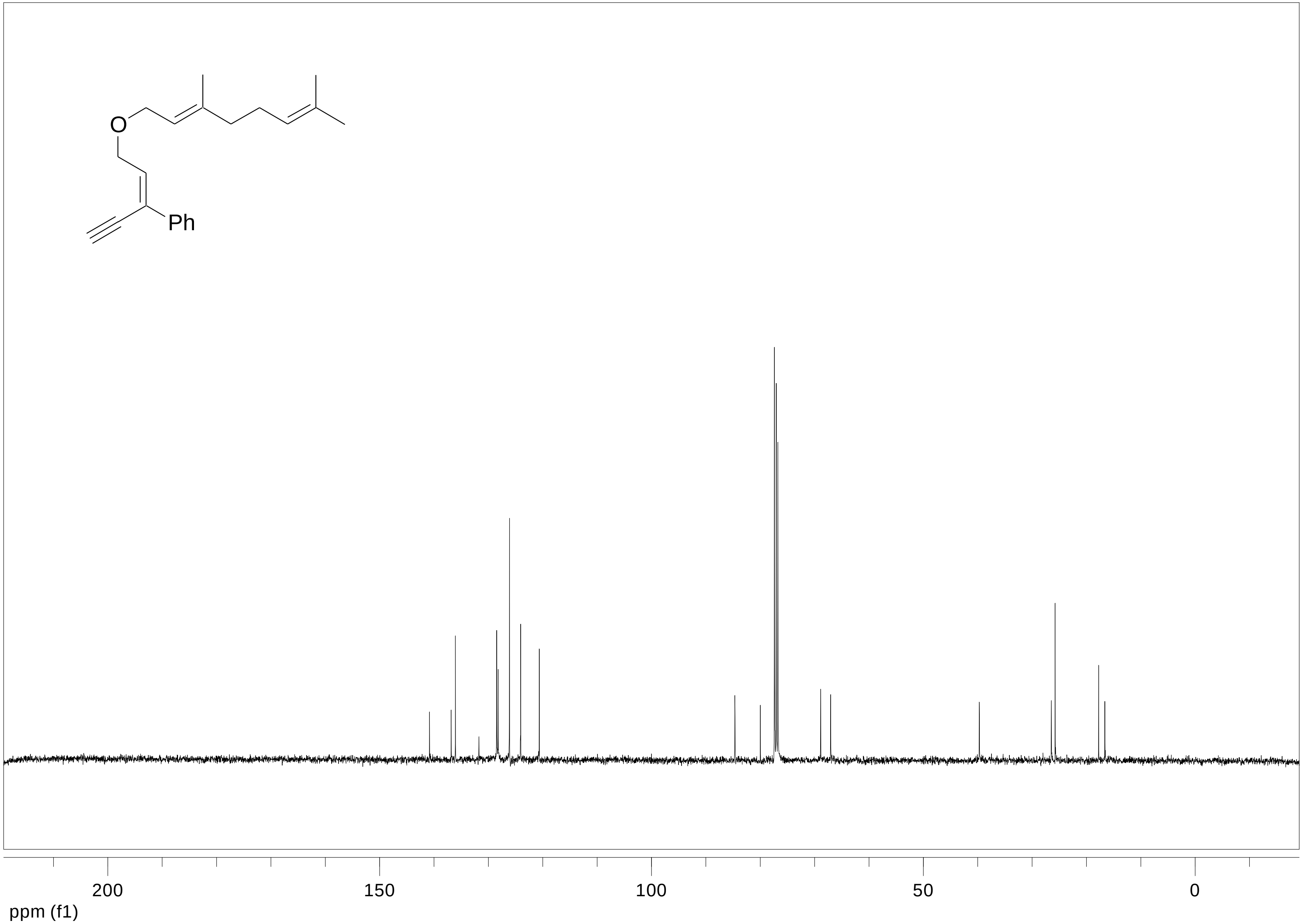

Compound 6n

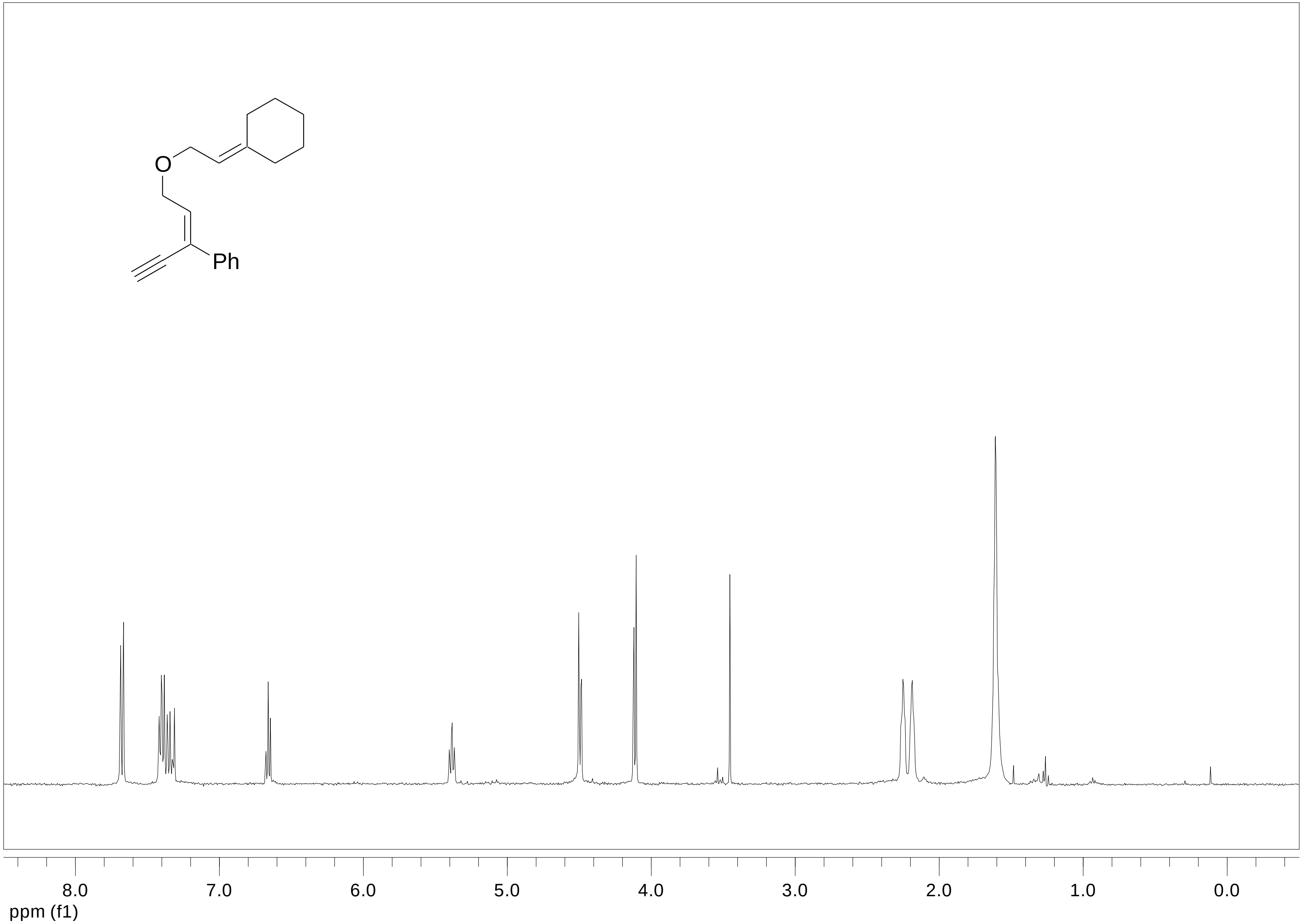

Compound 6o

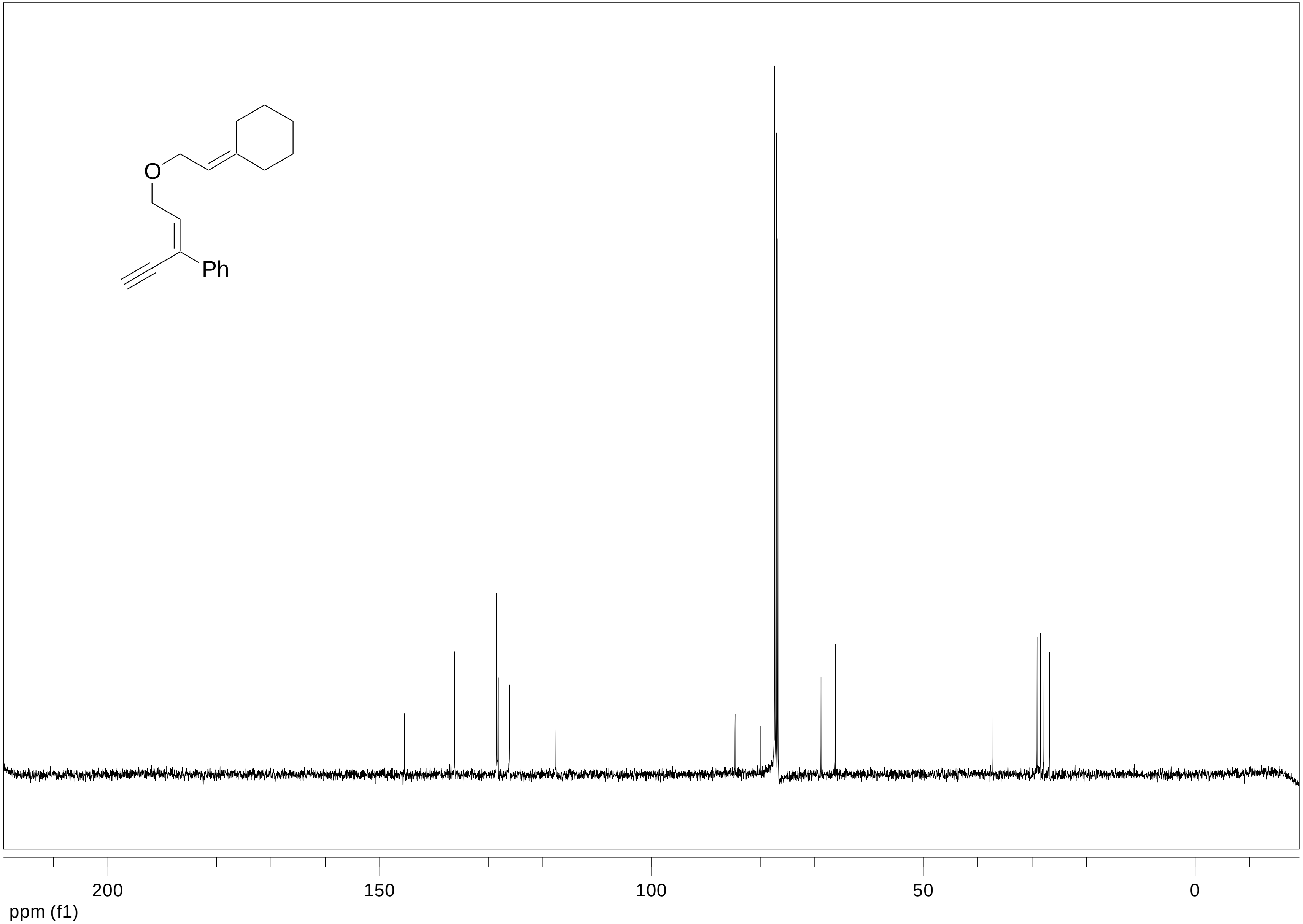

Compound 6o

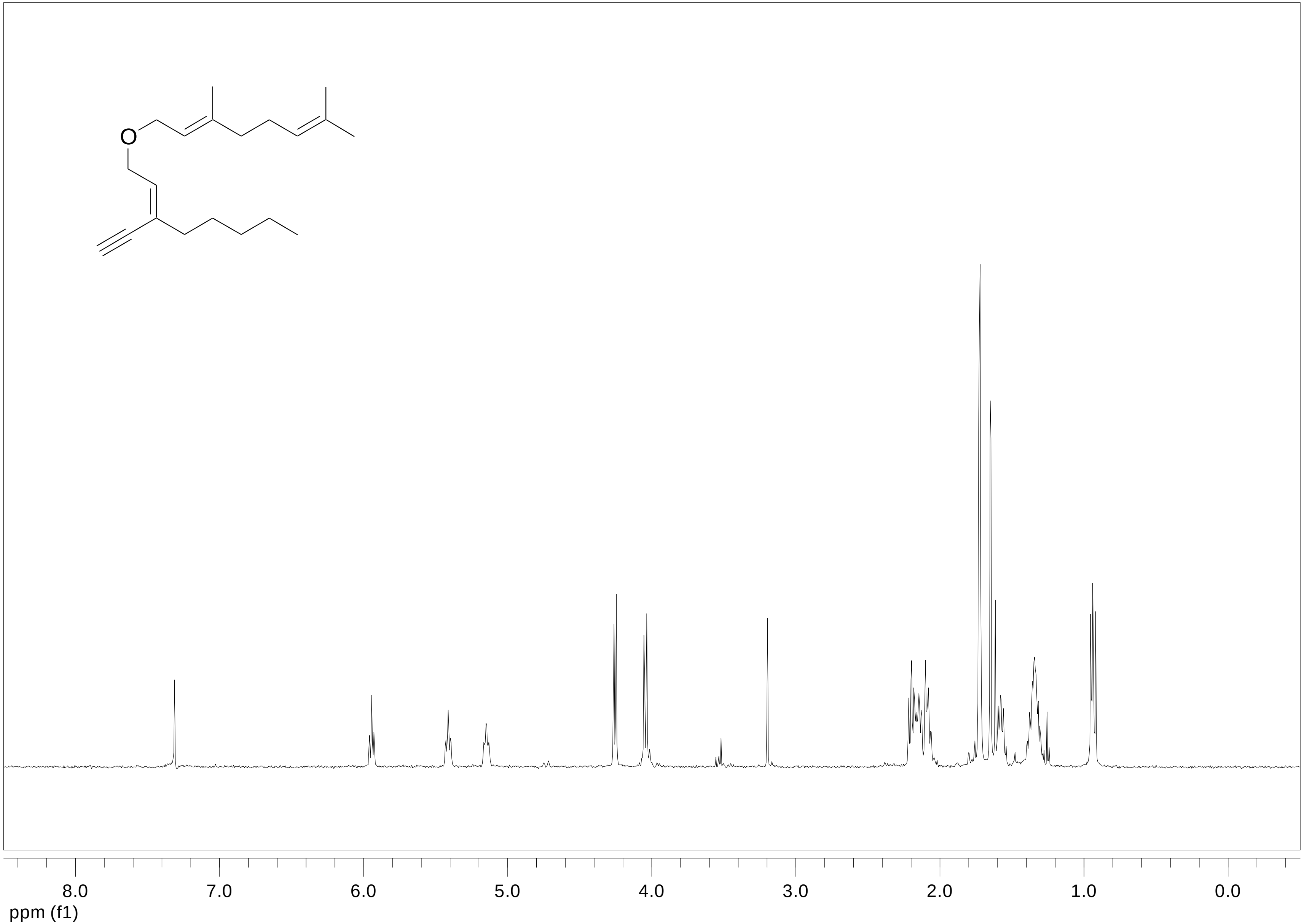

Compound 6p

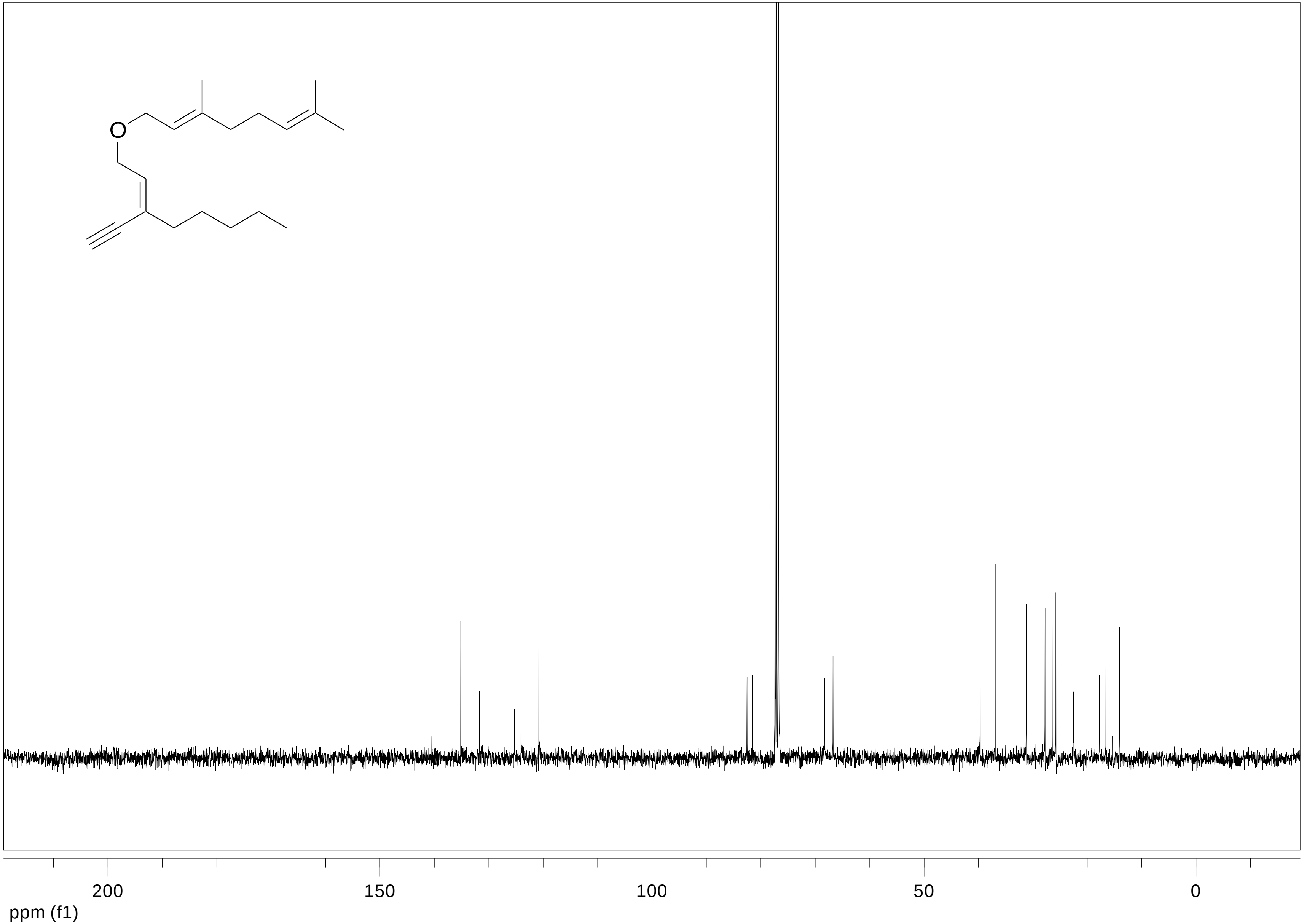

Compound 6p

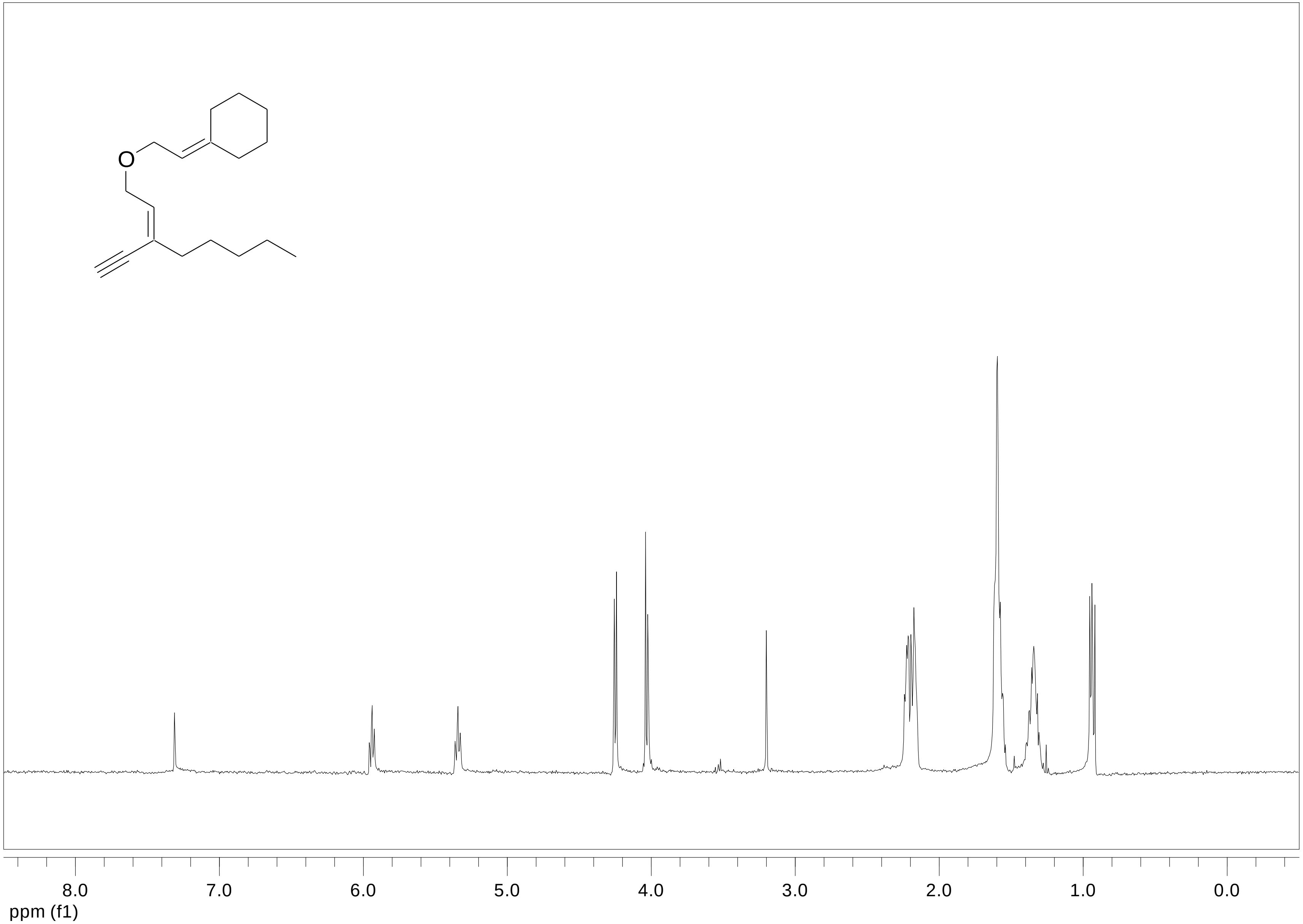

Compound 6q

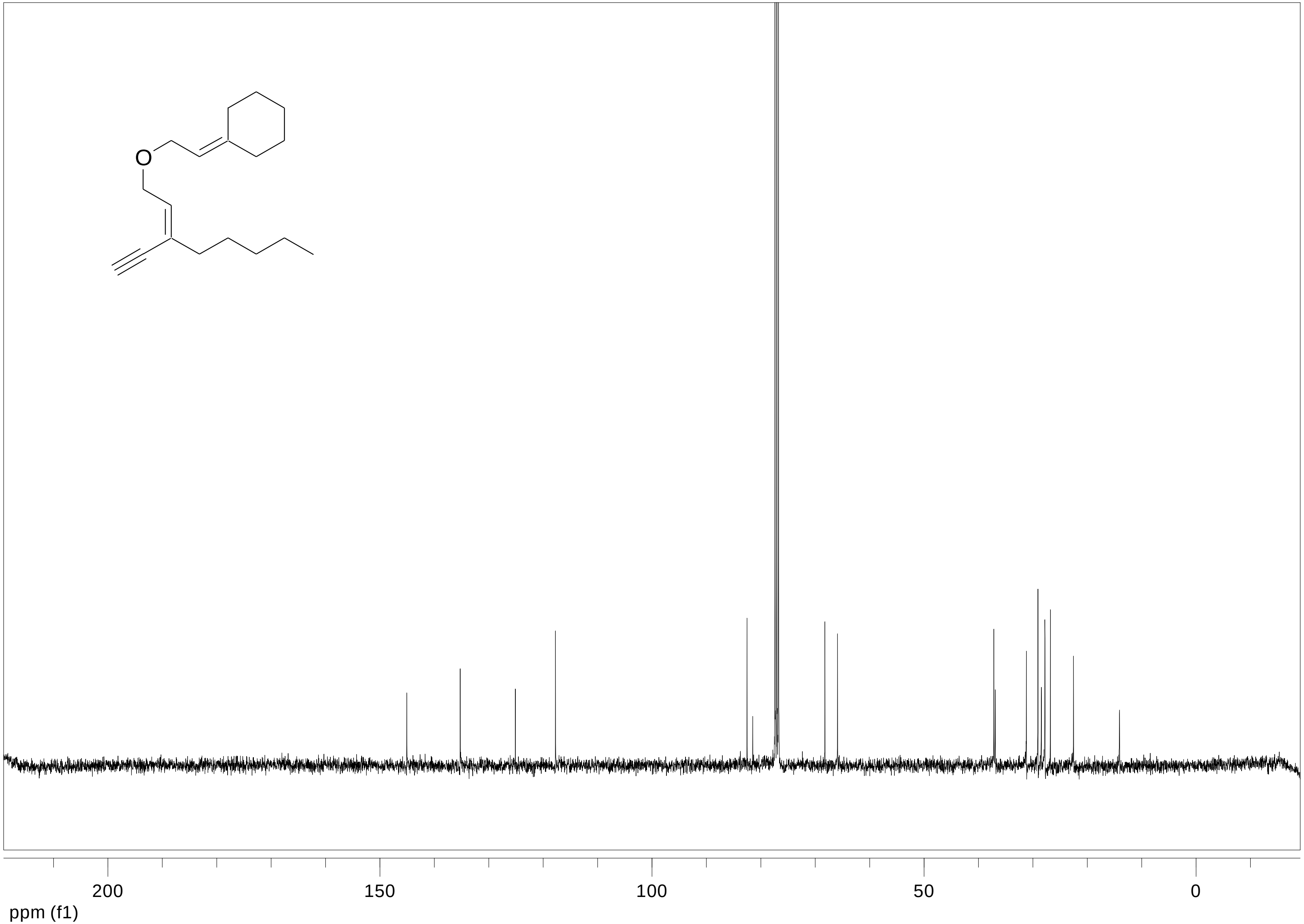

Compound 6q

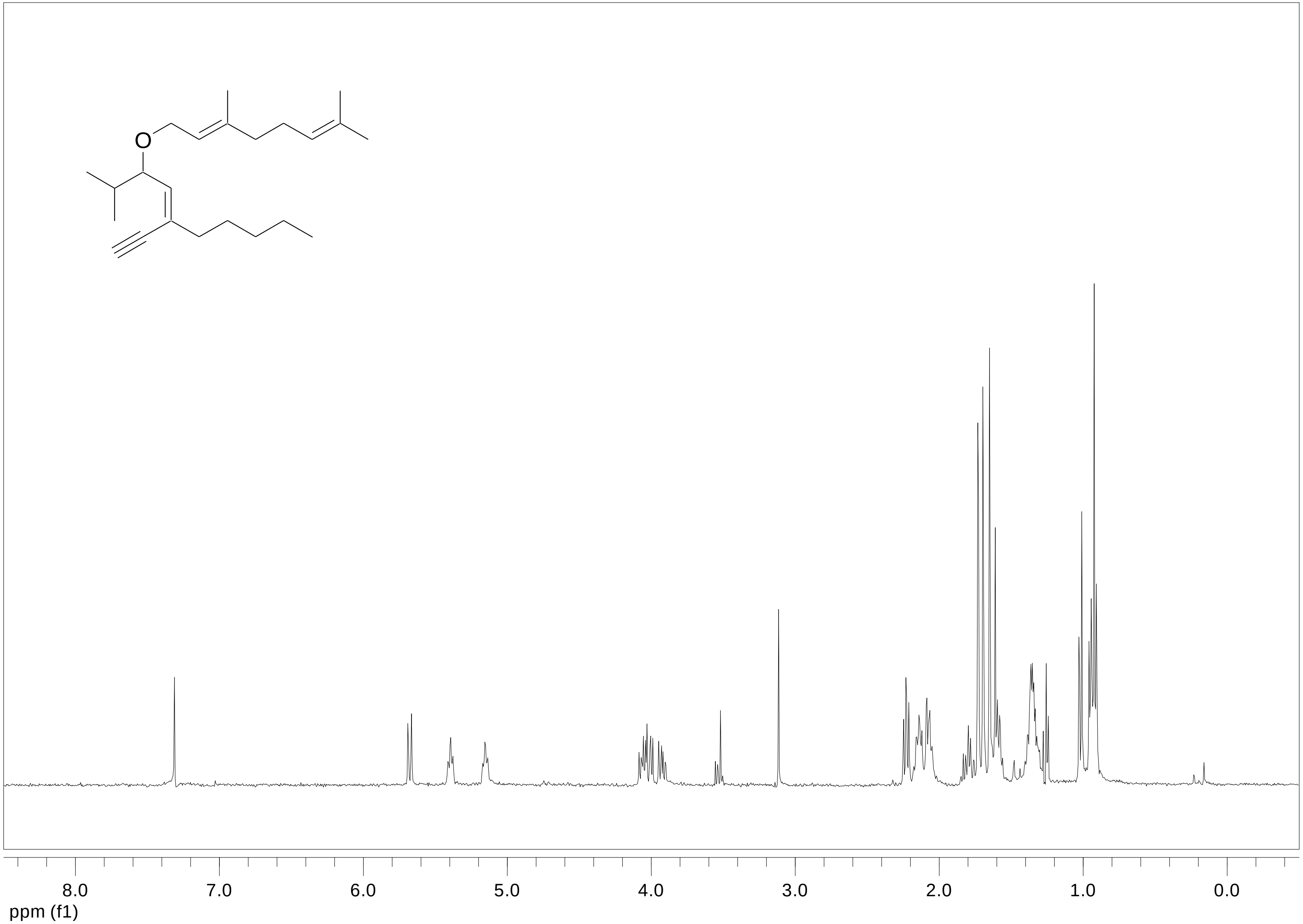

Compound 6r

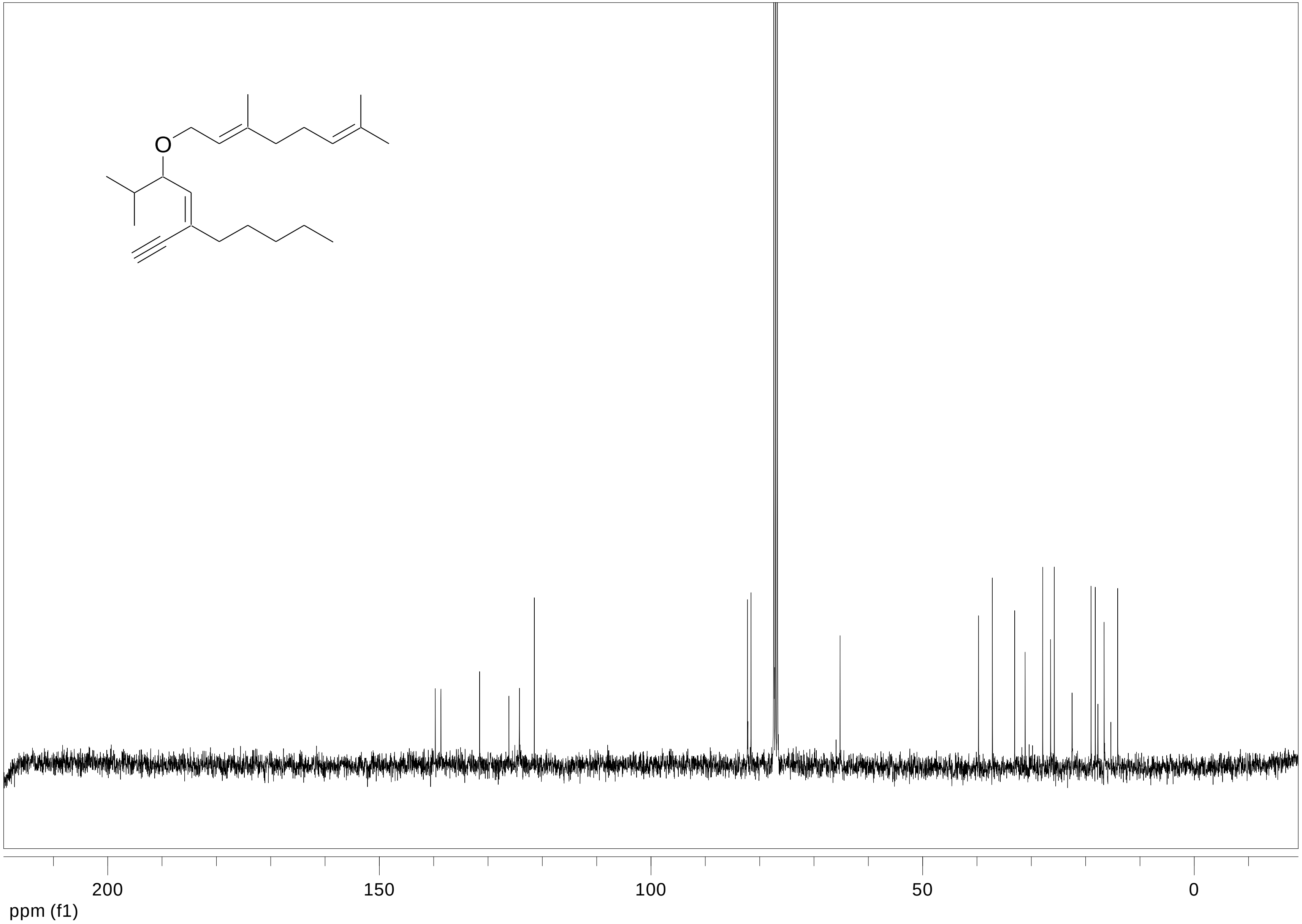

Compound 6r

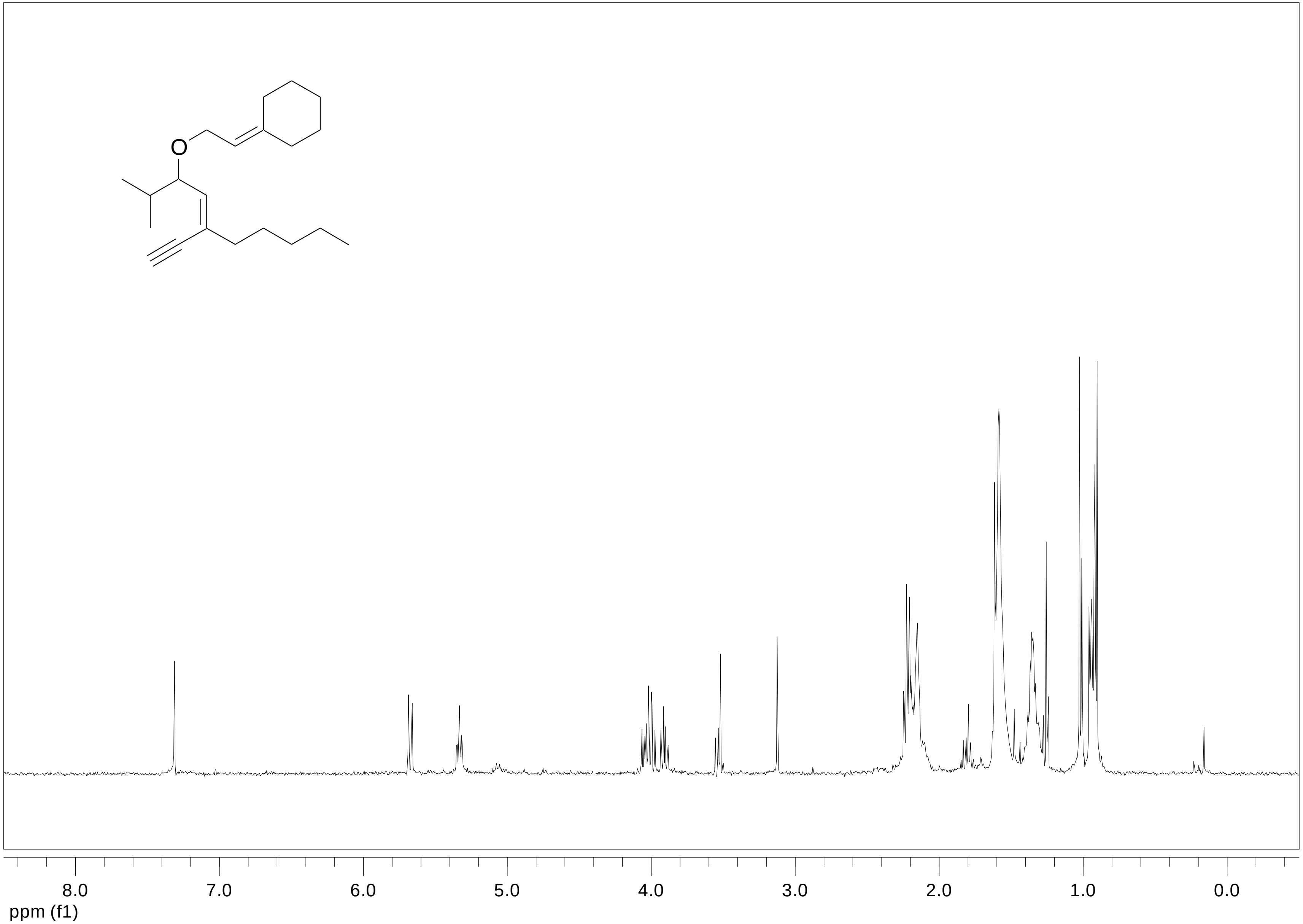

Compound 6s

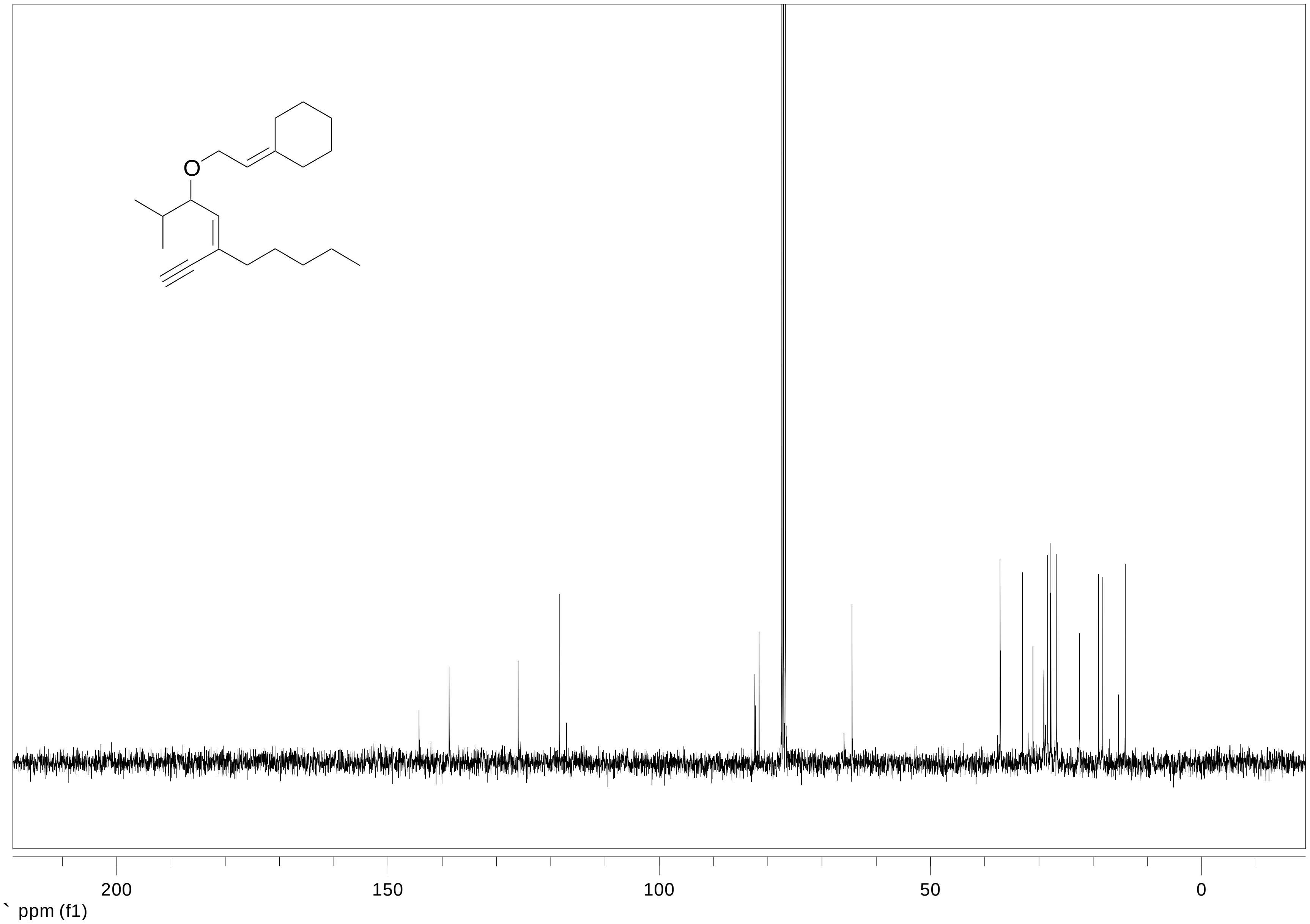

Compound 6s
