# Supplementary material for: Gold(I)-catalyzed formation of furans by a Claisen-type rearrangement of ynenyl allyl ethers
Source: Beilstein J Org Chem. 2011 Jun 29;7:878–85. doi: 10.3762/bjoc.7.100 (PMC3135071; doi:10.3762/bjoc.7.100)
Supplement: File 3 — NMR spectral data for products 7a–s. [file Beilstein_J_Org_Chem-07-878-s003.pdf]

Supporting Information

for

Gold(I)-catalyzed formation of furans by a Claisen-type rearrangement of ynenyl allyl ethers

Florin M. Istrate and Fabien Gagosz*

Address: Département de Chimie, UMR 7652, CNRS/Ecole Polytechnique, 91128 Palaiseau, France

Email: Fabien Gagosz - gagosz@dcso.polytechnique.fr

* Corresponding Author

Spectra of furans 7a-s

Spectra of compound **7a** p S2-S3
Spectra of compound **7b** p S4-S5
Spectra of compound **7c** p S6-S7
Spectra of compound **7d** p S8-S9
Spectra of compound **7e** p S10-S11
Spectra of compound **7f** p S12-S13
Spectra of compound **7g** p S14-S15
Spectra of compound **7h** p S16-S17
Spectra of compound **7i** p S18-S19
Spectra of compound **7j** p S20-S21
Spectra of compound **7k** p S22-S23
Spectra of compound **7l** p S24-S25
Spectra of compound **7m** p S26-S27
Spectra of compound **7n** p S28-S29
Spectra of compound **7o** p S30-S31
Spectra of compound **7p** p S32-S33
Spectra of compound **7q** p S34-S35
Spectra of compound **7r** p S36-S37
Spectra of compound **7s** p S38-S39

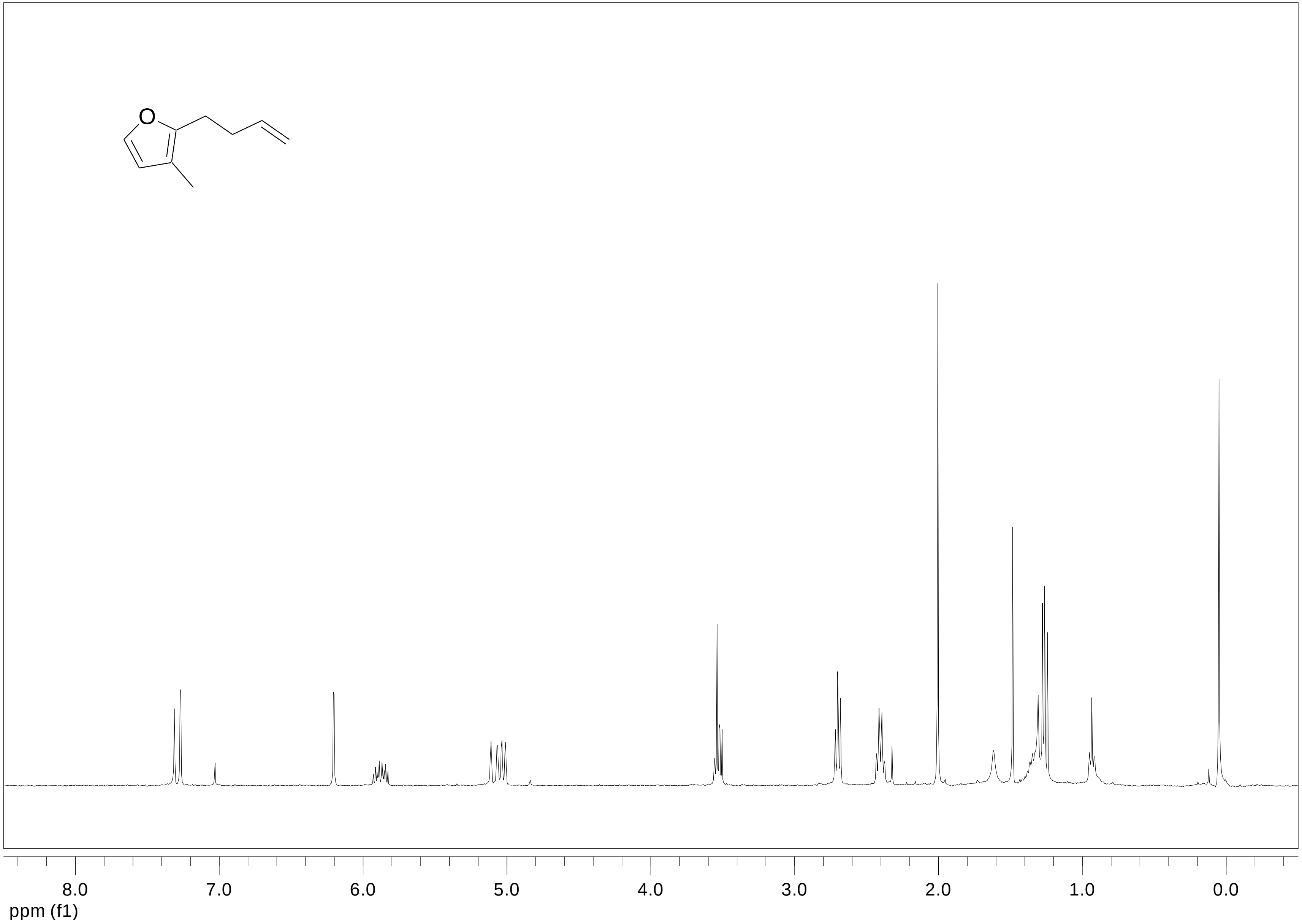

Compound 7a

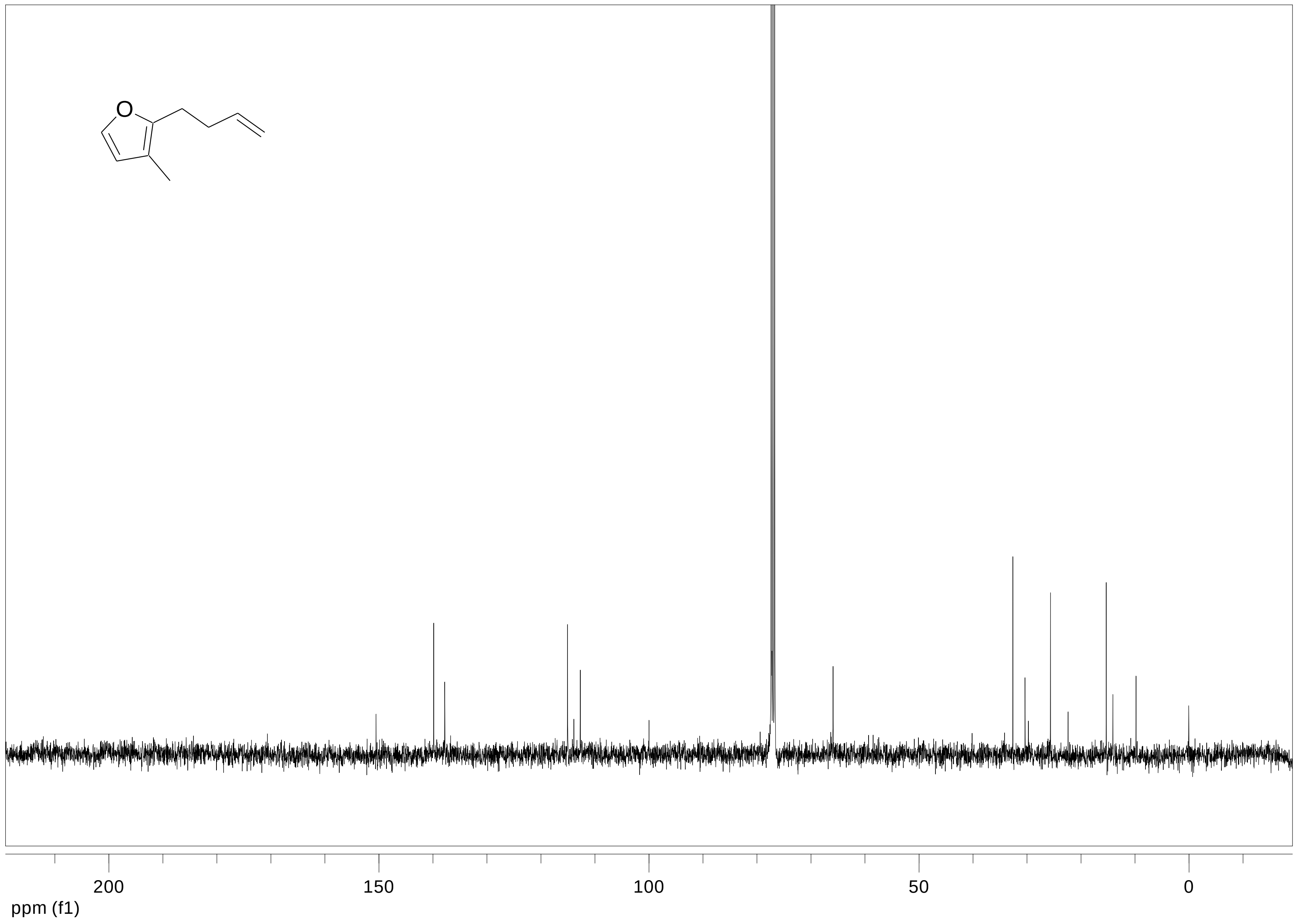

Compound 7a

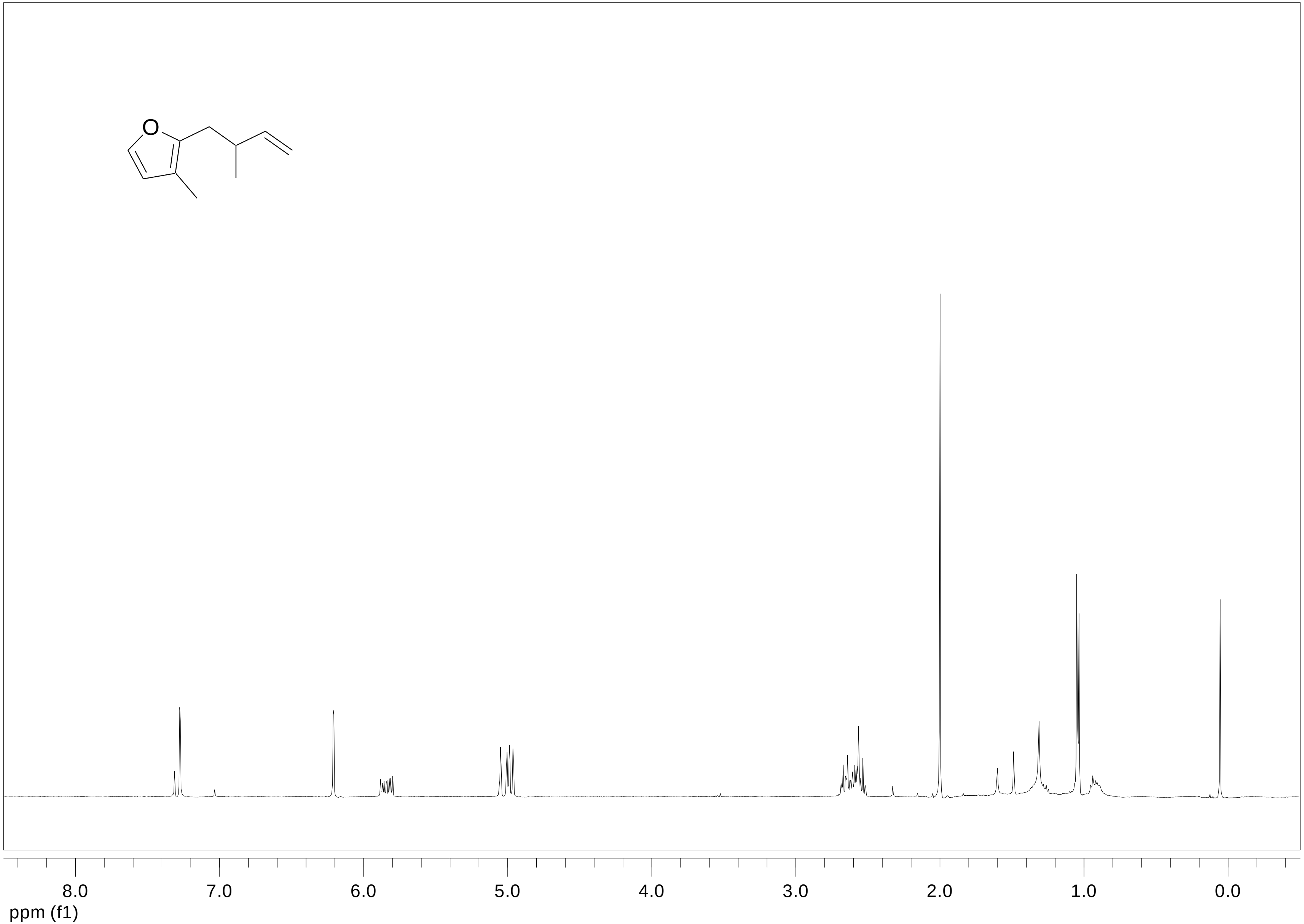

Compound 7b

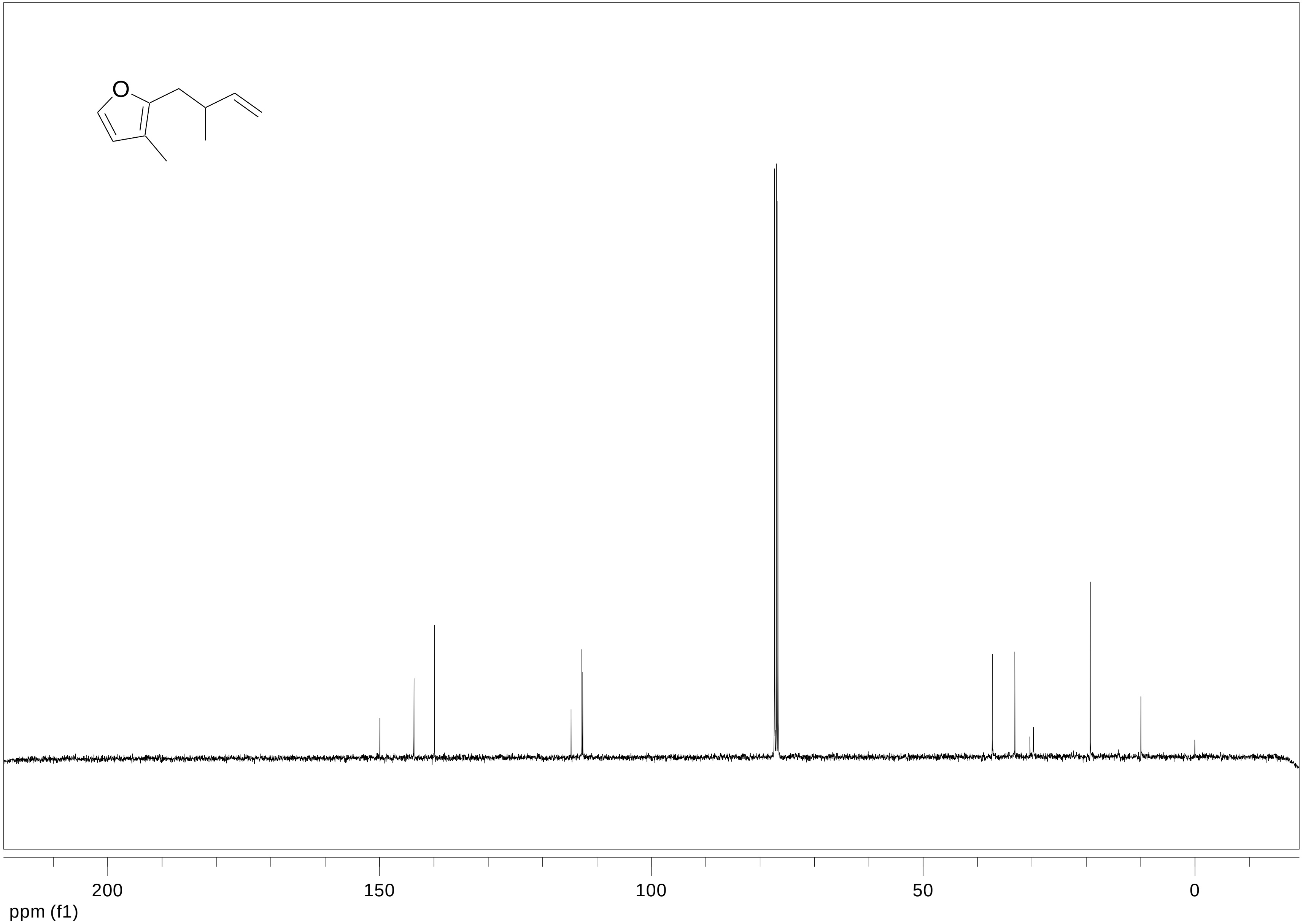

Compound 7b

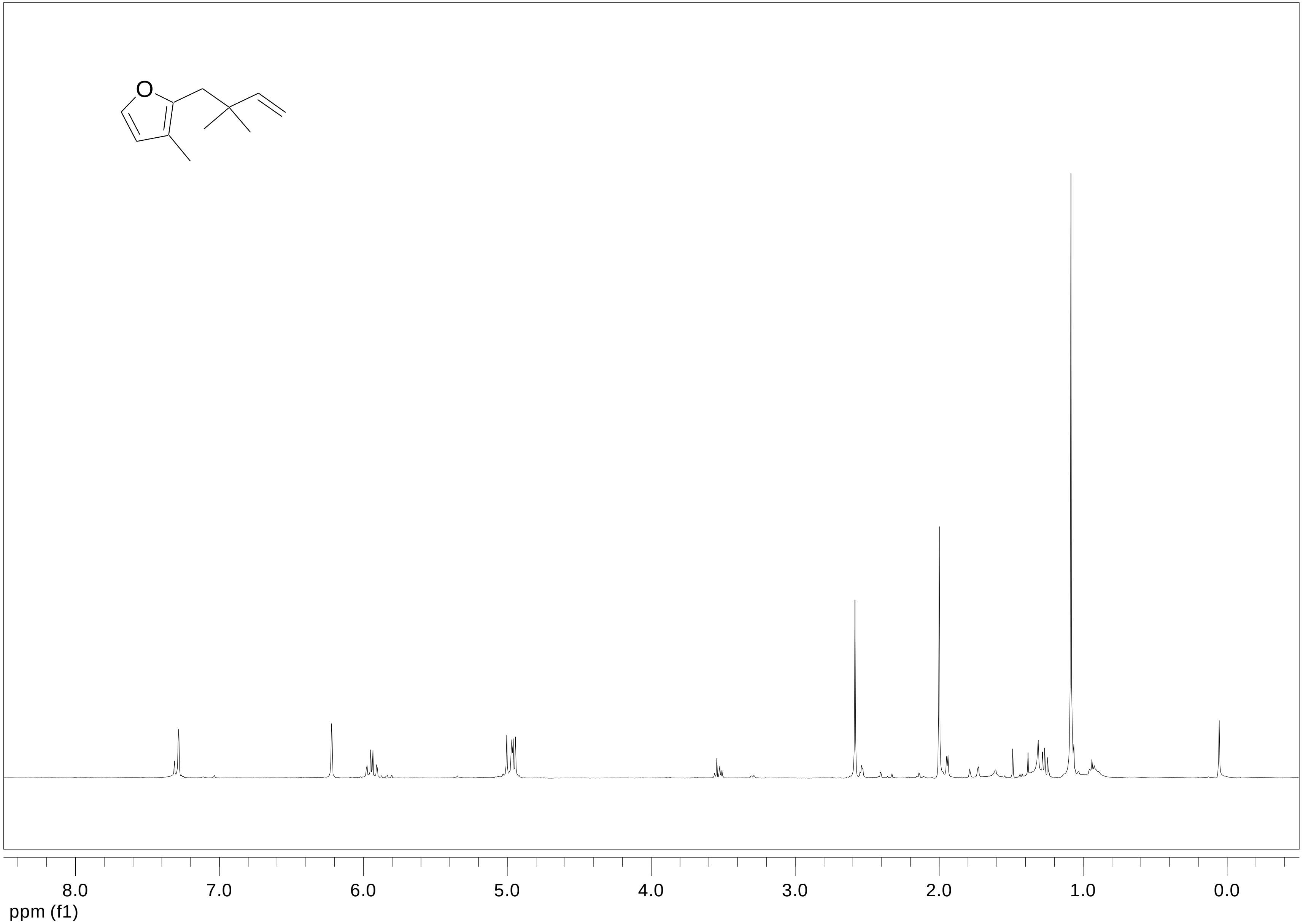

Compound 7c

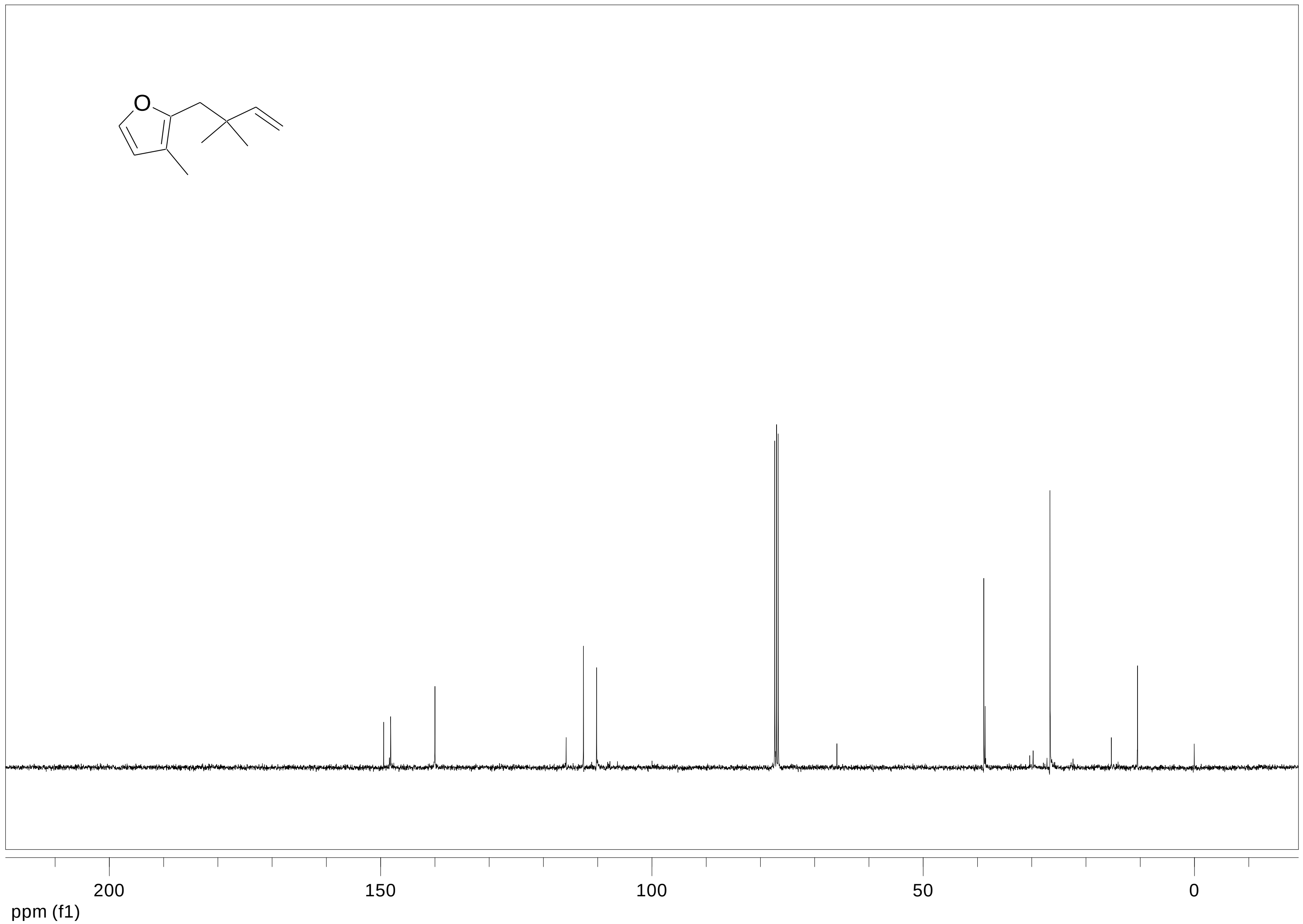

Compound 7c

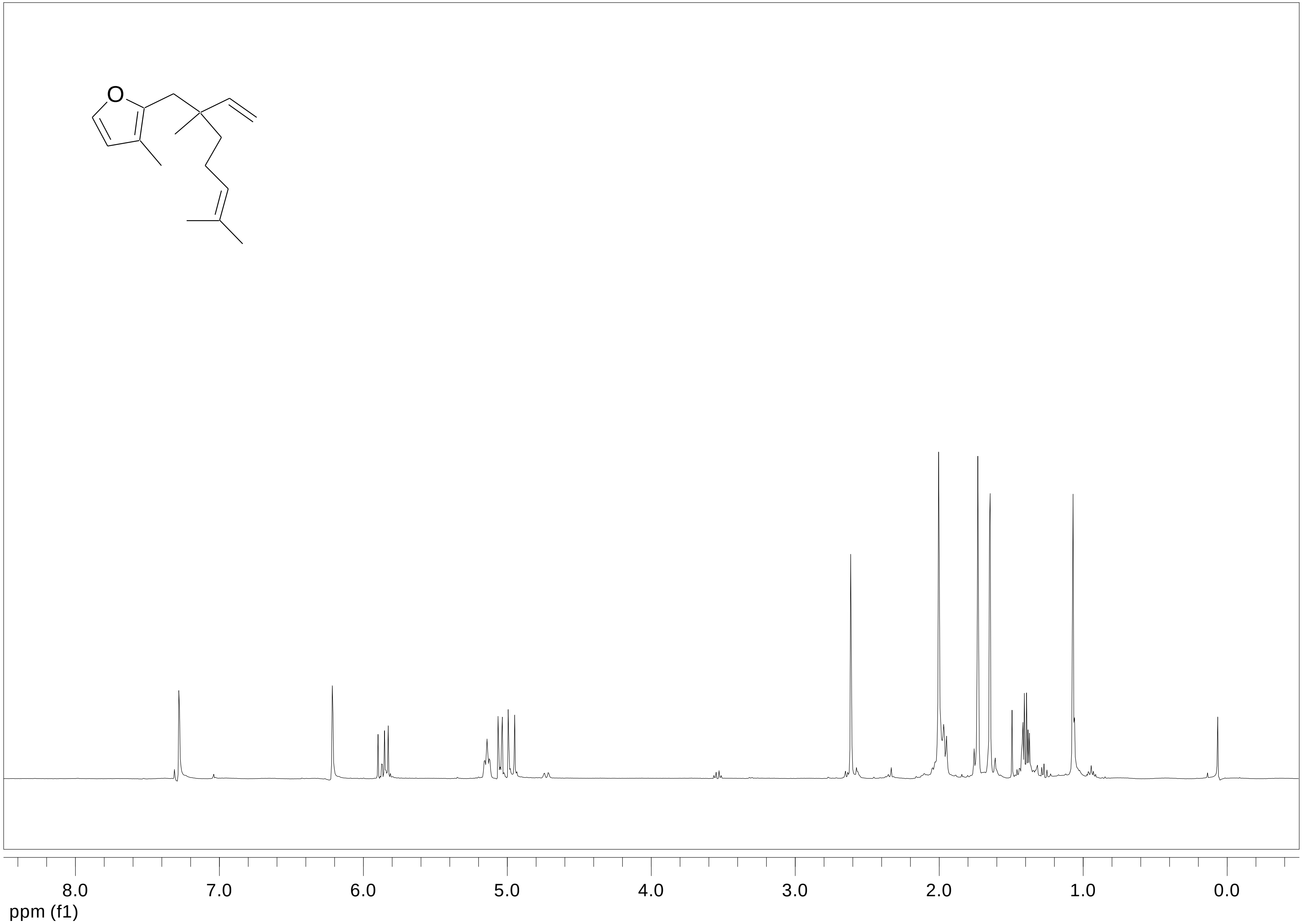

Compound 7d

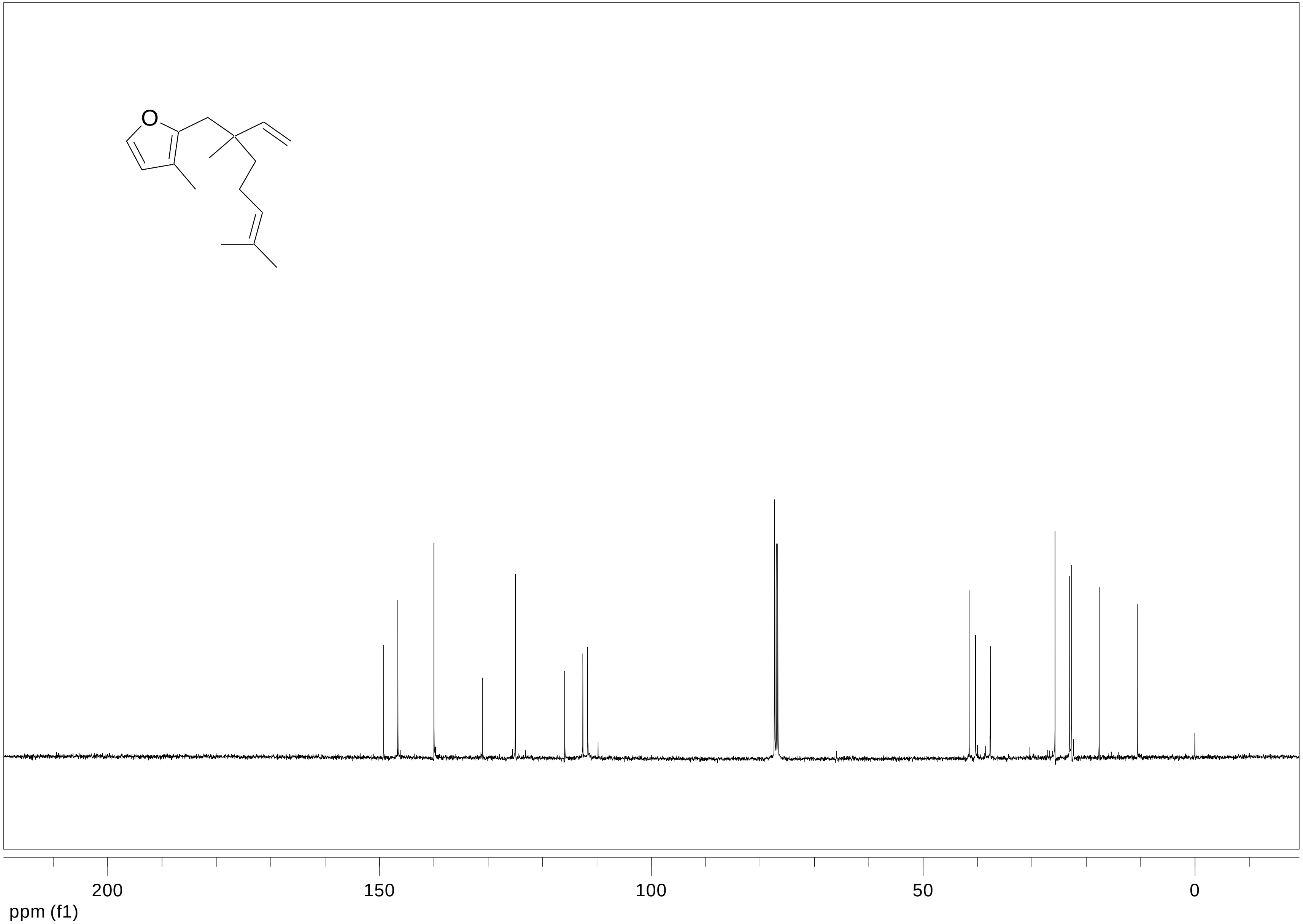

Compound 7d

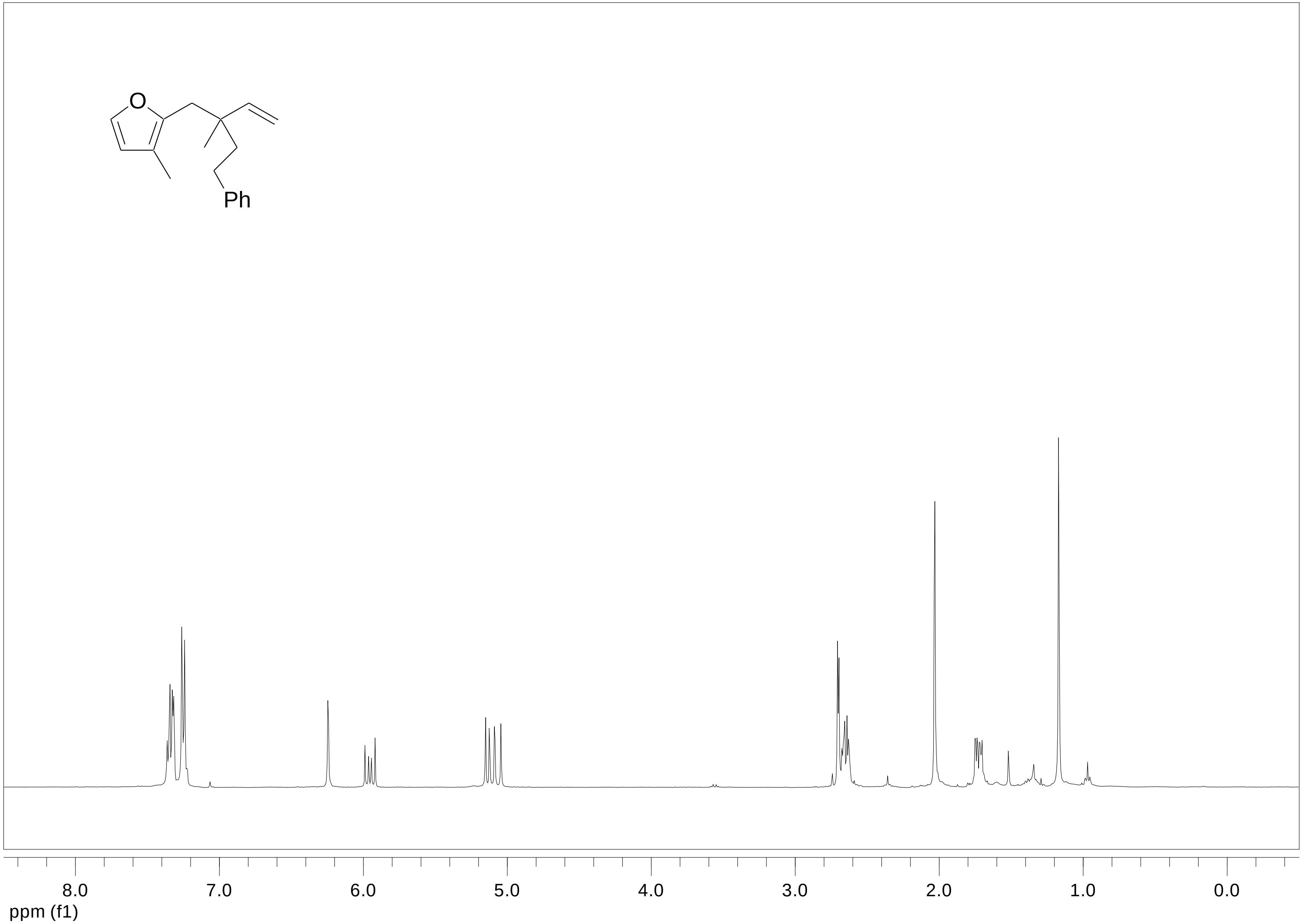

Compound 7e

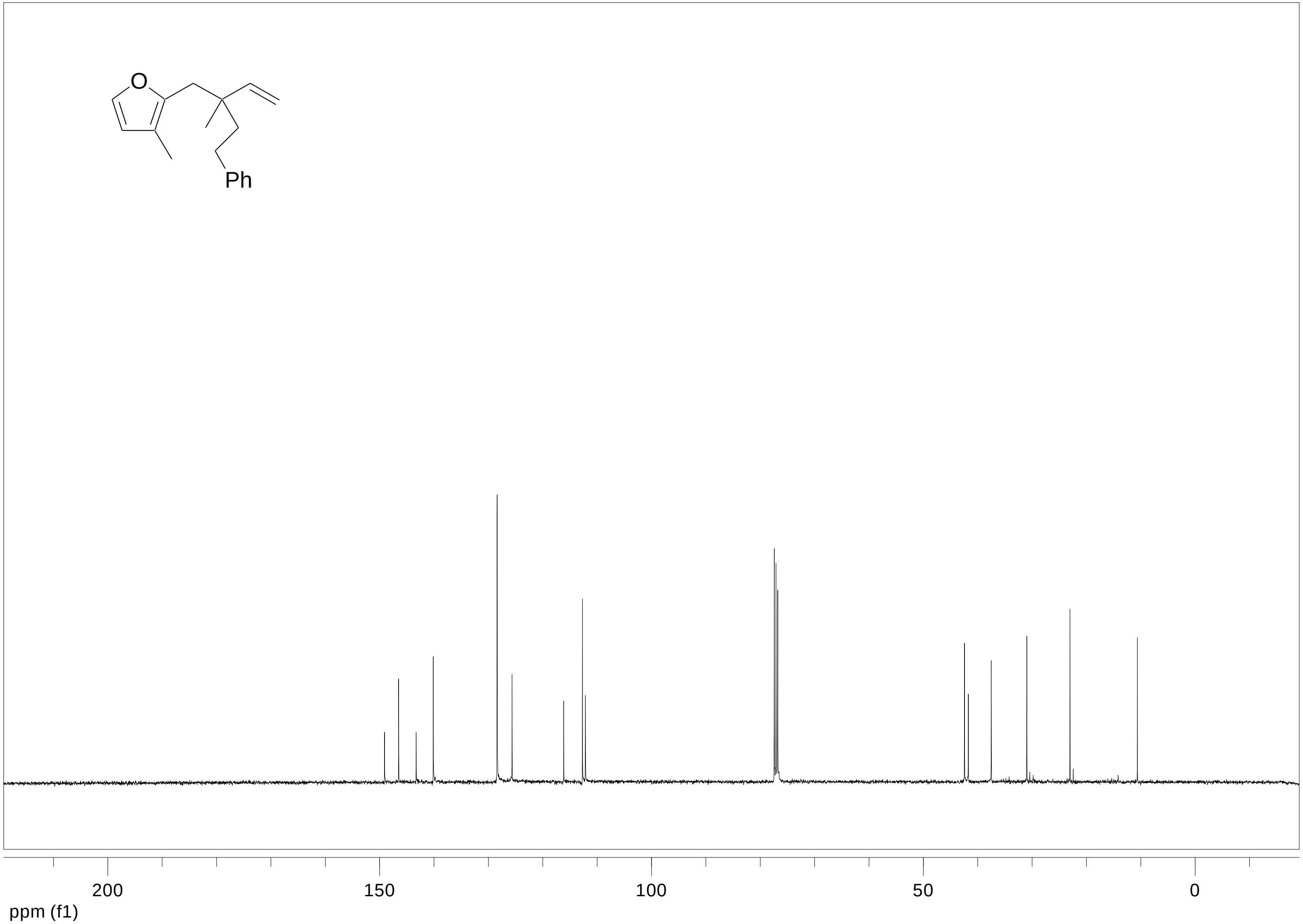

Compound 7e

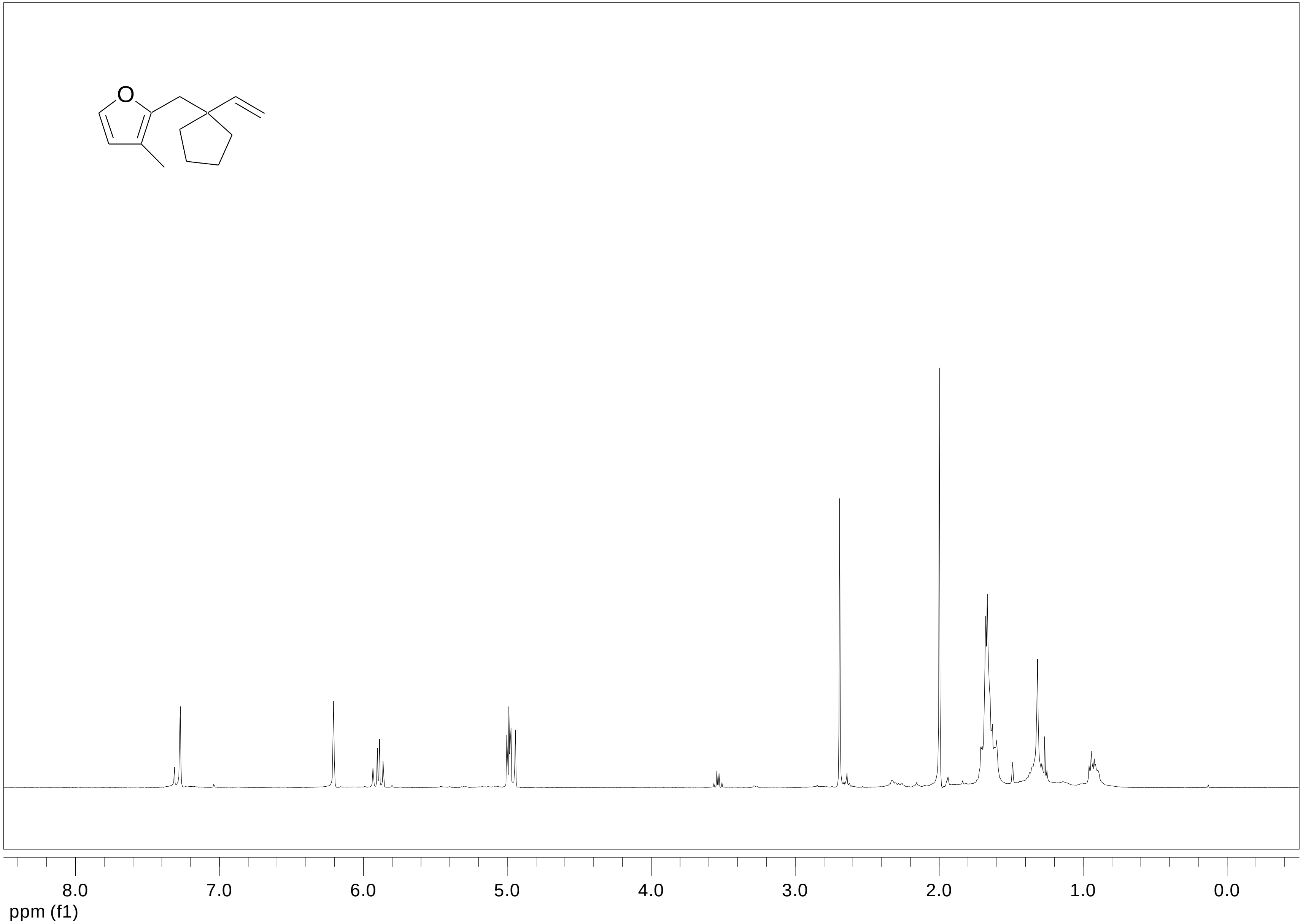

Compound 7f

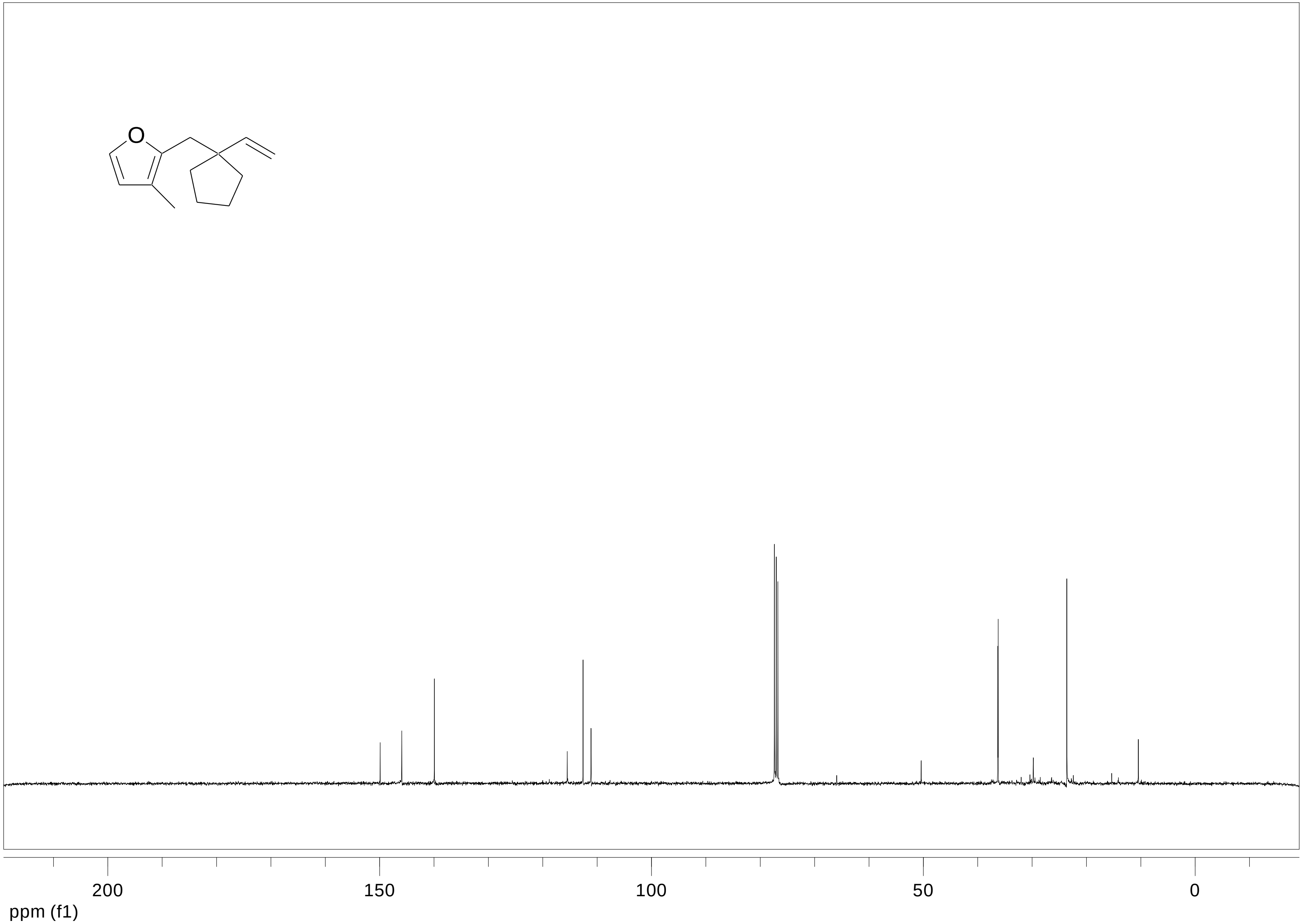

Compound 7f

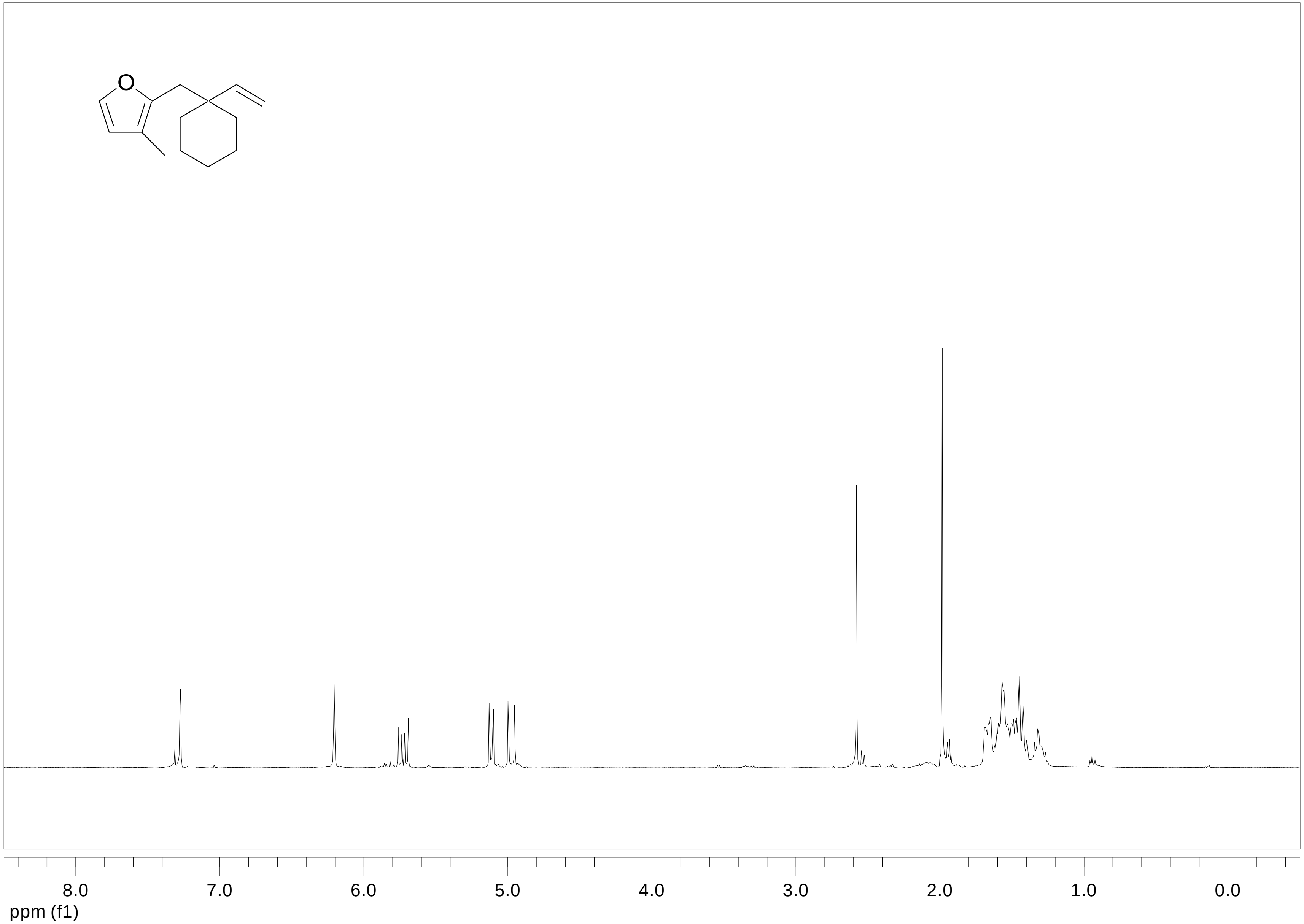

Compound 7g

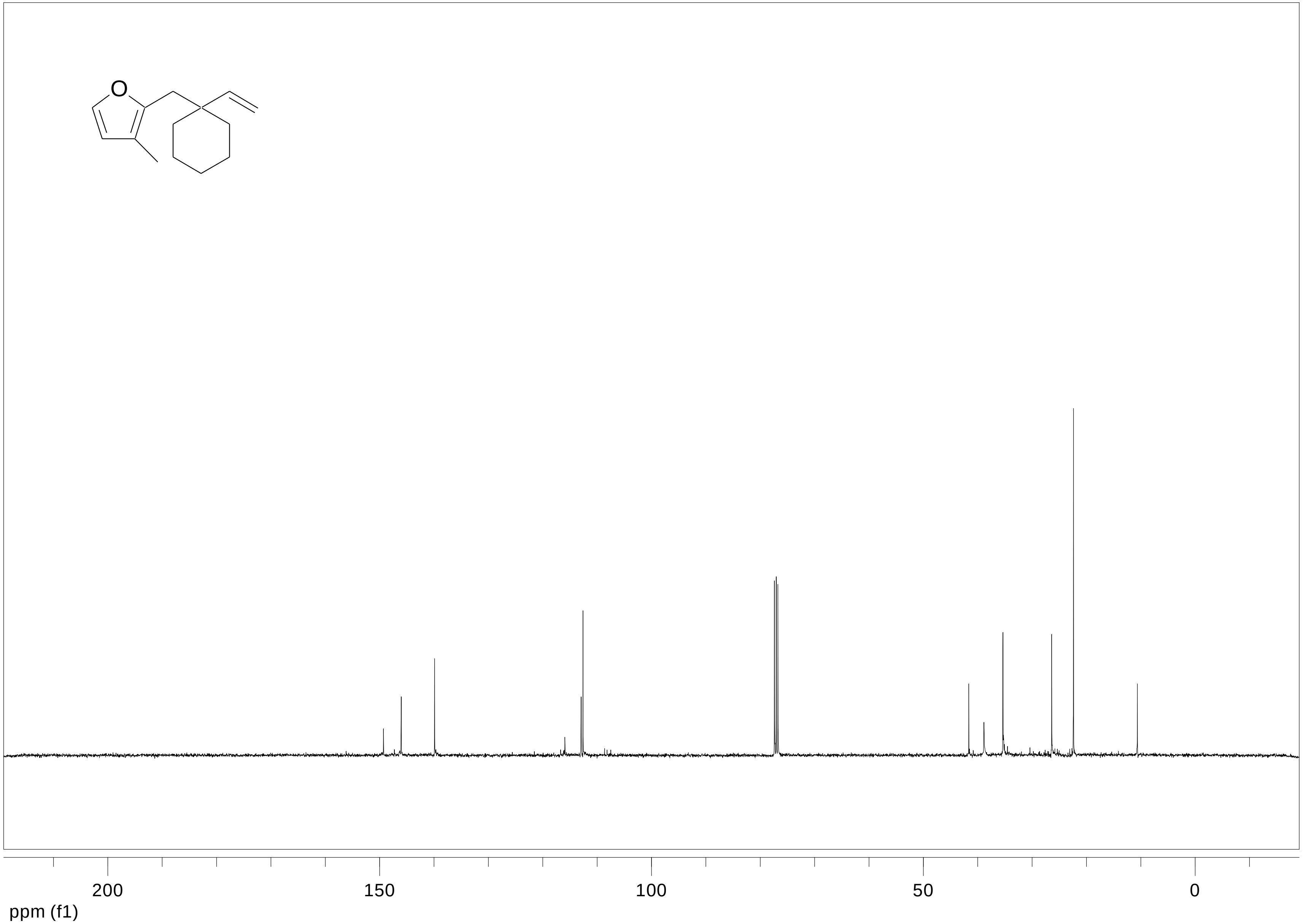

Compound 7g

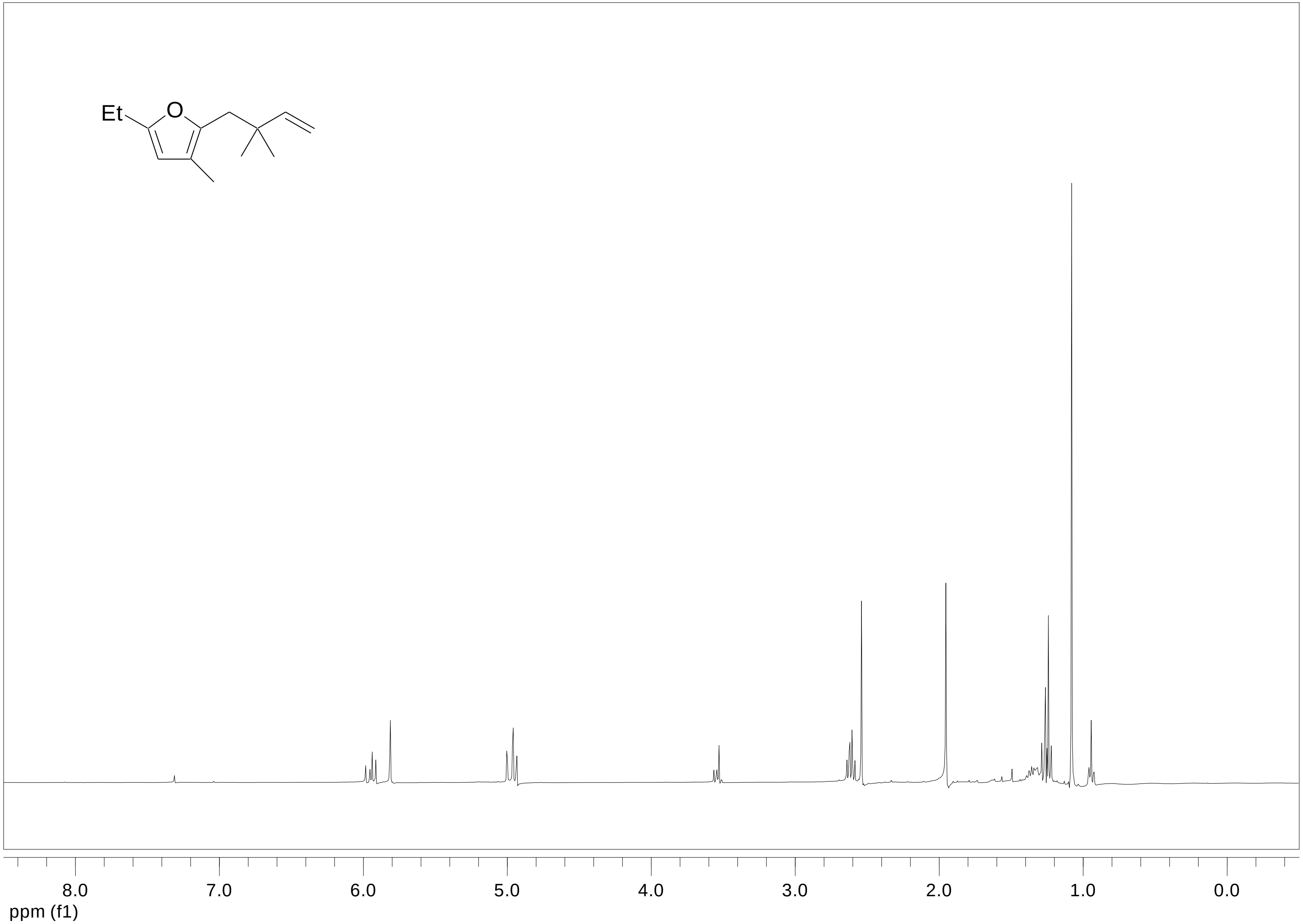

Compound 7h

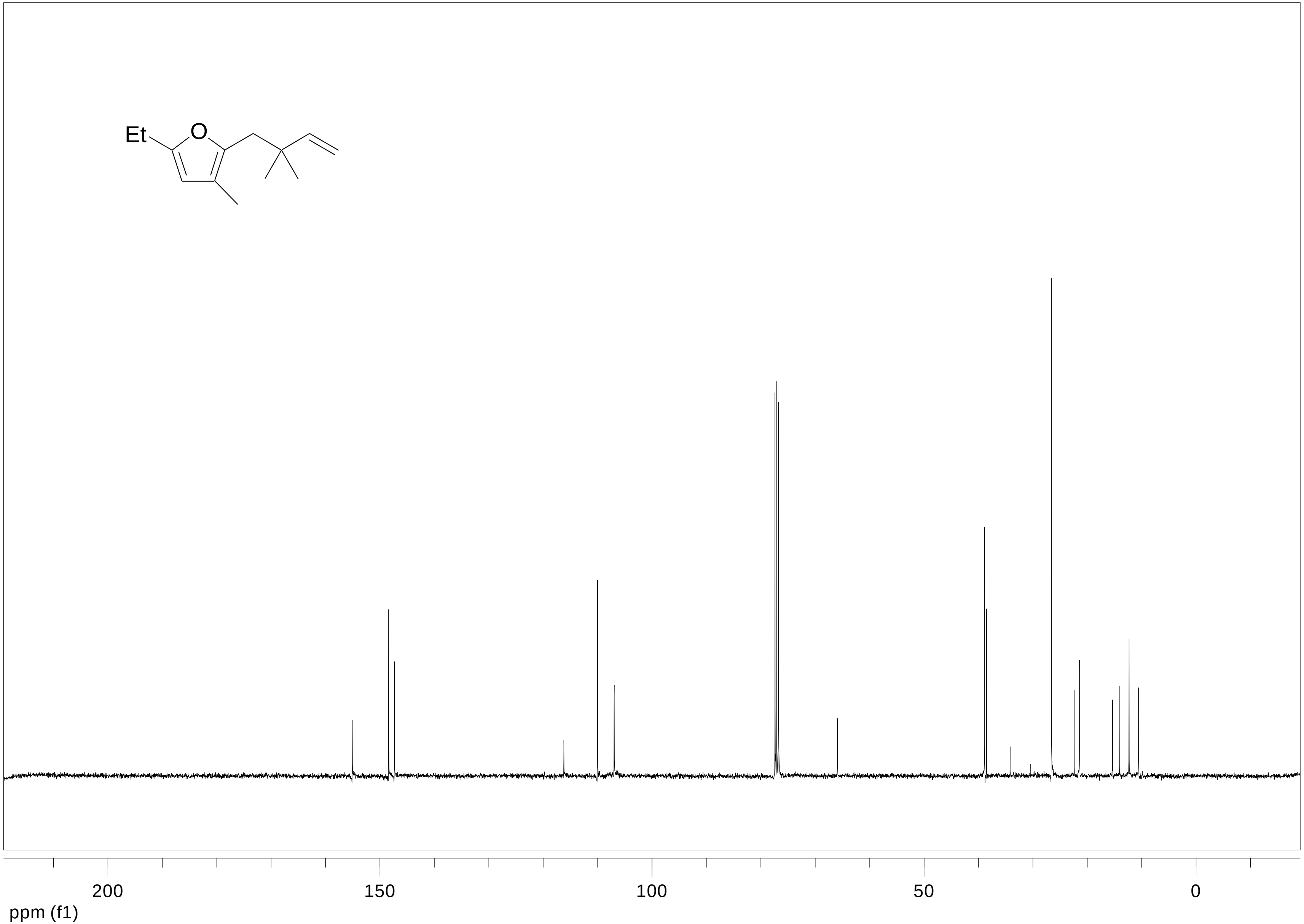

Compound 7h

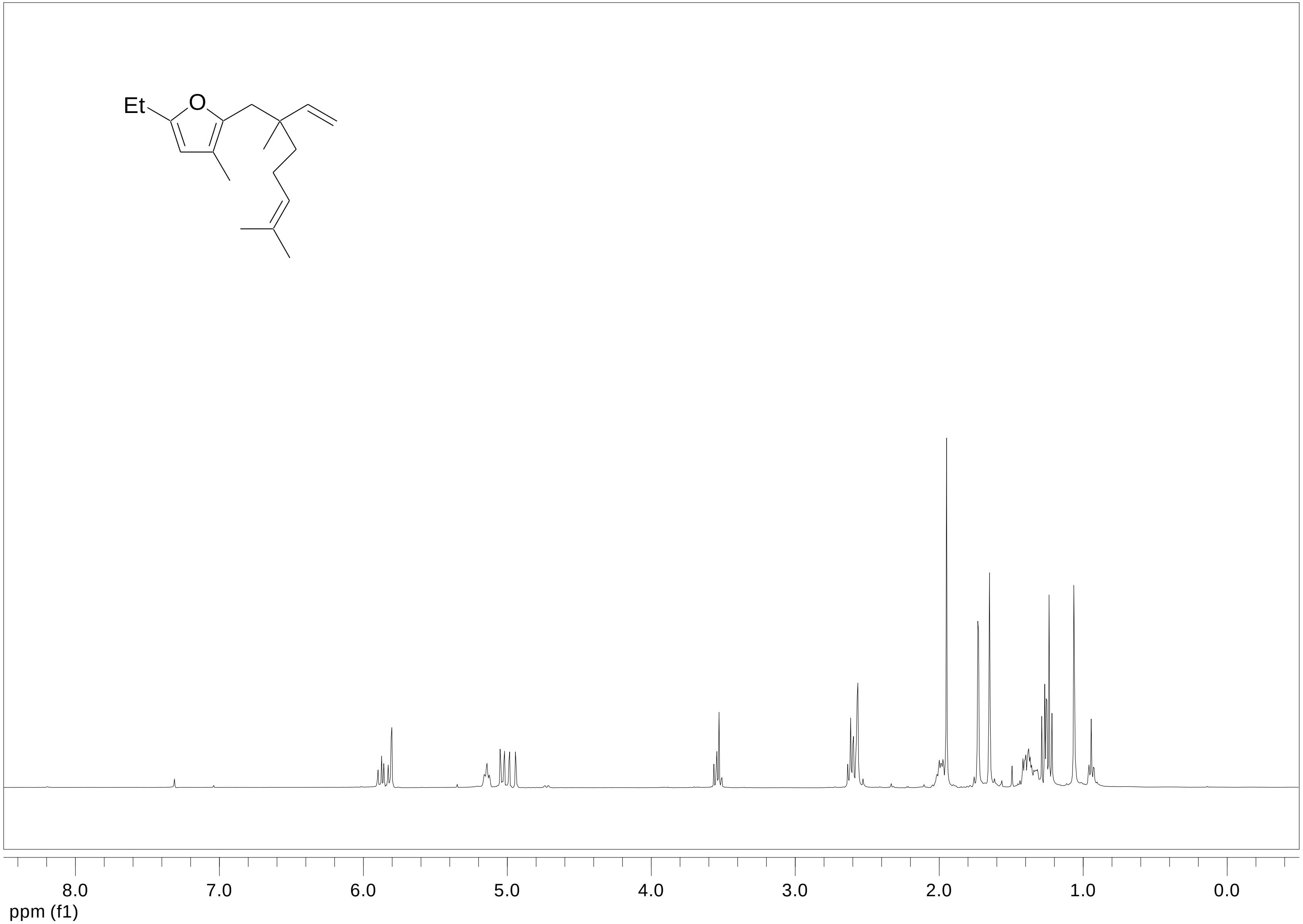

Compound 7i

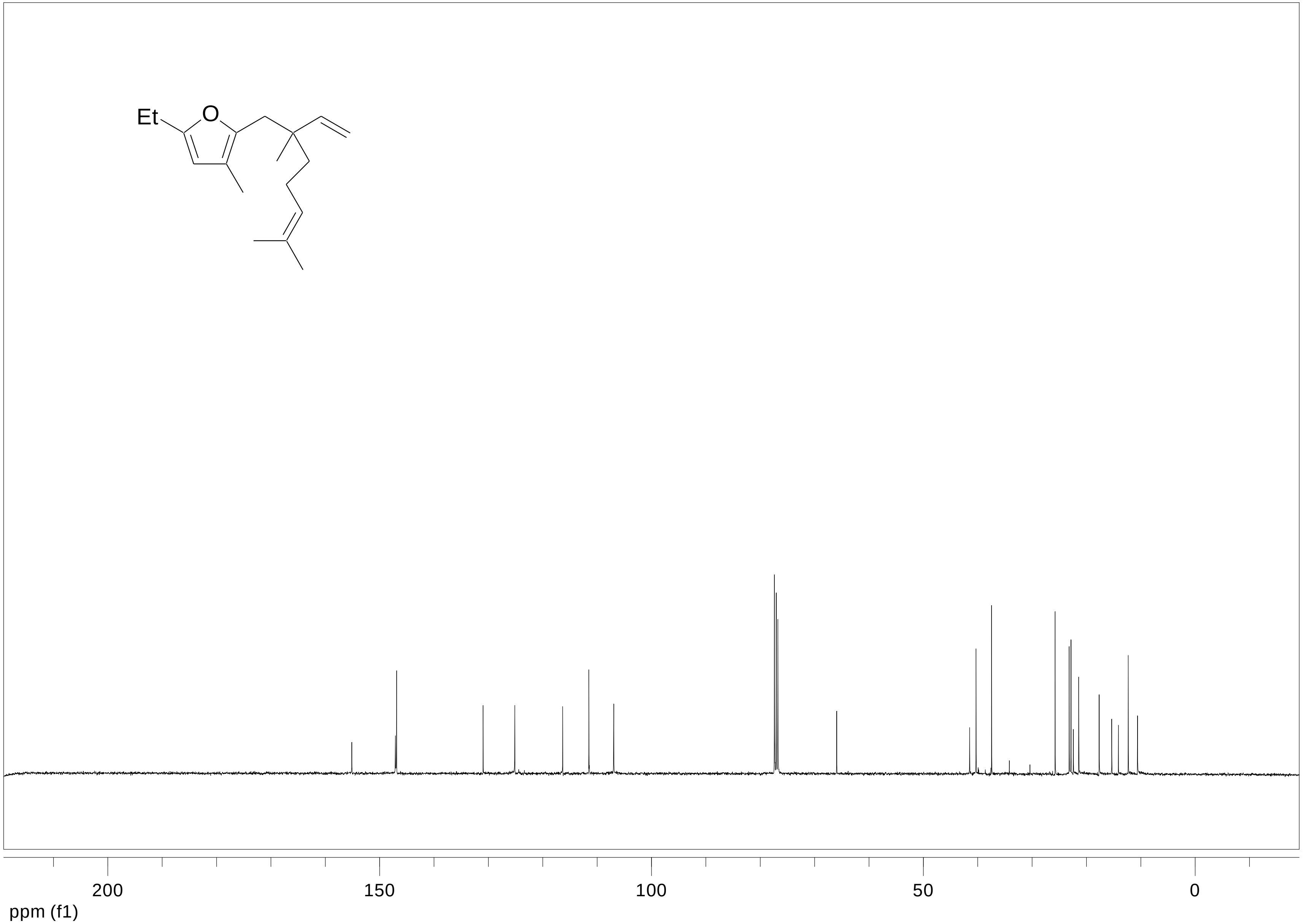

Compound 7i

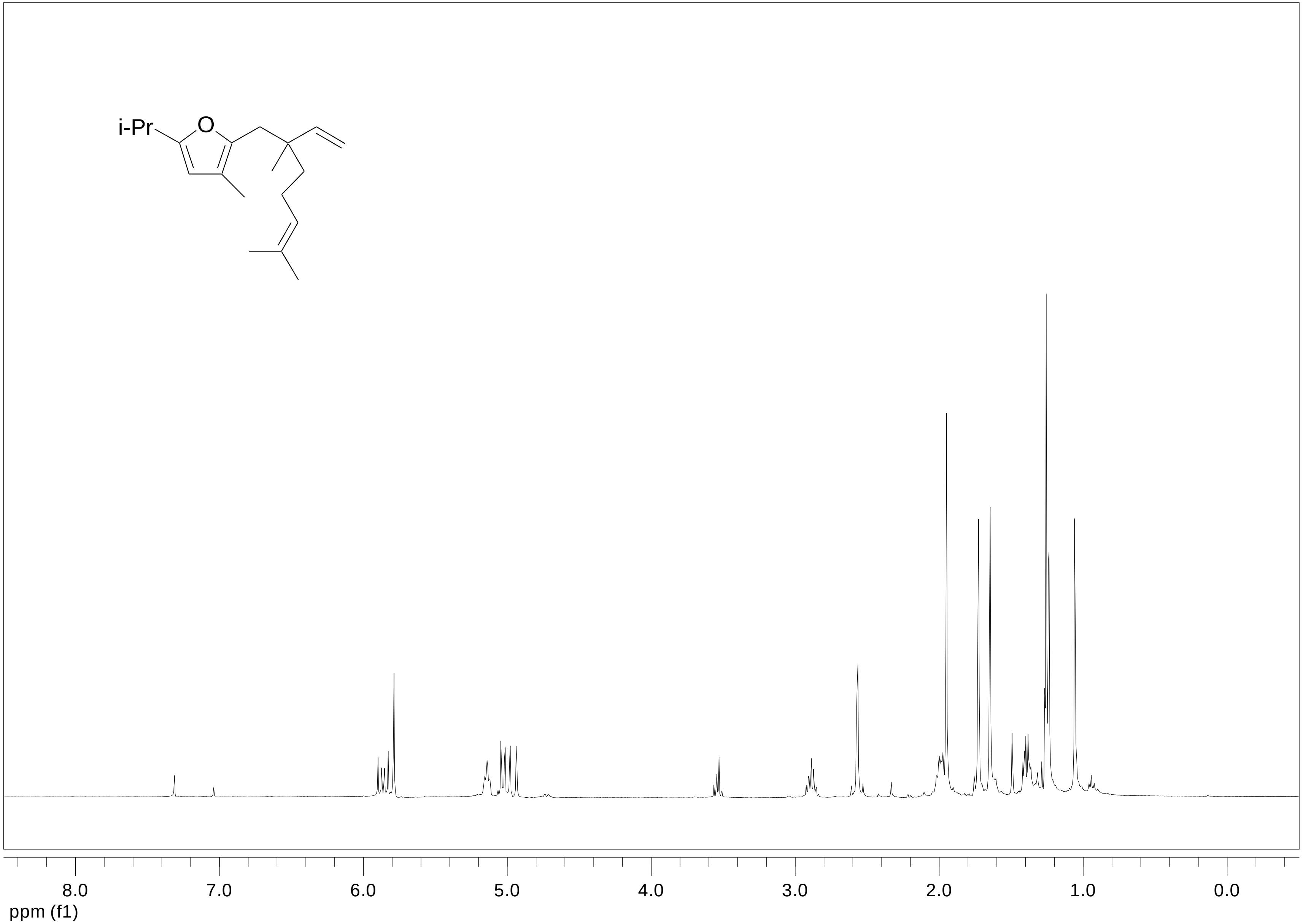

Compound 7j

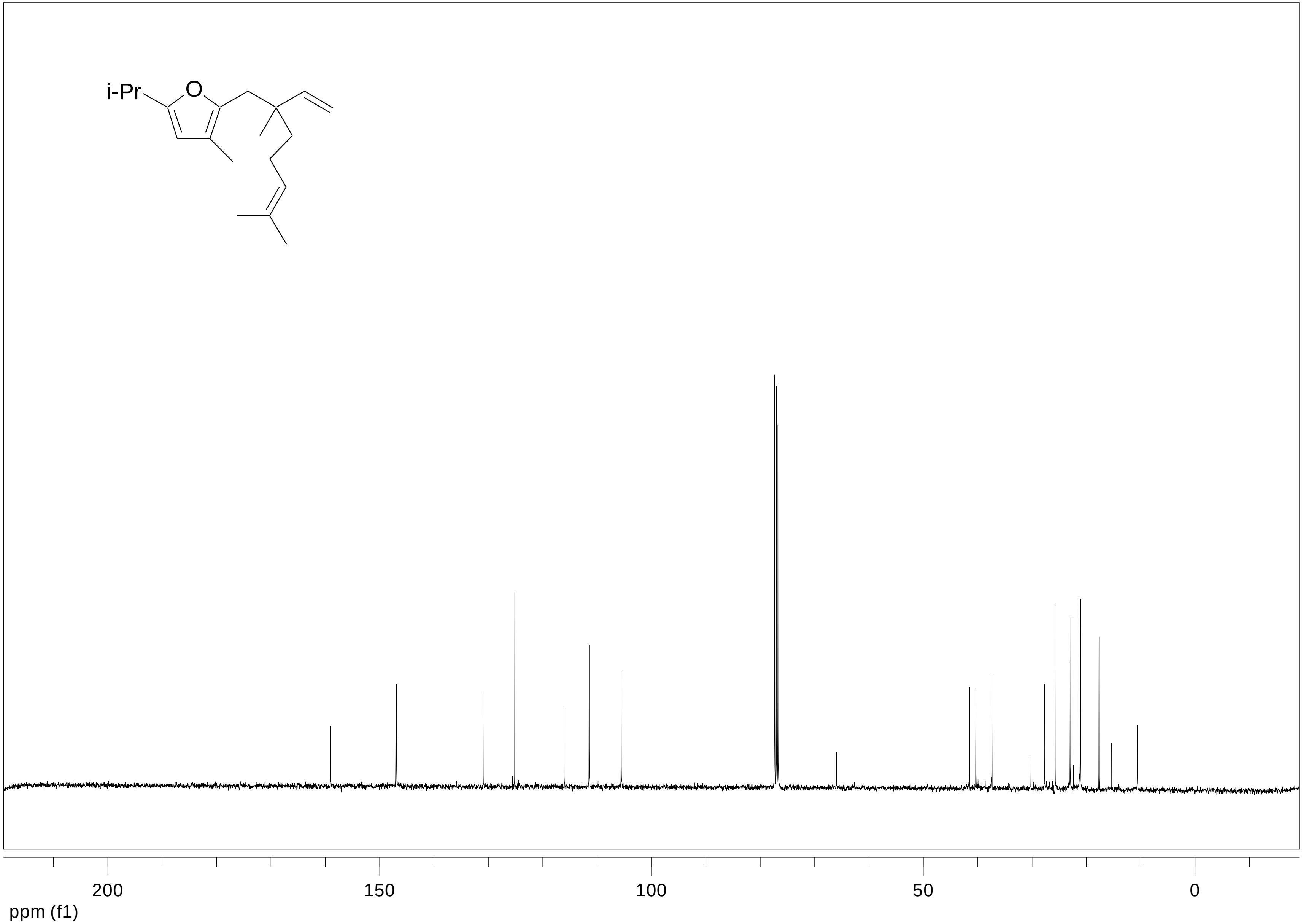

Compound 7j

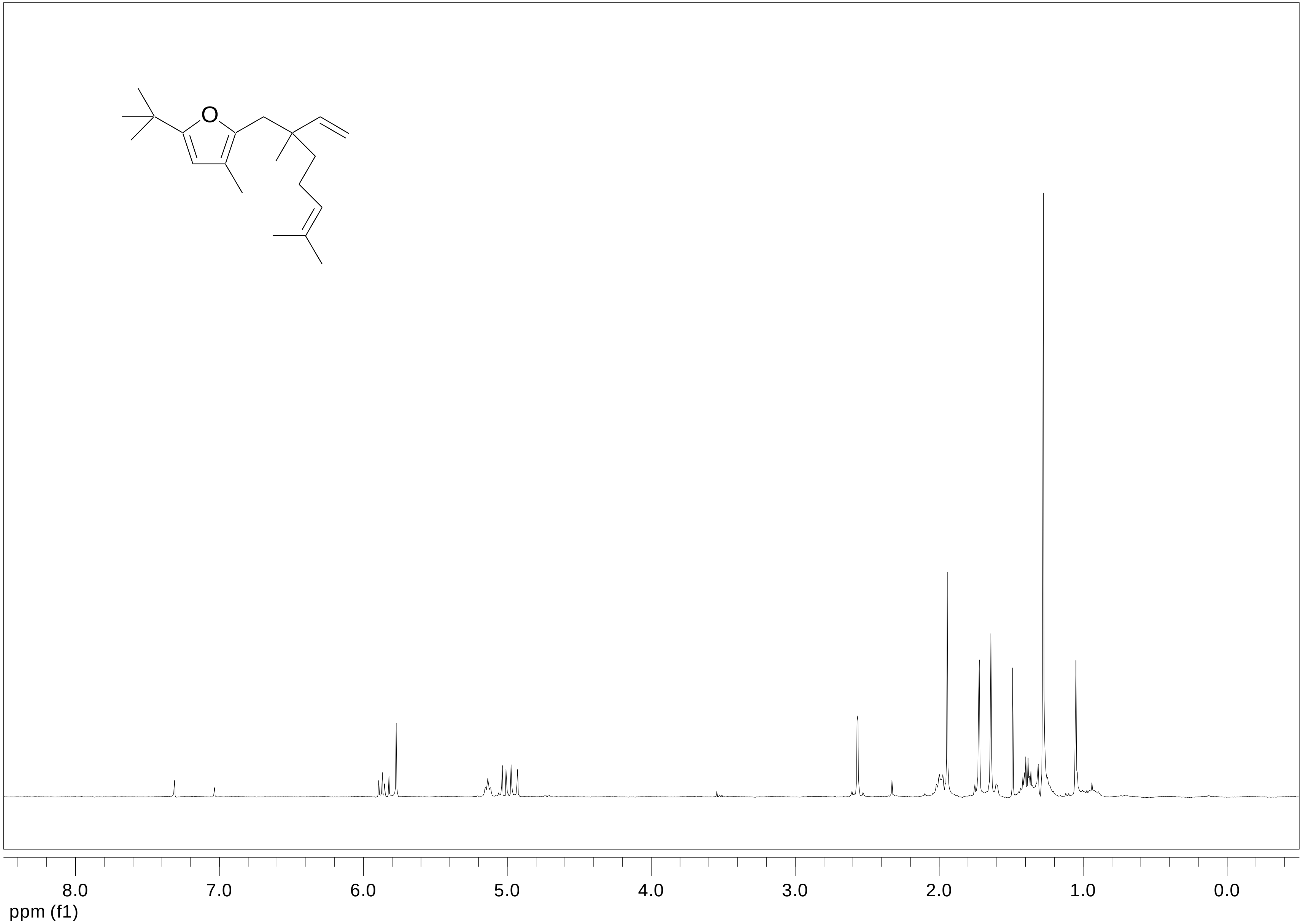

Compound 7k

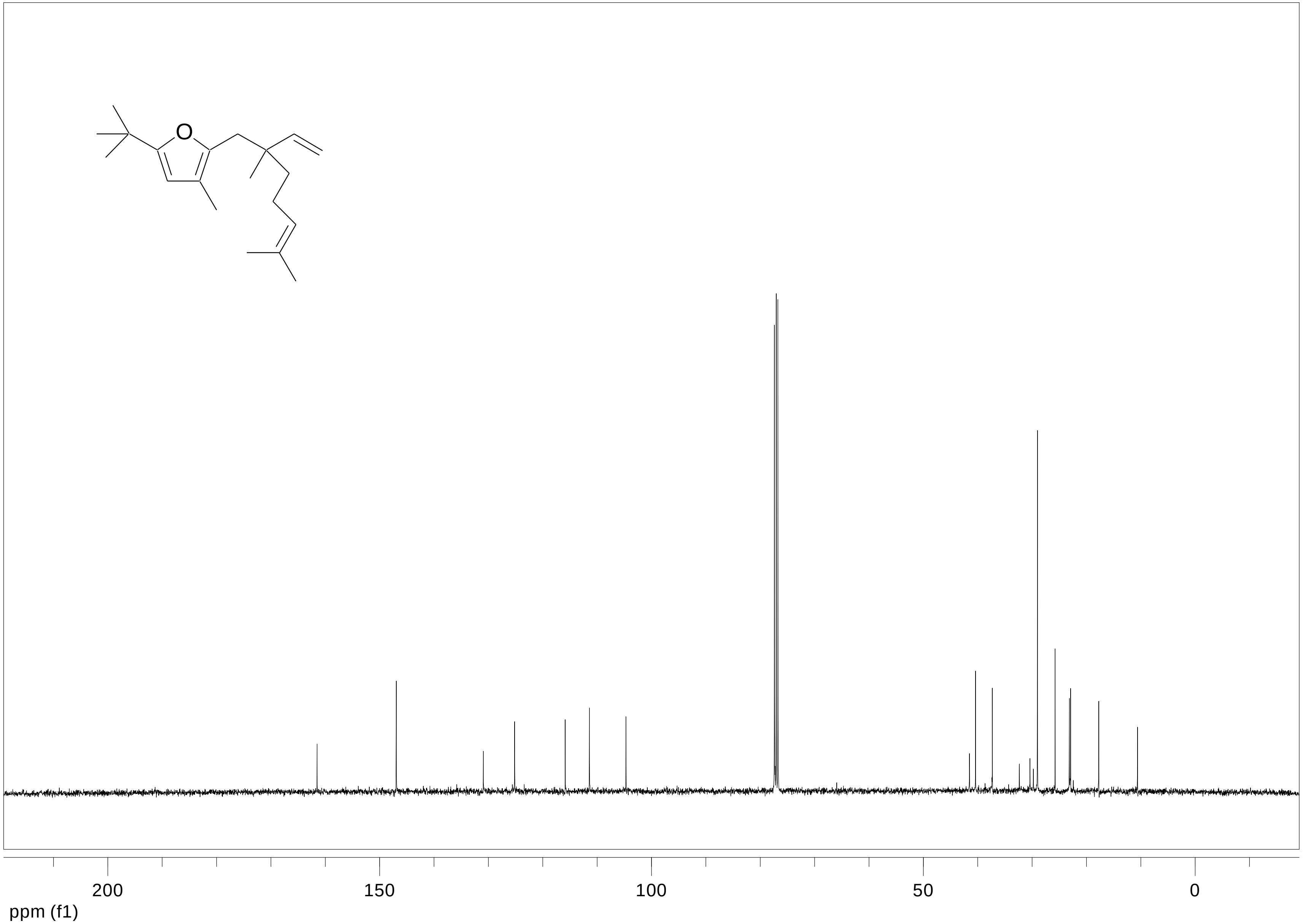

Compound 7k

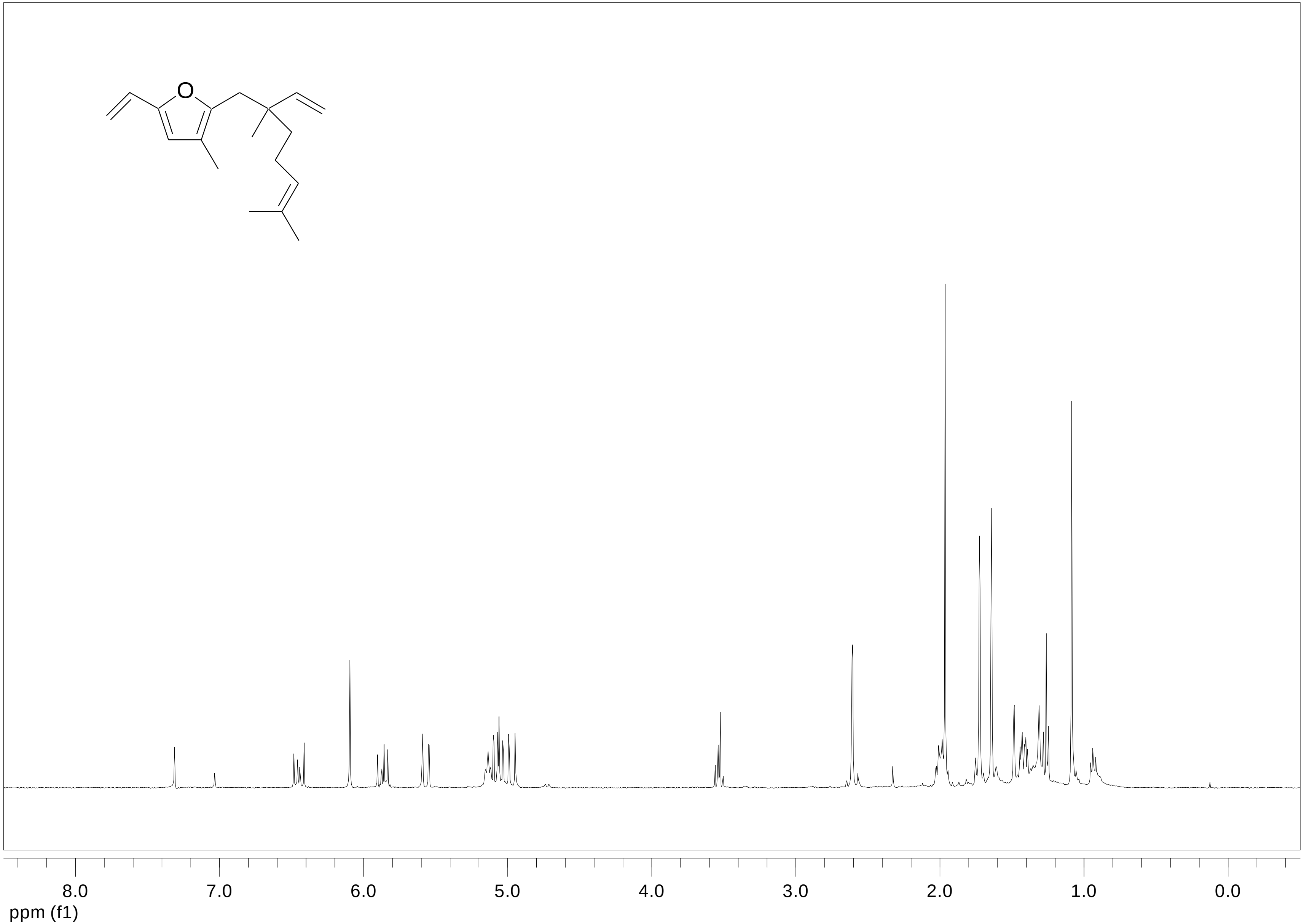

Compound 7l

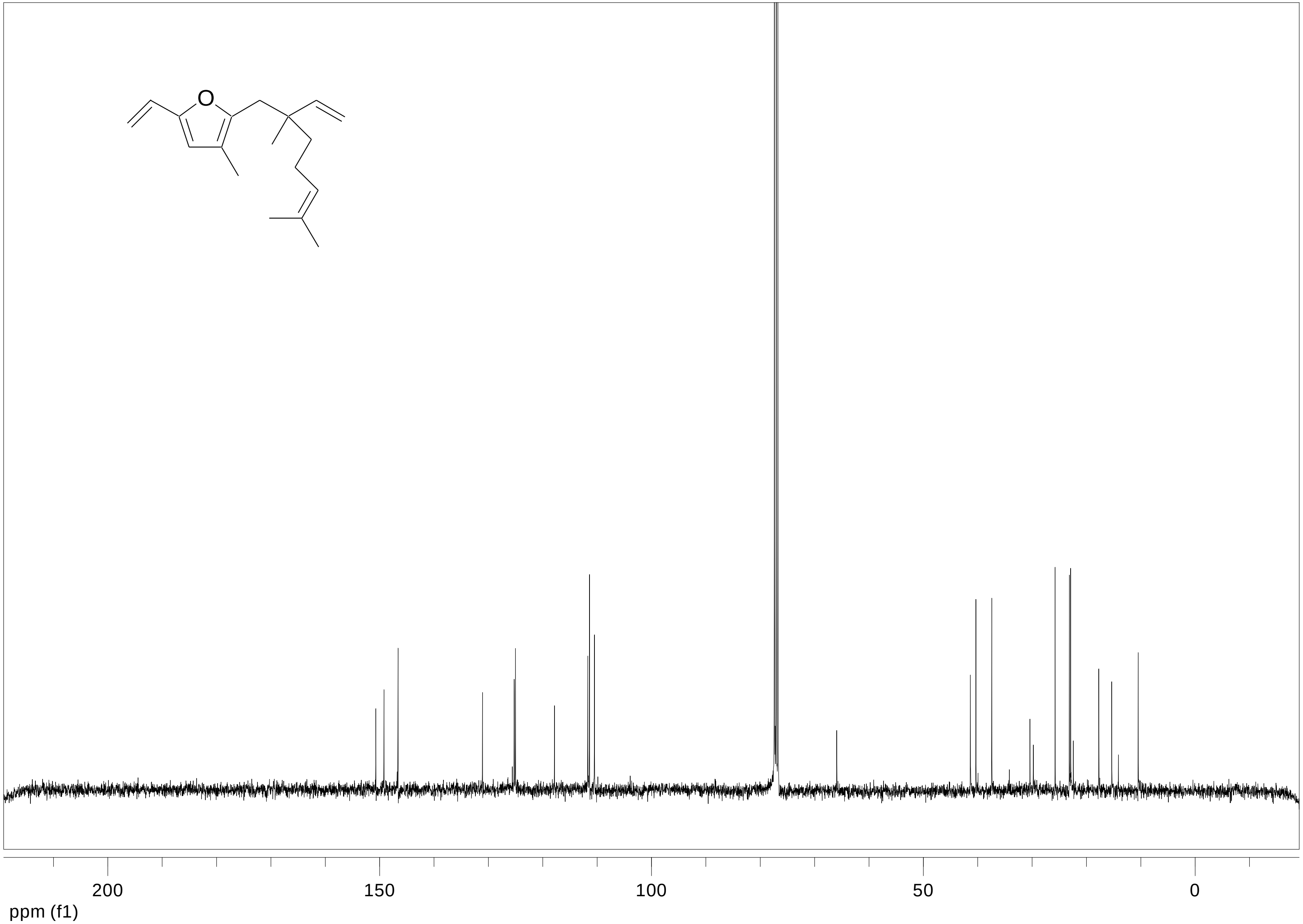

Compound 7l

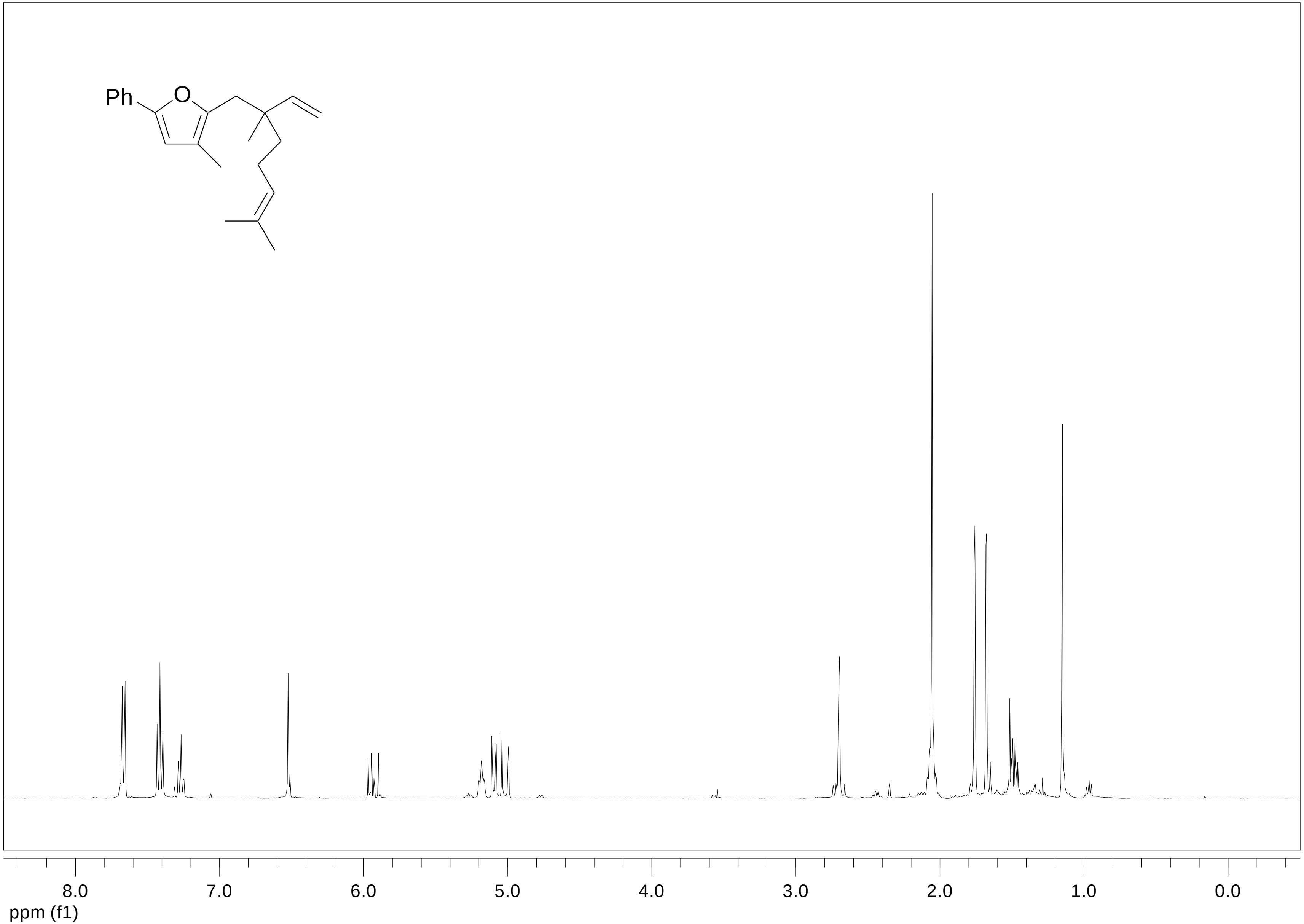

Compound 7m

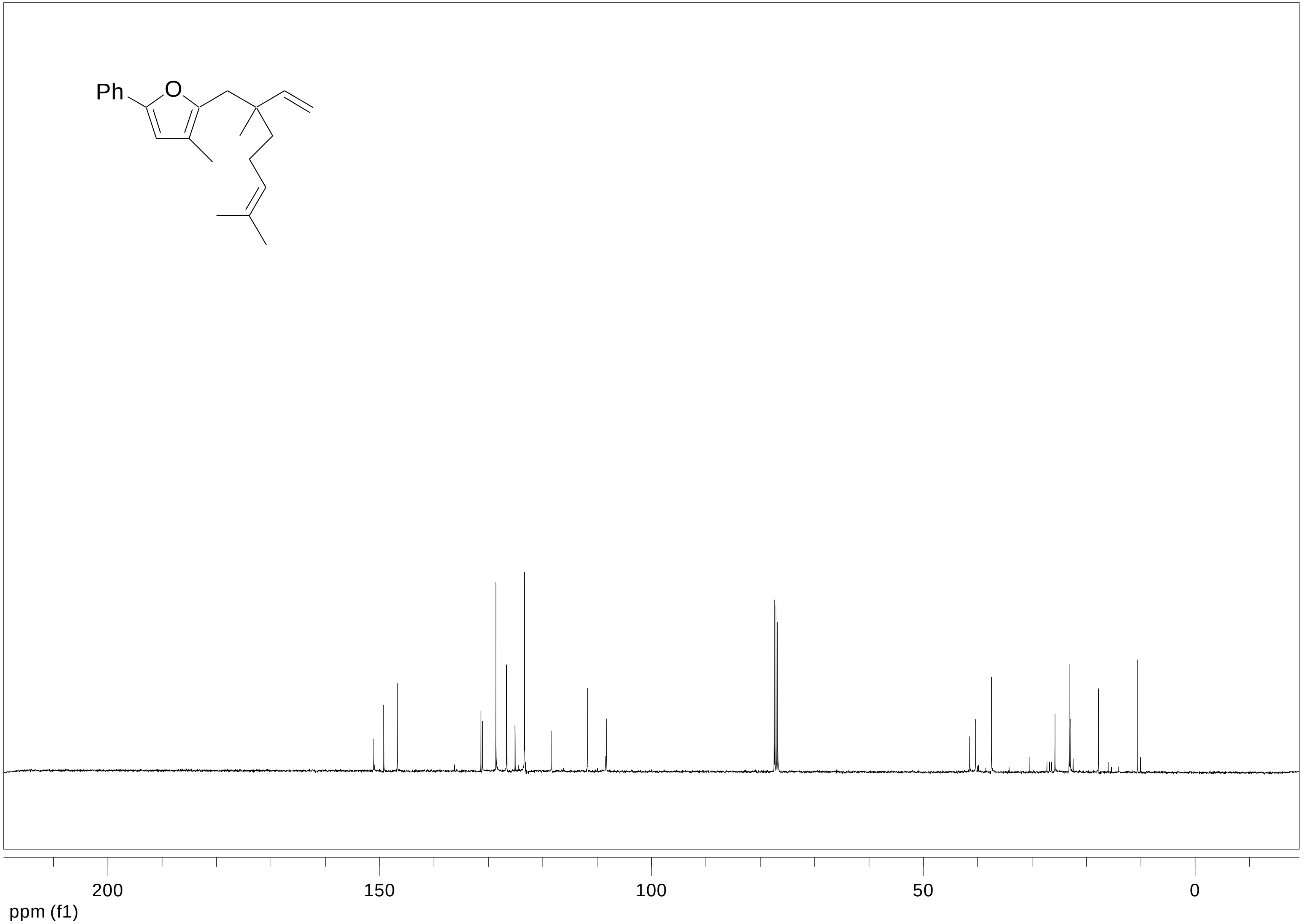

Compound 7m

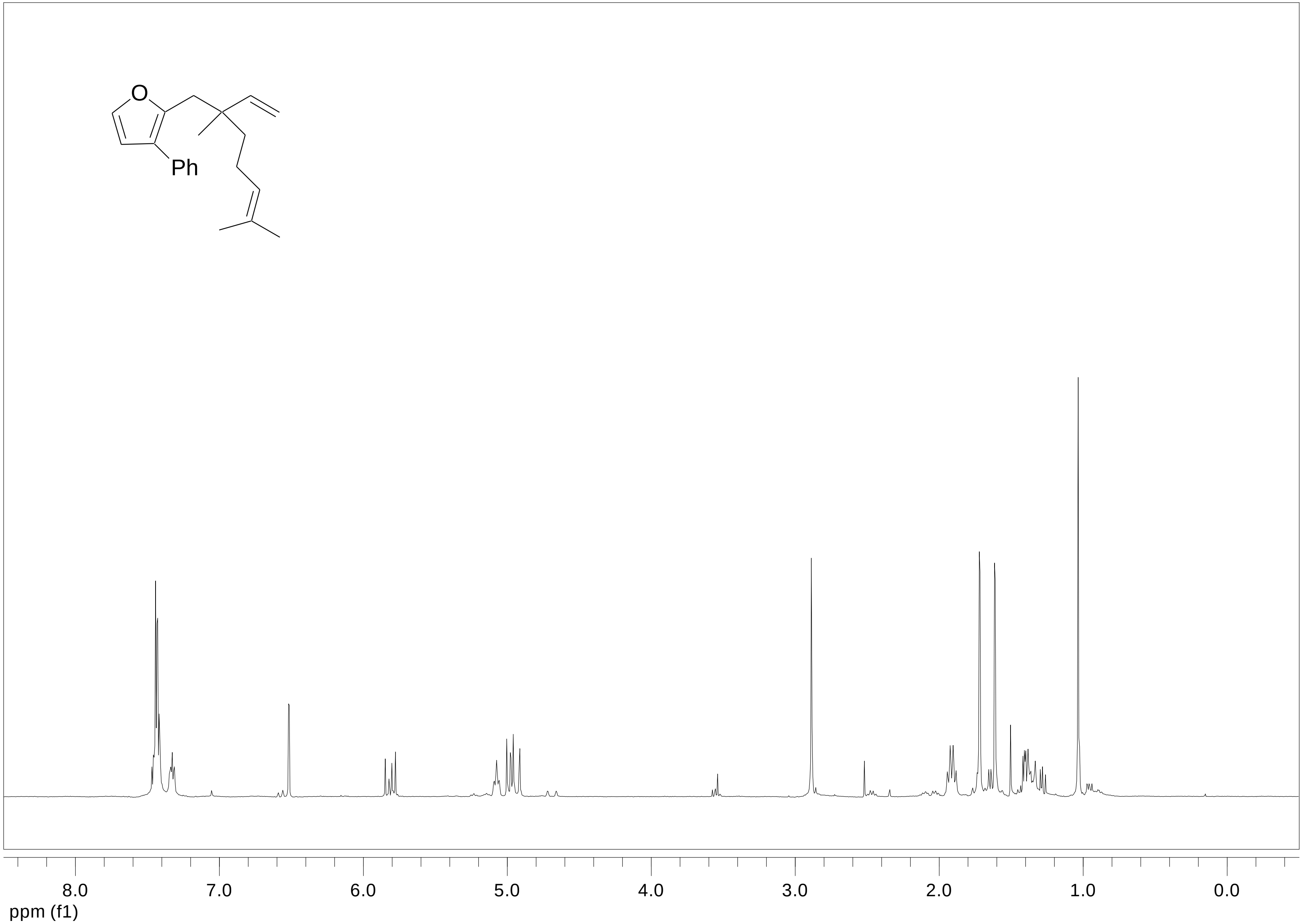

Compound 7n

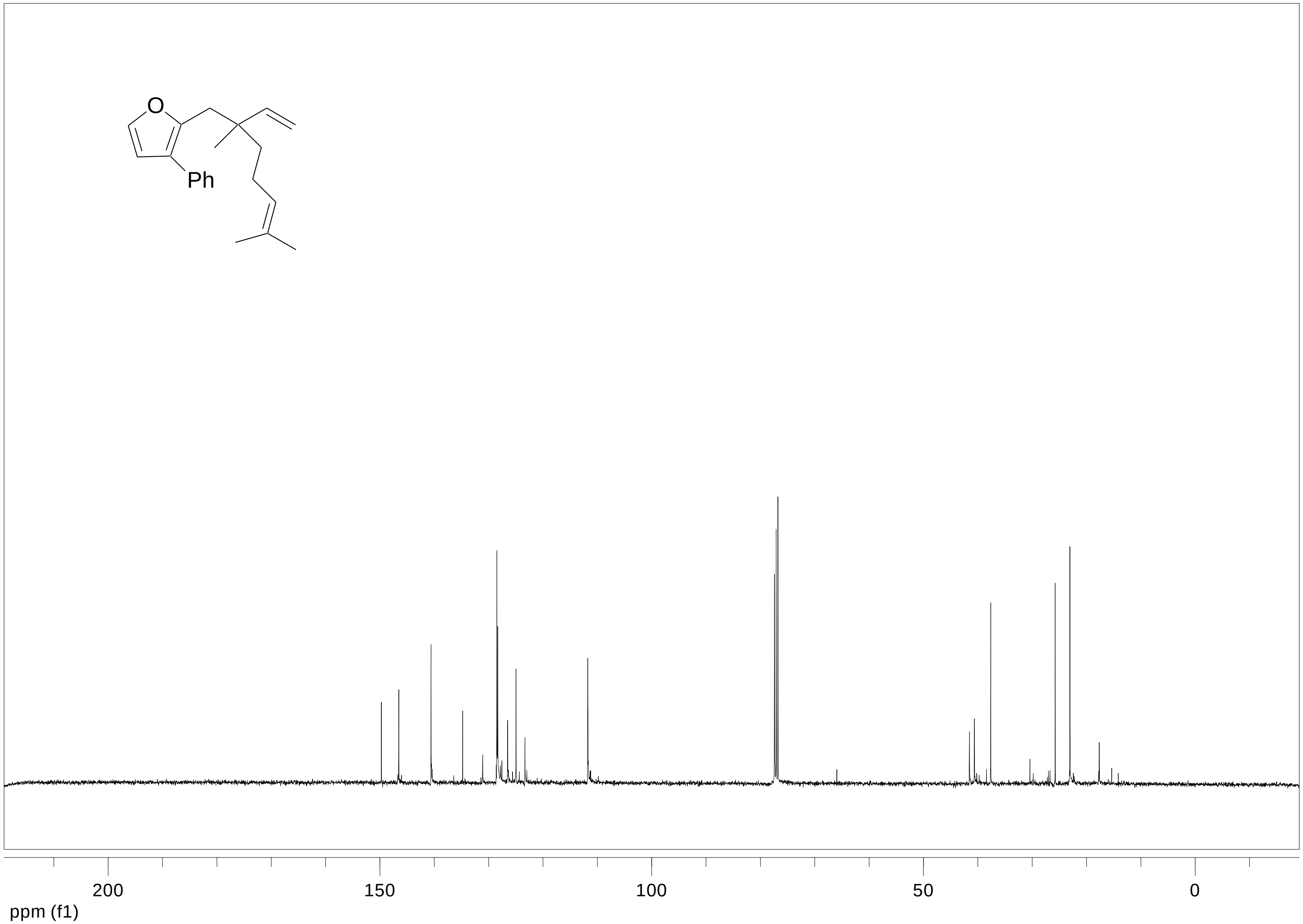

Compound 7n

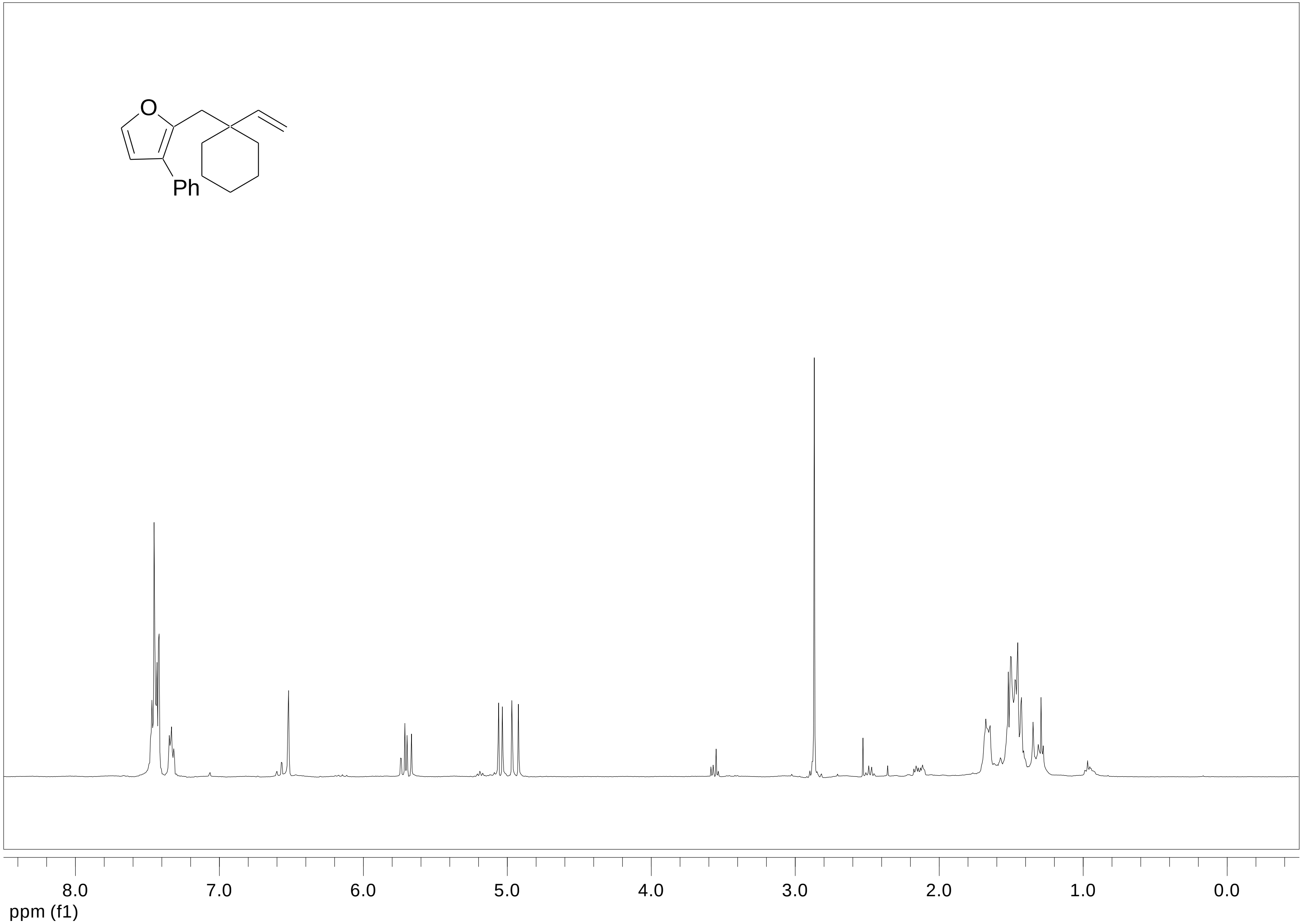

Compound 7o

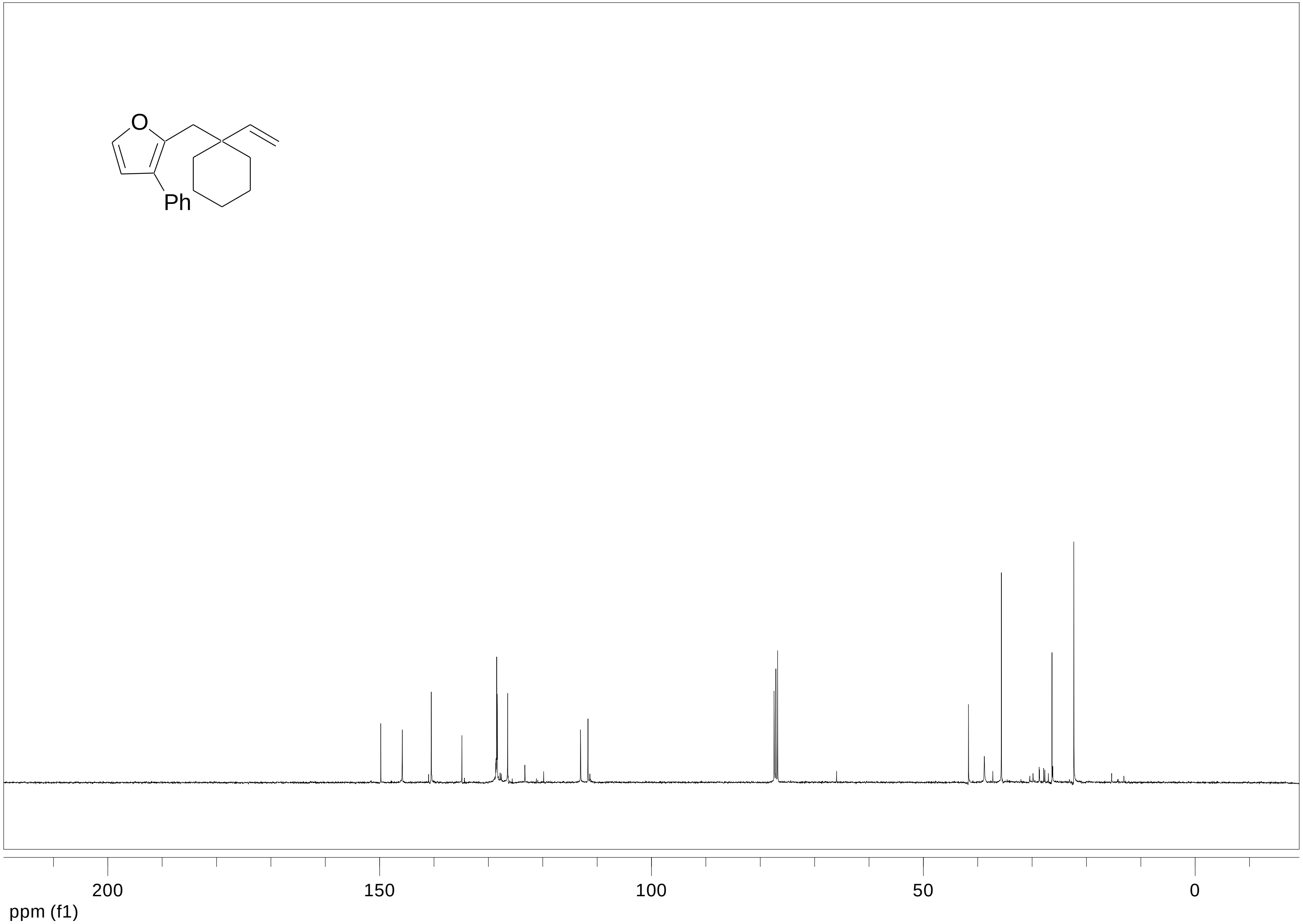

Compound 7o

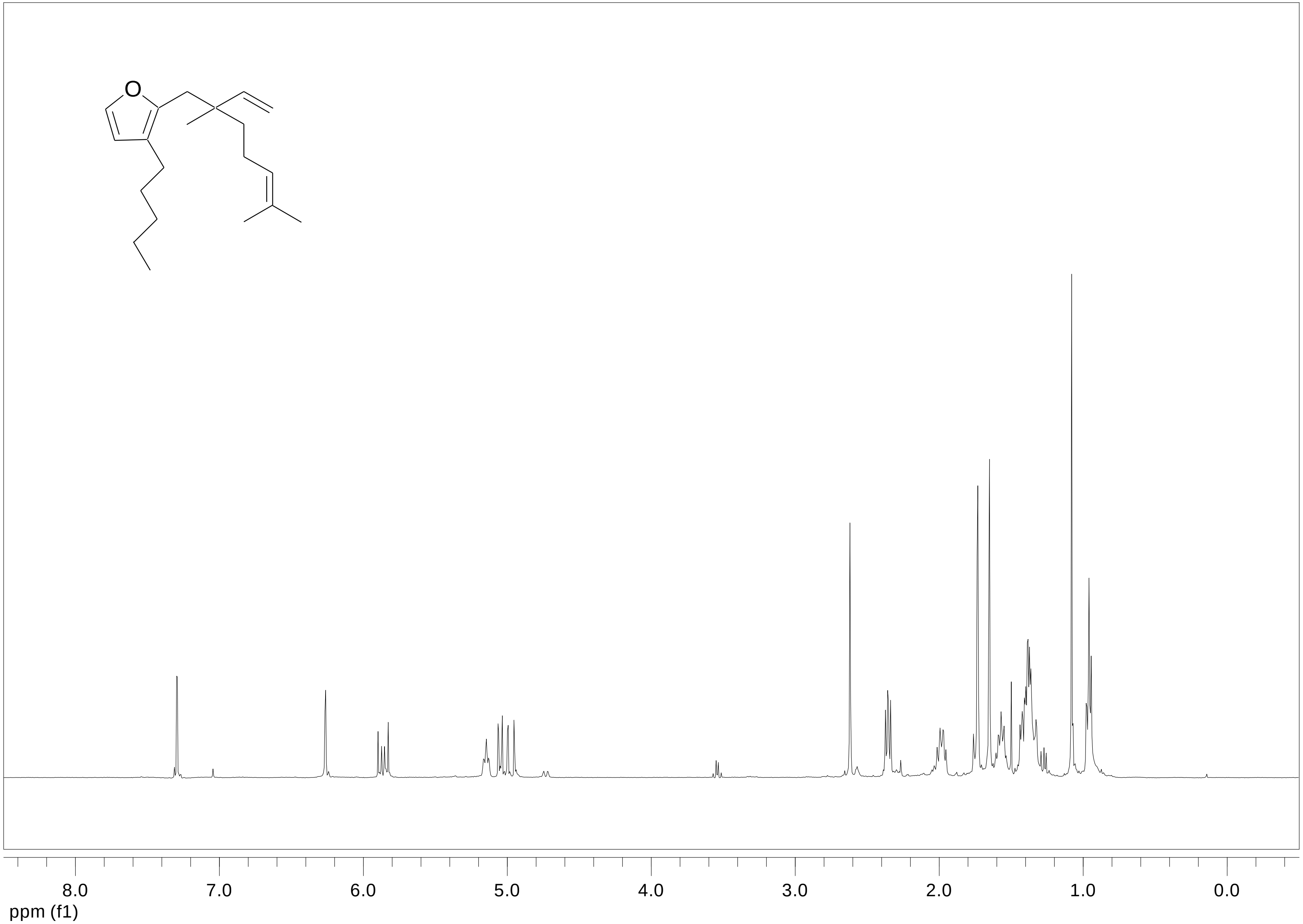

Compound 7p

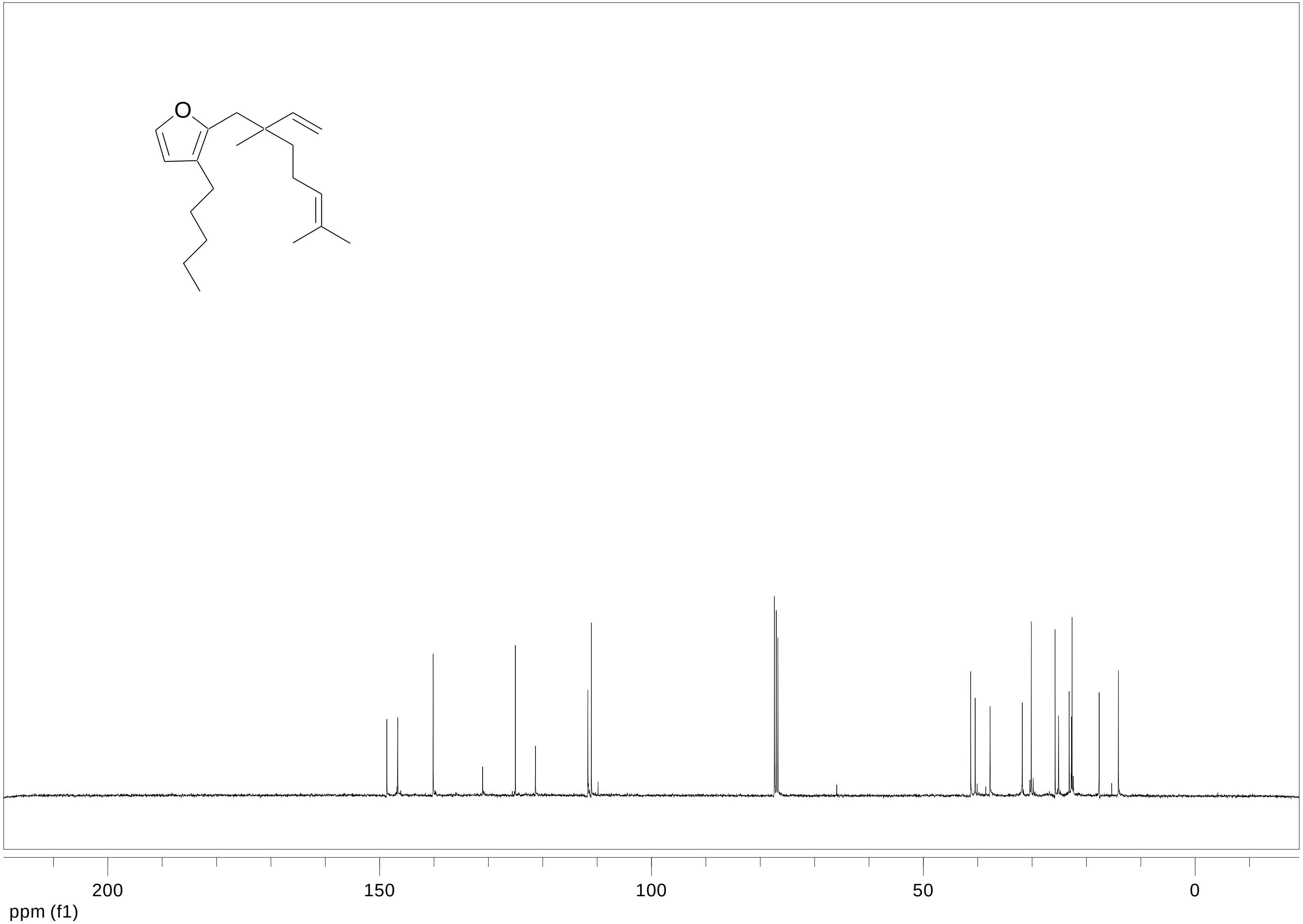

Compound 7p

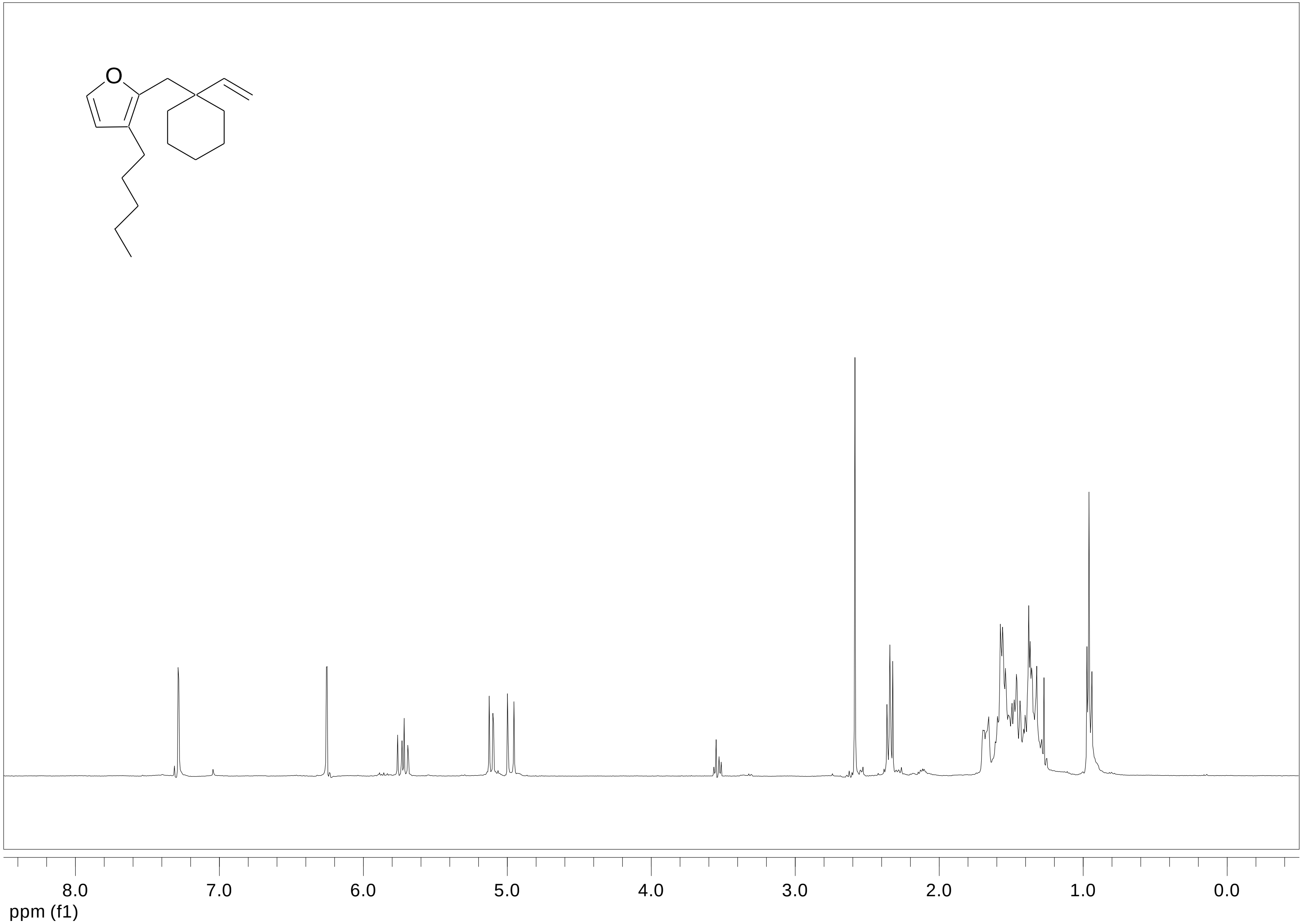

Compound 7q

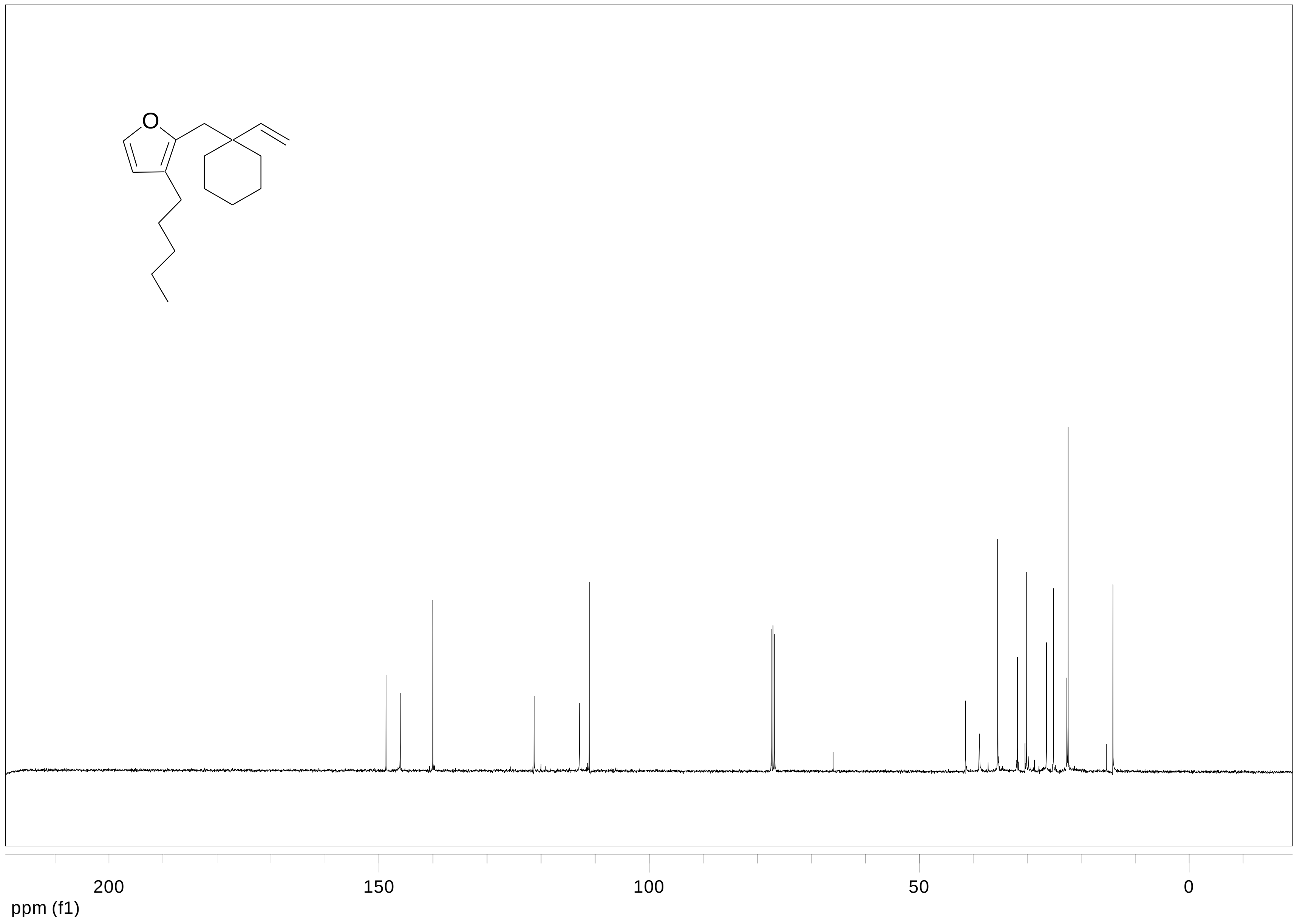

Compound 7q

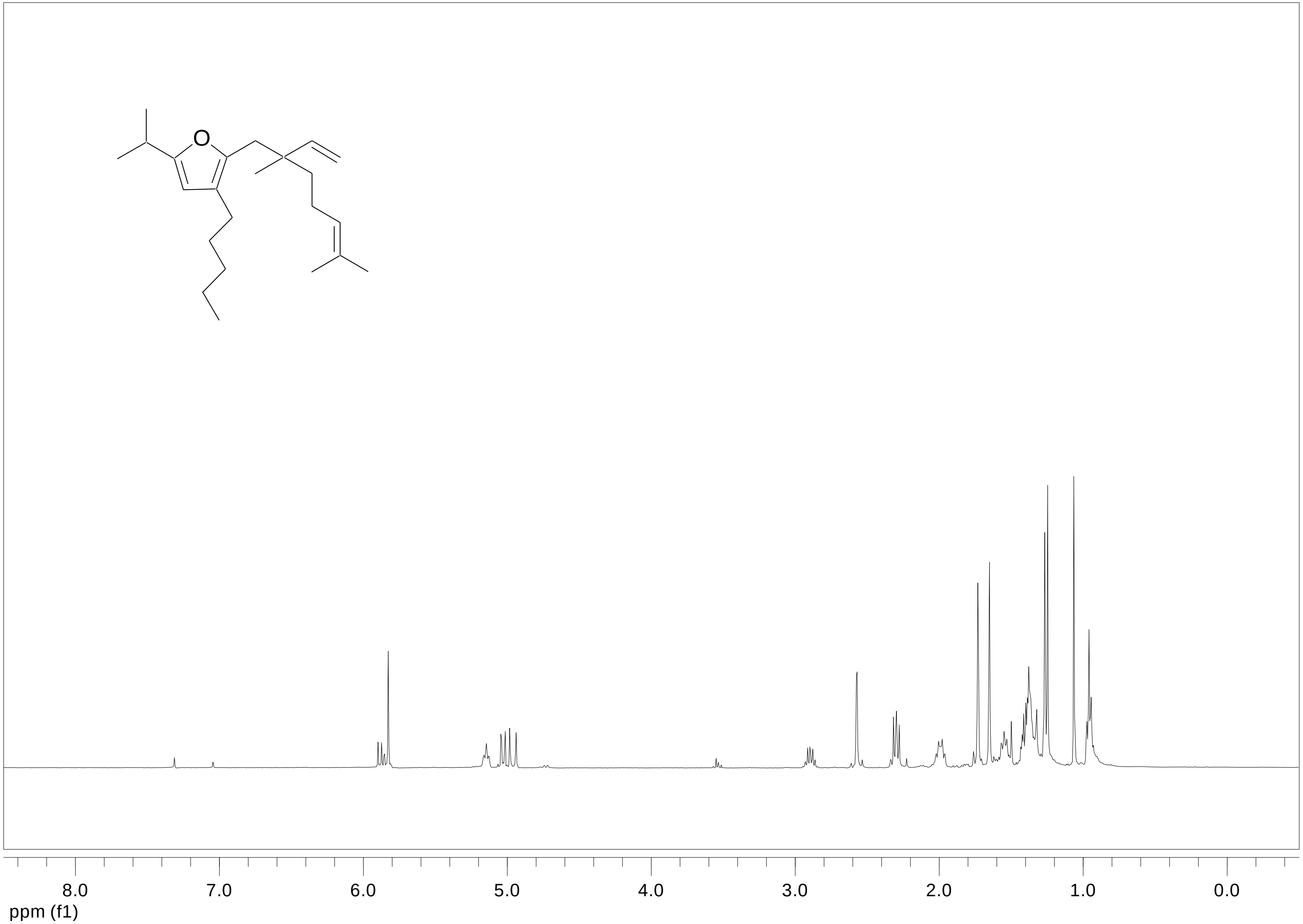

Compound 7r

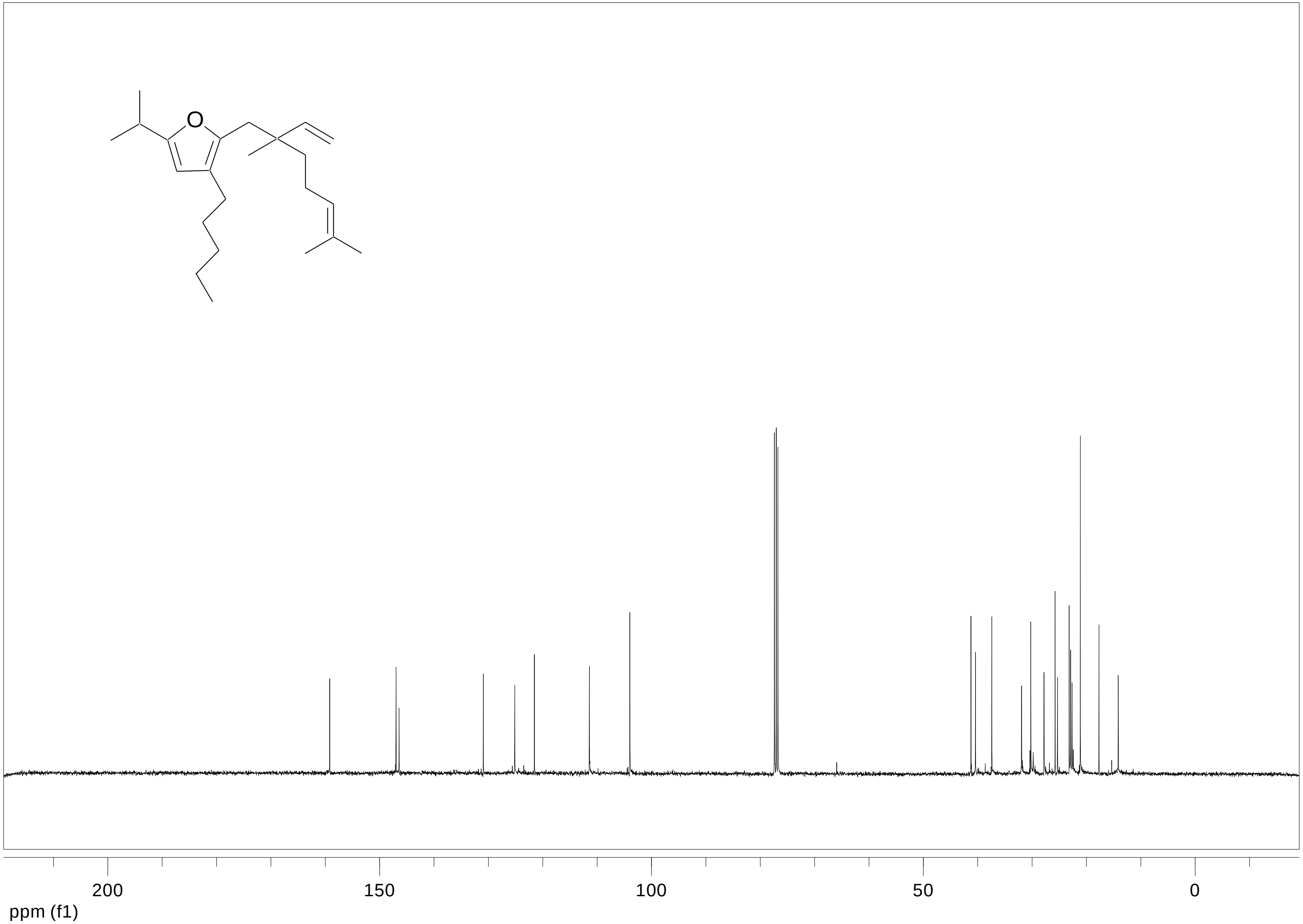

Compound 7r

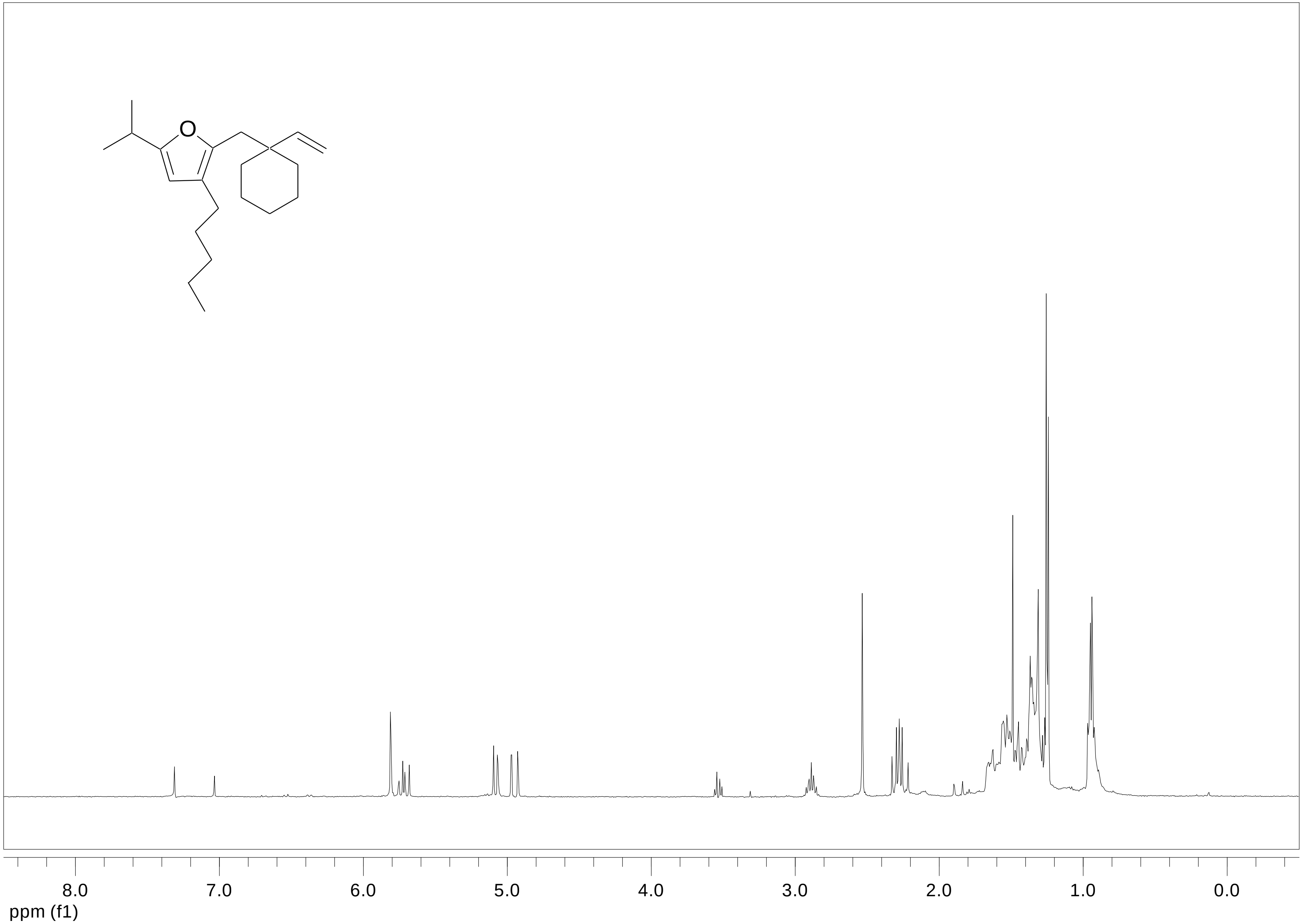

Compound 7s

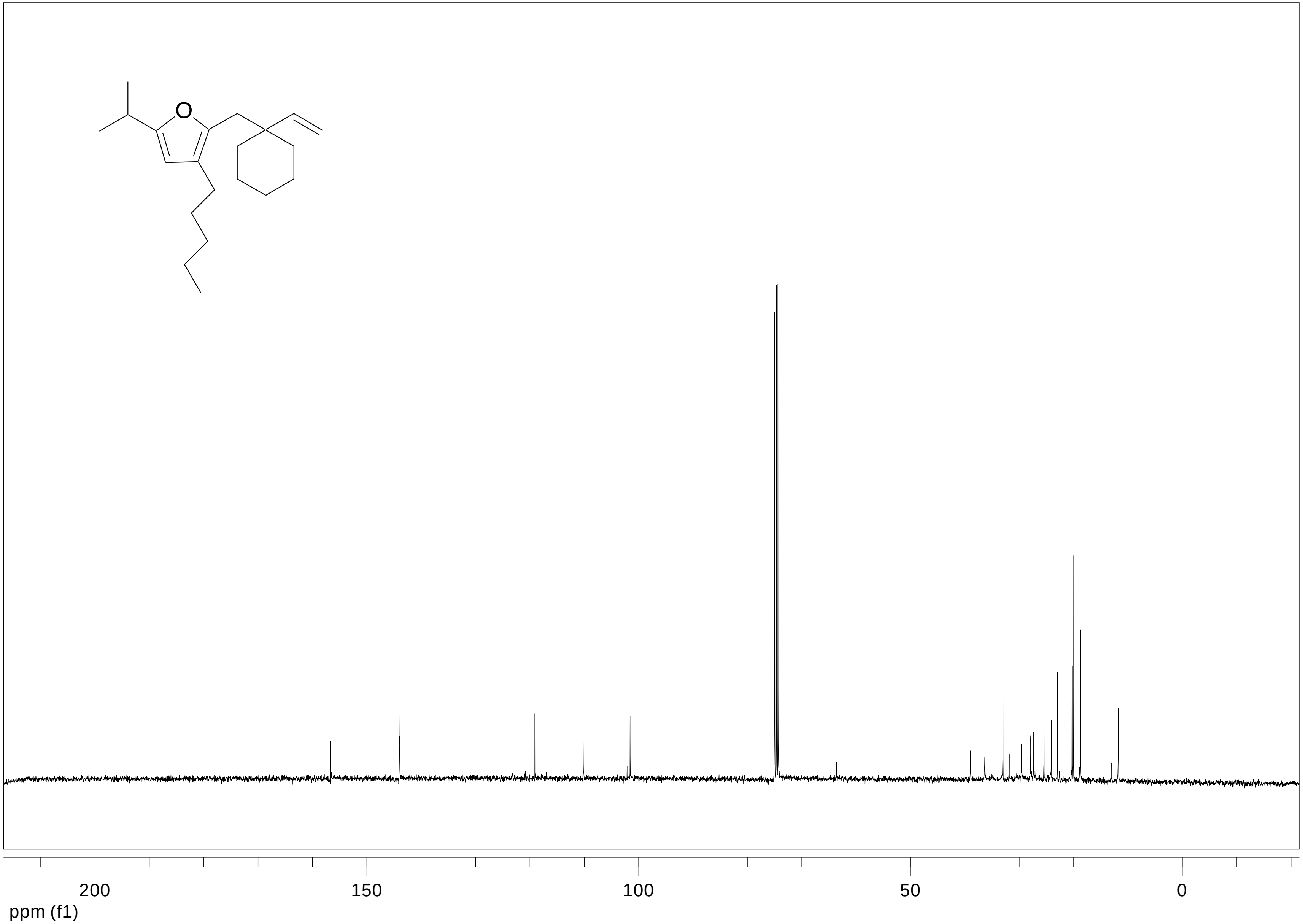

Compound 7s
